# Humans in Africa’s wet tropical forests 150 thousand years ago

**DOI:** 10.1038/s41586-025-08613-y

**Published:** 2025-02-26

**Authors:** Eslem Ben Arous, James A. Blinkhorn, Sarah Elliott, Christopher A. Kiahtipes, Charles D. N’zi, Mark D. Bateman, Mathieu Duval, Patrick Roberts, Robert Patalano, Alexander F. Blackwood, Khady Niang, Eugénie Affoua Kouamé, Edith Lebato, Emily Hallett, Jacopo N. Cerasoni, Erin Scott, Jana Ilgner, Maria Jesús Alonso Escarza, Francois Yodé Guédé, Eleanor M. L. Scerri

**Affiliations:** 1 Centro Nacional de Investigación sobre la Evolución Humana (CENIEH), Burgos, Spain; 2 Human Palaeosystems Group, Max Planck Institute of Geoanthropology (MPI-GEA), Jena, Germany; 3 Histoire Naturelle des Humanités Préhistoriques (HNHP), CNRS–Université de Perpignan Via Domitia (UPVD)–Muséum national d’Histoire naturelle (MNHN), Paris, France; 4 Department of Archaeology, Classics, and Egyptology, University of Liverpool, Liverpool, UK; 5 Department of Life and Environmental Sciences, Bournemouth University, Poole, UK; 6 University of South Florida Libraries, University of South Florida, Tampa, FL USA; 7 Départmente d’Histoire, Institut d’Histoire, d’Art et d’Archéologie Africains (IHAAA), Abidjan, Côte d’Ivoire; 8 School of Geography and Planning, University of Sheffield, Sheffield, UK; 9 Australian Research Centre for Human Evolution (ARCHE), Griffith University, Brisbane, Queensland Australia; 10 Palaeoscience Labs, Department of Archaeology and History, La Trobe University Melbourne Campus, Bundoora, Victoria Australia; 11 isoTROPIC Research Group, Max Planck Institute of Geoanthropology (MPI-GEA), Jena, Germany; 12 Department of Land Use and Urbanisation, Max Planck Institute of Geoanthropology (MPI-GEA), Jena, Germany; 13 Institute of Prehistoric Archaeology, University of Cologne, Cologne, Germany; 14 Department of Biological and Biomedical Sciences, School of Health and Behavioral Sciences, Bryant University, Smithfield, RI USA; 15 Human Evolution Research Institute (HERI), Department of Geological Sciences, University of Cape Town, Cape Town, South Africa; 16 Université Cheikh Anta Diop de Dakar, Dakar, Senegal; 17 Institut des Sciences Anthropologiques de Développement (ISAD), Abidjan, Côte d’Ivoire; 18 Department of Anthropology, Loyola University Chicago, Chicago, IL USA; 19 Department of Biology, Loyola University Chicago, Chicago, IL USA; 20 Department of Classics and Archaeology, University of Malta, Msida, Malta

**Keywords:** Archaeology, Archaeology

## Abstract

Humans emerged across Africa shortly before 300 thousand years ago (ka)^1–3^. Although this pan-African evolutionary process implicates diverse environments in the human story, the role of tropical forests remains poorly understood. Here we report a clear association between late Middle Pleistocene material culture and a wet tropical forest in southern Côte d’Ivoire, a region of present-day rainforest. Twinned optically stimulated luminescence and electron spin resonance dating methods constrain the onset of human occupations at Bété I to around 150 ka, linking them with *Homo sapiens*. Plant wax biomarker, stable isotope, phytolith and pollen analyses of associated sediments all point to a wet forest environment. The results represent the oldest yet known clear association between humans and this habitat type. The secure attribution of stone tool assemblages with the wet forest environment demonstrates that Africa’s forests were not a major ecological barrier for *H. sapiens* as early as around 150 ka.

## Abstract (web summary)

The identification of tools dated to the time of *Homo sapiens* associated with microfloral evidence of wet tropical forests indicates that West African forests were occupied by humans much earlier in human evolution than previously thought.

## Main

Our species (*Homo sapiens*) is thought to have emerged shortly before 300 thousand years ago (ka) in Africa, before dispersing to occupy all the world’s biomes, from deserts to dense tropical rainforest^4,5^. Although grasslands and coasts have typically been given primacy in studies of the cultural and environmental context for human emergence and spread (for example, refs. ^6–8^), recent evidence has implicated several regions and ecosystems in the earliest prehistory of our species^3,9,10^. Rainforest habitation in Asia and Oceania is firmly documented as early as 45 ka (refs. ^11,12^), and perhaps as early as 73 ka (ref. ^13^). However, the oldest secure, close human associations with such wet tropical forests in Africa do not date beyond around 18 ka (refs. ^6,14,15^), despite evidence of the widespread presence of Middle Stone Age (MSA) assemblages in regions of present-day African rainforest^16,17^ (Supplementary Information Section SI-1). Isotopic and zooarchaeological evidence from Panga ya Saidi in Kenya have supported an earlier use of mixed tropical forest and ecotonal environments from at least 77 ka (refs. ^18,19^), but clear evidence for the dedicated occupation of wet tropical forest remains lacking.

**Fig. 1:**
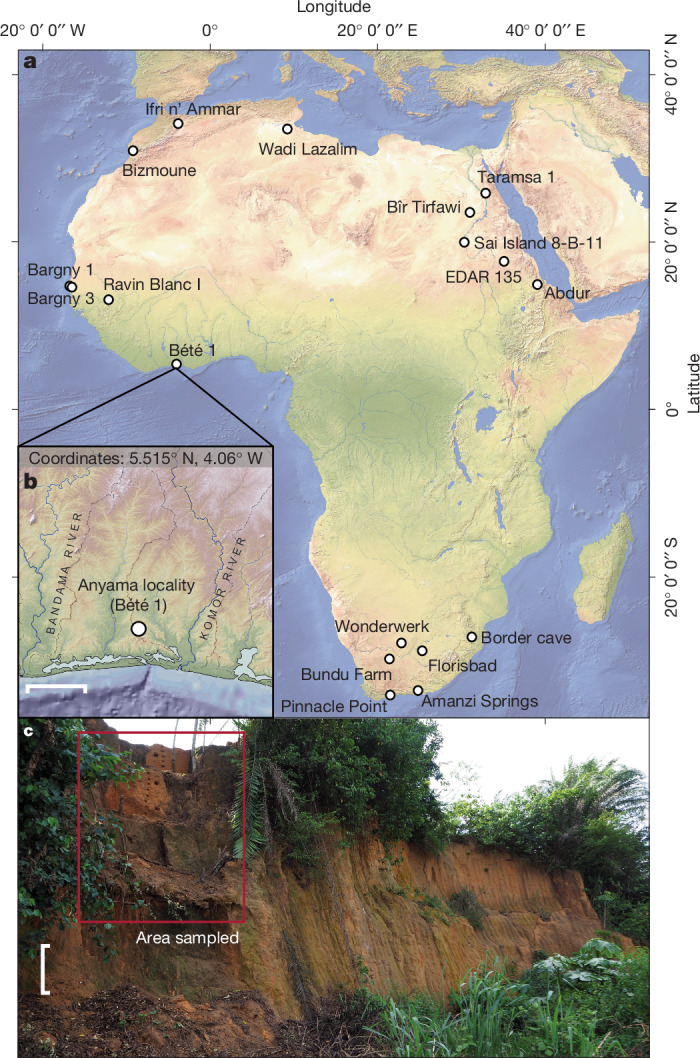
The Bété I site. **a**, General map showing the African sites dated to MIS 6 (around 130–190 ka). **b**, Location of Bété I site. **c**, Sequence at Bété I in 2020 after sampling for geochronology and palaeoecological proxies.

**Fig. 2:**
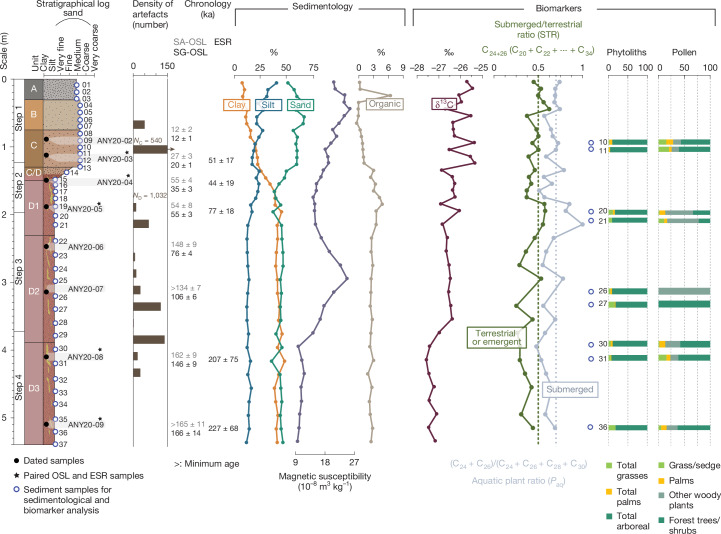
Stratigraphy, artefact density, geochronology, sedimentology and biomarkers from the Bété I sequence. Artefact densities are derived from ref. ^21^. Numerical age uncertainties are given at 1*σ*. NC and ND are the total numbers of lithics artefacts found in Units C and D, respectively.

**Extended Data Fig. 1:**
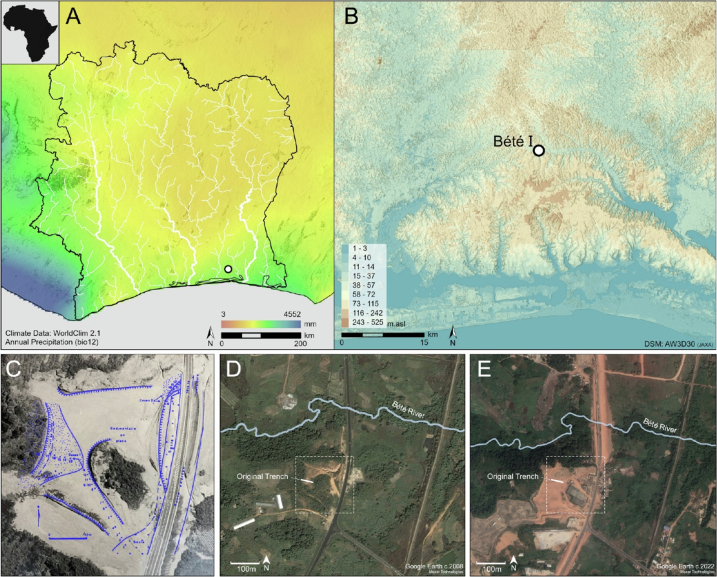
Location and details of quarry site at Anyama. **A**, Map of Côte d’Ivoire, showing modern annual precipitation^71^ and river systems. **B**, Digital surface model showing location of Bété I within the wider region. **C**, Historical aerial imagery of the Anyama Quarry with Bété I sequence (c. 1980s, courtesy of F.Y.G.) with site plan^72^ showing trench locations. **D**, Google Earth imagery from 04/2008, showing conditions at the site and the Bété River, and **E**, imagery from 01/2022, showing the expansion of the quarry and subsequent destruction of the site.

**Extended Data Fig. 2:**
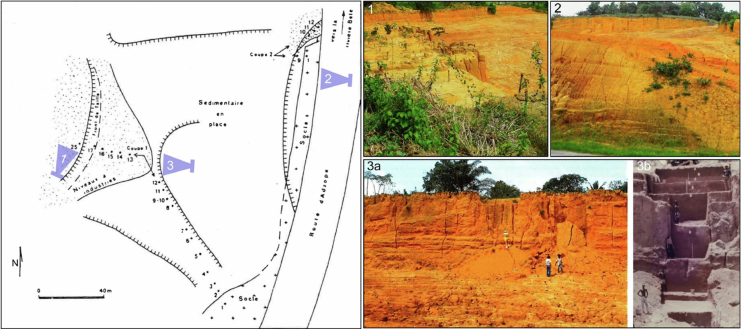
Historical photos of excavations at Bété I. See SI-1 for details. **1**–**3a**, Photos of Bété I in November 1983 taken by P. Allsworth-Jones during a visit to the site, and **3b**, photo of main trench at Bété I during excavation in 1990s (courtesy of F.Y.G.).

**Extended Data Fig. 3:**
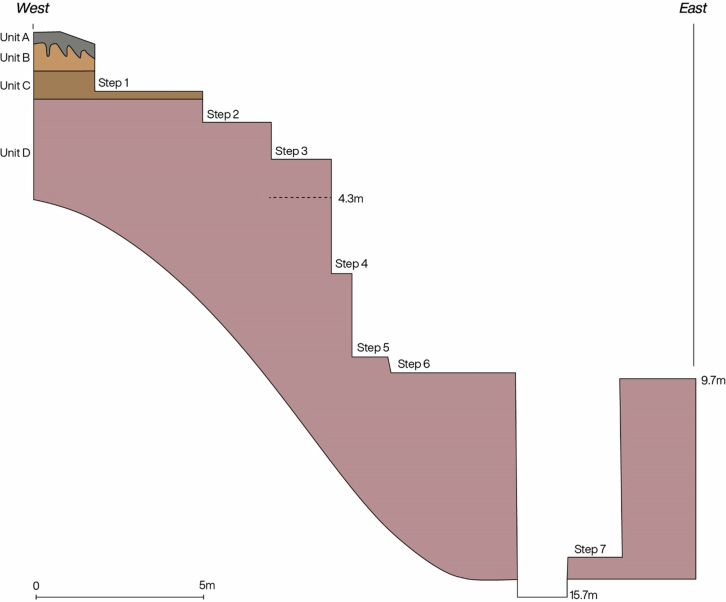
Historical longitudinal section across the Bété I excavation. Longitudinal section across the Bété I excavation area (Steps 1–3), and the cleaned Quarry wall (Steps 4–6) and Test Pit 1 (Step 7), redrawn from Figure 6 in ref. ^21^, differentiating the Terre de Barre deposits of Units A-D and highlighting the steps re-examined in this study (Steps 1–4) and the lowermost artefact bearing level at 4.3 m.

**Extended Data Fig. 4:**
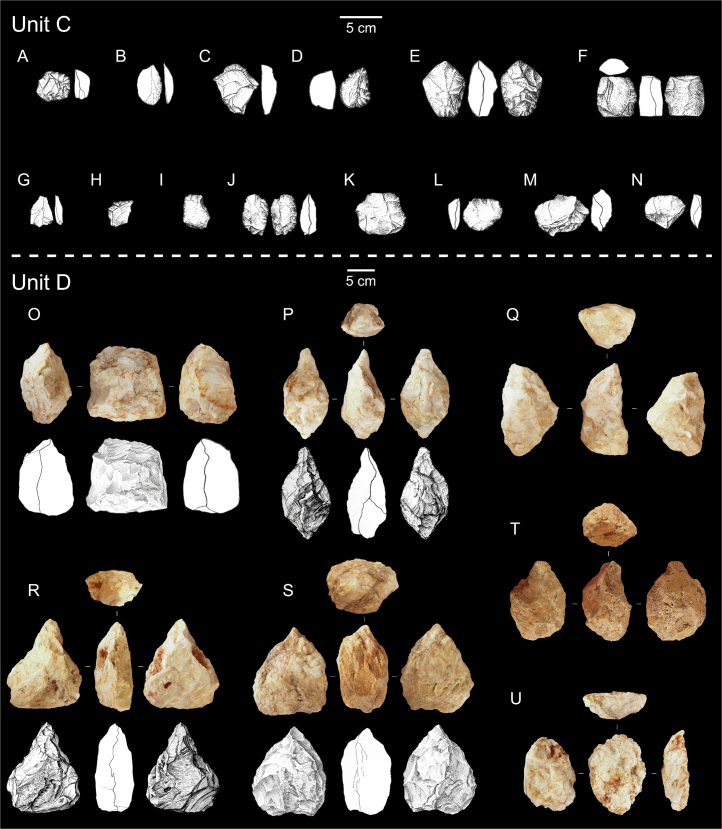
Stone tools from Units C and D at Bété I and III from ref. ^21^, and photos taken of the remaining artefact collection at the Institut des Sciences Anthropologiques de Développement (ISAD) in 2021. Unit C: **A**, ‘end-scraper’; **B**, ‘point’; **C**, ‘end-scraper à museau’; **D**, ‘double-ended carinated end-scraper’; **E**, ‘small handaxe’; **F**, ‘fragment of a bifacial foliate piece’; **G**, ‘point Levallois’; **H**, ‘combination tool’; **I**, ‘end-scraper with spine’; **J**, ‘short foliate biface’; and **K**-**N**, ‘cores’. Unit D: **O**, ‘side and end chopper’; **P**, ‘biface - trihedral’; **Q**, bifacial LCT (our term); **R**, ‘pick with double-flat cross-section of the body and centred quadrihedral distal point’; **S**, ‘pick with double-flat cross section of the body and centred trihedral distal point’; **T**–**U**, bifacial pieces (our term). **A**-**N** and **O**,**P**,**R**,**S** are from ref. ^21^.

Here we report a suite of analyses from the site of Bété I, located in the Anyama locality of Côte d’Ivoire, West Africa (Fig. 1 and Extended Data Figs. 1–4), that demonstrates a deep-time association between humans and wet tropical forests dating to around 150 ka (Marine Isotope Stage (MIS) 6) (Fig. 2). This association is both geographically and ecologically distinct from contemporaneous sites known from across Africa. The site of Bété I (5.515° N, 4.06° W) is located approximately 20 km north of Abidjan, where an extensive Quaternary sequence is displayed in several deep sedimentary exposures revealed by quarrying activity (that is, Bété I–IV), as first reported in ref. ^20^. These were resolved into six stratigraphic units (F to A, from the base to the top) and further subdivided into 14 layers (Supplementary Information Section SI-1 and Supplementary Figs. 1–3) during excavations undertaken between 1982 and 1993 by a joint Ivorian–Russian mission that focused on an approximately 14-m step trench, with deeper exposures observed across the quarry site^21^. These broadly comprise a weathering horizon of the chloritic shale bedrock (Unit F), coarser alluvial deposits attributed to the Continental Terminal (Unit E), finer, gravel-free alluvial terre de barre deposits that show extensive weathering in the lower levels (Units D and C), along with recent subsoil (Unit B) and a topsoil horizon (Unit A). A radiothermoluminescence study initially dated the Unit E deposits to the Early and Middle Pleistocene. Although these ages should be regarded with extreme caution (see Supplementary Information Section SI-1 for a critical evaluation), an estimate of 254 ± 51 ka from Unit D in sediments underlying archaeological horizons provides a tentative terminus post quem for human presence at the site^21^. Dating of the nearby valley floor deposit has indicated an Early Holocene or terminal Pleistocene incision to establish the modern drainage, probably cutting the terrace deposits (Supplementary Information Section SI-2). Key stone tool assemblages recovered from Unit D include a prominent heavy tool component, such as picks (Supplementary Information Section SI-1 and Supplementary Figs. 1 and 4), alongside small retouched tools (Supplementary Information Section SI-1 and Supplementary Fig. 4). Unit C has assemblages with Levallois reduction and small retouched tools. Unfortunately, the lithic artefact collections were lost during the 2011 civil war.

Today, this site lies within the modern distribution of wet–humid West African tropical rainforest, which encompasses a diversity of forest types, including periodically to permanently inundated swamp and riparian forests, as well as evergreen rainforest^22,23^. Because the site represents the deepest stratified site yet found in Africa’s (present-day) tropical forest regions, we returned to re-investigate it in 2020. The site was unfortunately destroyed later, between 2020 and 2021 (ref. ^24^), by quarrying activities.

We located the original step trench at Bété I, and cut back and cleaned the uppermost four steps of the original excavation, spanning the top 5.65 m of the sedimentary sequence. Our field records matched the sequence reported in ref. ^21^, comprising four discrete sedimentary units referred to as Units A–D. A suite of 37 sediment samples were recovered from this sequence for new sedimentological and palaeoecological analyses. The quantified description of the physical characteristics of the deposits matched those from the field records and previous studies, but also highlighted a discrete transition between Units C and D (Fig. 2, Supplementary Information Section SI-2 and Supplementary Fig. 5). The sedimentology supported the interpretation of a low-energy alluvial environment with episodic hiatuses presenting a high-resolution depositional setting for the archaeological assemblages (Supplementary Information Section SI-2 and Supplementary Table 1), with earlier identification of fine knapping debris at the site indicating little likelihood of post-depositional disturbance.

**Extended Data Fig. 5:**
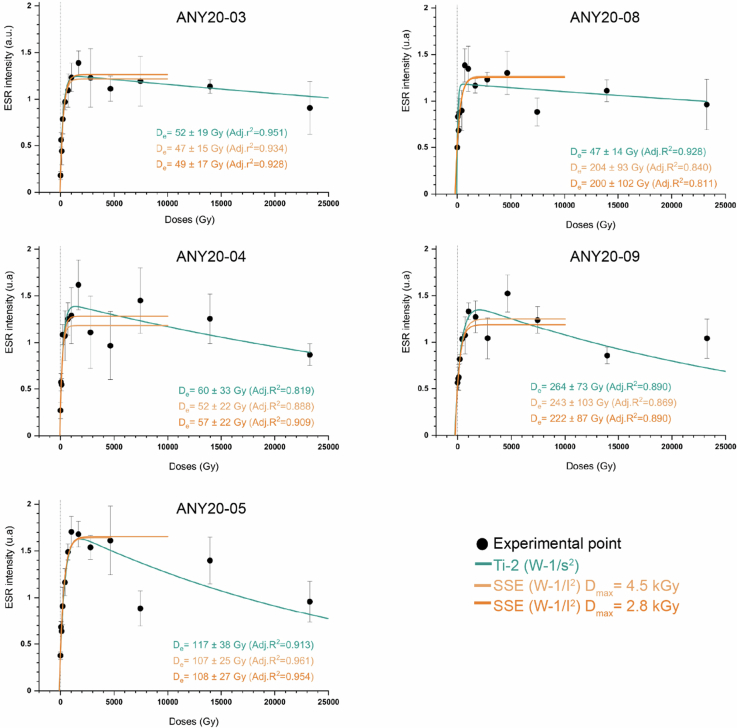
ESR dose response curves (DRCs) derived from the Ti-H ESR signal measured in the quartz samples from Bété I. Fitting was performed as follows: (see text for further information): **a**, with the Ti-2 function (data weighting by 1/s^2^); **b**, with the single saturating exponential (SSE) function (data weighting by 1/I^2^) with D_max_ = 4.5 kGy; and **c**, D_max_ = 2.8 kGy. Each experimental data point and associated vertical errors bar represent the mean ESR intensity and 1 standard deviation derived from the repeated measurements, respectively. DRCs display ESR intensities after noise subtraction.

**Extended Data Fig. 6:**
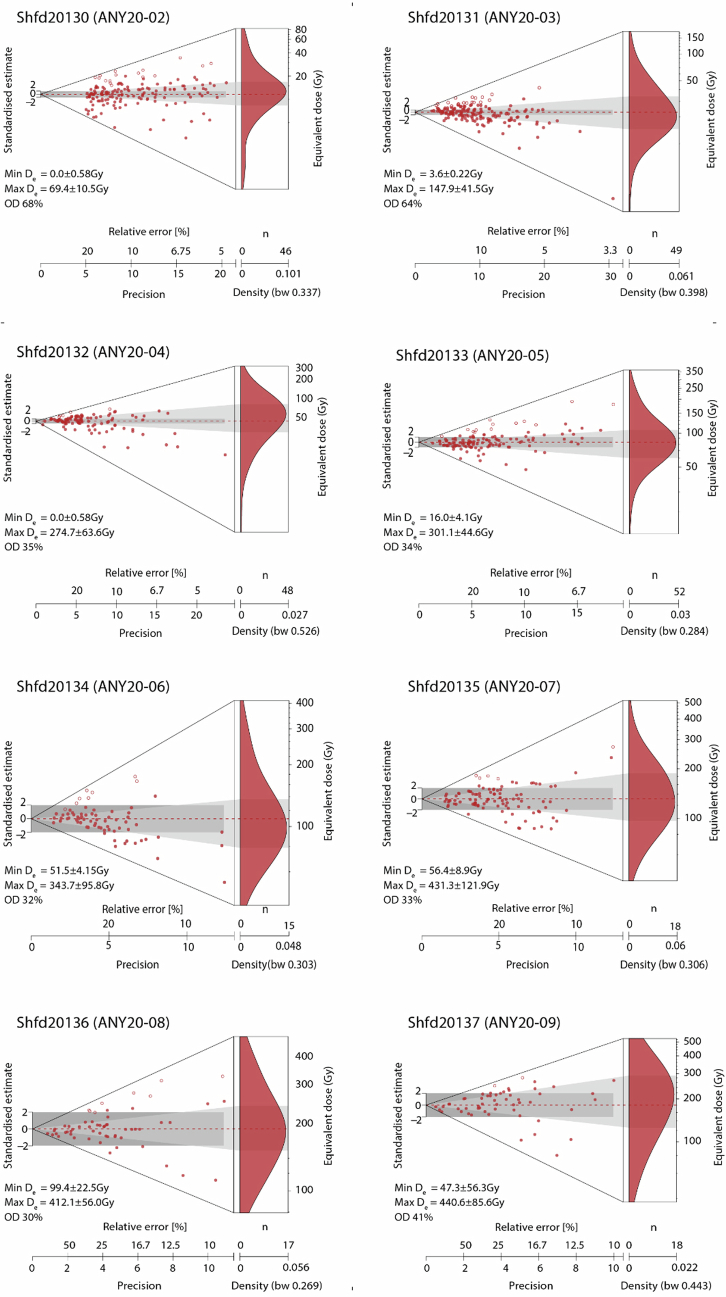
Abanico plots of D_e_ replicate single grain (SG) OSL data centred on the Central Age Model D_e_ also used for age calculation purposes. Open circles indicate aliquots considered as outliers (see SI-3 for details). Overdispersion (OD) as shown is prior to outlier removal and grey bar shows the 2 standard error estimate on the Central Age D_e_.

The chronology of the Bété I site was obtained using a combination of single-aliquot (SA) and single-grain (SG) optically stimulated luminescence (OSL) and multiple centre (MC) electron spin resonance (ESR) dating, both applied to quartz grains extracted from the sediments (Supplementary Information Section SI-3). In total, eight SA and SG-OSL and five MC-ESR ages (all reported with 1*σ* error) were calculated for various samples from Units C and D (Fig. 2 and Supplementary Information Section SI-3). The ages are, overall, stratigraphically consistent from the base to the top of the Bété I sequence, and range from the late Middle Pleistocene to the Pleistocene/Holocene transition, contributing to the establishment of a coherent age–depth model for the in situ stone tool assemblages from Units D and C. Our critical evaluation of the combined OSL-ESR dataset indicated that: (1) the SG-OSL results can be regarded as the most reliable estimates of the true burial age of the deposits; (2) the SG-OSL chronology is supported, in most samples, by the semi-independent age control provided by the SA-OSL and MC-ESR results; and (3) among the various sets of ESR ages obtained through the MC approach, those derived from the titanium centre of quartz (Ti–H) signal are regarded as providing a closer estimate to the true burial age. A complete discussion of the dating results is presented in Supplementary Information Section SI-3 (see also Supplementary Figs. 6–14 and Supplementary Tables 2–11) and Extended Data Figs. 5 and 6. At the bottom of the sequence, the SG-OSL age of the deepest sample, ANY20-09, was 166 ± 14 ka (placing it in MIS 6, 130–190 ka, ref. ^25^), providing a maximum age constraint for the deposits. The chronology of the lithic assemblages from Unit D, with the larger tool component, is bracketed by SG-OSL ages of 146 ± 9 ka (ANY20-08) and 55 ± 3 ka (ANY20-05). By comparison, the Ti–H ESR ages are older, but consistent at 1*σ*, because of their large associated uncertainties (Supplementary Information Section SI-3). In particular, sample ANY20-08 is associated with the deepest location of lithic artefacts in Unit D, and provides an age of around 150 ka (in MIS 6) for the earliest evidence of human presence at the site (Supplementary Information Section SI-3).

The ages of 35 ± 3 ka (SG-OSL) and 44 ± 19 ka (ESR) from the transition from Units D to C are relatively coherent, and agree at 1*σ*, indicating the end of Unit D deposition towards the end of MIS 3. Further up in the sequence, two SG-OSL ages from Unit C constrain the typical MSA artefacts to between 20 ± 1 ka (ANY20-03) and 12 ± 1 ka (ANY20-02), placing this unit in MIS 2.

**Extended Data Fig. 7:**
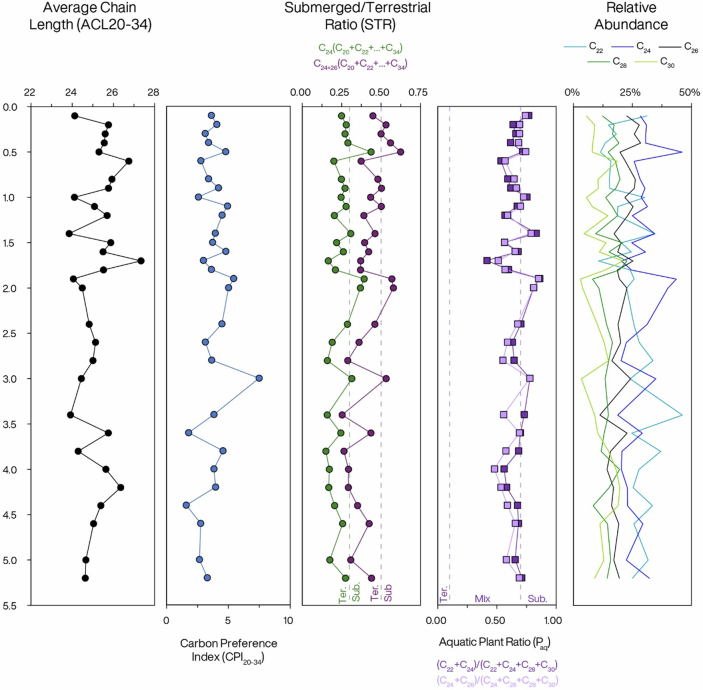
Biomarker signatures of the Bété I archaeological sediments. See Section 4.2.1 for ACL and CPI calculations. Two versions of the submerged/terrestrial ratio were calculated: STR24 and STR24 + 26. Submerged aquatic plants tend to have values greater than 0.25 and 0.50 for STR24 and STR24 + 26, respectively. Two versions of the aquatic plant ratio (P_a__q_) were also calculated to compare with STR. Terrestrial plants typically have sedimentary P_aq_ values of less than 0.1 while aquatic plants tend to have values greater than 0.7. Values ranging between 0.1 and 0.7 typically represent a mixed input that includes terrestrial, emergent, and aquatic plants. The percentages of compound abundances are relative to the dominant FAMEs (C22 + C24 + C26 + C28 + C30). Higher values reflect relatively increased inputs of the respective compounds.

The delta 13 carbon (δ^13^C) measurements of the bulk soil organic matter (SOM) from the sediments taken through the Bété I sequence are shown in Fig. 2, Supplementary Table 10 and Extended Data Fig. 7. The values range from −25.4 to −27.6‰ (−26.6 ± 0.6‰, *n* = 35) and overlap with the bulk δ^13^C values measured from contemporary African rainforest contexts^26^, corrected for the Suess effect^27–30^, of −24.8 to −34.5‰ (−28.1 ± 1.9‰, *n* = 24). The SOM δ^13^C is usually assumed to represent standing plant biomass and plant remains introduced by either humans (for example, carried for bedding or other uses) or nature (for example, from wind and water transport)^31^, with an increase of +1 to +3 per mil in SOM δ^13^C values relative to the contributing plant matter being the result of microbial activity^32^. With this in mind, the Bété I bulk δ^13^C values primarily indicate C3 biomass, with an increase in values recorded from sample 31 (Unit D3) to sample 29 (Unit D2), from 4.2 to 3.8 m deep, and δ^13^C fluctuations from sample 21 (Unit D1, at 2.2 m deep) upwards with a steady trend towards increasing values towards the top of the sequence (Unit A). To discern the exact drivers of these trends, we combined this isotopic analysis with biomarker analyses (leaf-wax) and the examination of phytoliths and pollen from the sequence.

Of the 37 palaeoenvironmental samples, 31 had sufficient lipid material for plant wax biomarker analysis through gas chromatography mass spectrometry (GCMS). Even-numbered, mid-chain-length (C_22_–C_24_) *n*-alkanoic acids (as fatty acid methyl esters (FAMEs)) dominated the biomarker distributions, indicating high input from submerged or emergent plant wax sources for most samples^33,34^. The average chain length (ACL_20–34_) ranged from 23.9 to 27.3 (25.2 ± 0.84, *n* = 31) (Supplementary Table 13), which is typically lower than previous reports of modern ACLs from African terrestrial plants^35–39^. The aquatic plant ratio (*P*_aq_ C_22+24_) ranged from 0.41 to 0.86 (0.66 ± 0.09, *n* = 31), and the submerged/terrestrial ratio (STR) of the C_24_ FAME (STR_24_) ranged from 0.15 to 0.44 (0.25 ± 0.07, *n* = 31) (Fig. 2 and Supplementary Table 13). Both proxies indicate that abundant wetland-adapted species, either fully submerged or emergent plants, were principal contributors of plant wax biomarkers to the site (Supplementary Information Section SI-4 and Supplementary Figs. 15 and 16). The STR values, however, indicate that terrestrial plants also provided plant wax biomarkers to the Bété I sediments. There also seems to be a changing pattern in the relationship between the abundance of the C_24_ FAME and the bulk sedimentary δ^13^C. For instance, below 2.0 m (from the lower section of Unit D3 to the middle of Unit D1), the δ^13^C co-varies in the same direction as both the STR and *P*_aq_ (Spearman’s correlation STR_24_ *r*_s_ = 0.248, *P* = 0.394; *P*_aq_ *r*_s_ = 0.597, *P* = 0.024). However, above 2.0 m (from the middle of Unit D1 to the middle of Unit A), there is an anti-phased correlation between the C_24_ FAME and the bulk sedimentary δ^13^C (Spearman’s correlation *r*_s_ = −0.377, *P* = 0.136). That is, as the abundance of C_24_ increases relative to the other FAMEs, the δ^13^C decreases. Additionally, as both the STR and *P*_aq_ values increase, indicating greater input from submerged/aquatic plant biomarkers, the bulk sedimentary δ^13^C shifts lower (Spearman’s correlation STR_24_ *r*_s_ = −0.441, *P* = 0.077; *P*_aq_ *r*_s_ = −0.240, *P* = 0.353). It is possible that, as local forest characteristics changed, such as with forest succession, the bulk isotope signal changed in accordance with the abundance of terrestrial or aquatic plants.

Nine samples were analysed for phytoliths and pollen to establish the preservation and representation of microbotanical evidence, selected to match peak artefact densities, correlate to human habitation of the site, and provide further insights into the trends observed in the biochemical data. The deposition and preservation of these two types of plant microfossil is inversely correlated, such that samples with a low phytolith influx (Supplementary Information Section SI-4 and Supplementary Fig. 17) tend to have a higher influx of pollen (Supplementary Information Section SI-4 and Supplementary Tables 15 and 16), but every sample yielded plant microfossils.

**Extended Data Fig. 8:**
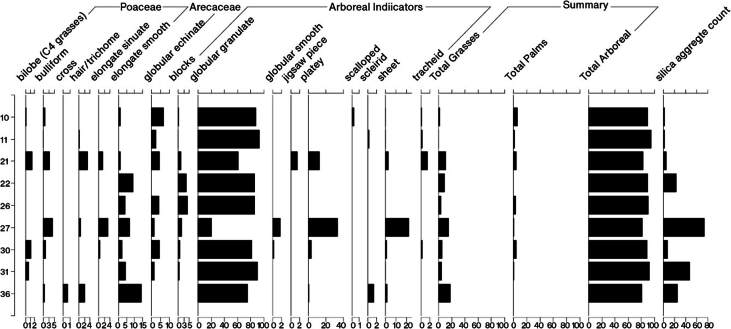
C2 diagram of percentages of phytoliths types. Samples 21, 22, 26, 27 and 36 are calculated on phytolith counts below the statistically viable minimum number of recorded phytoliths so their interpretation should be treated with caution.

The phytolith assemblages produced by nine samples (samples 10, 11, 21, 22, 26, 27, 30, 31 and 36) (Supplementary Table 14) established their preservation. Morphotype identification demonstrated that all samples were dominated by arboreal phytolith morphotypes (Extended Data Fig. 8), with 82–96% of these morphotypes representing trees/shrubs, and therefore being indicative of a C3-dominated biomass in all cases (Supplementary Fig. 18). All of the arboreal phytolith types identified in the nine sediment samples are produced in most dicotyledons, so they could not be attributed to the genus or species level. Arecaceae phytoliths (palms) made up between 2 and 7% of the identified assemblages in seven of the nine samples, reaching a maximum in the uppermost sample (sample 10). Grass phytoliths made up between 1 and 18% of the assemblage, with the highest values coming from the lowermost sample (sample 36). Grasses peak again in sample 27 (16%) and fell to their lowest percentages in the uppermost unit (sample 10, 3%, and sample 11, 1%). It was not possible to directly compare the number of phytoliths produced by monocots and dicots because plants have different abilities to produce phytoliths—in particular, the Poaceae produce more than other monocotyledons^40^. As monocotyledons can produce up to 20 times more phytoliths than the dicots^41^, the dominance of arboreal phytoliths in all samples (more than 81%) is a clear signal of local forest cover with relatively few grasses or palms.

**Fig. 3:**
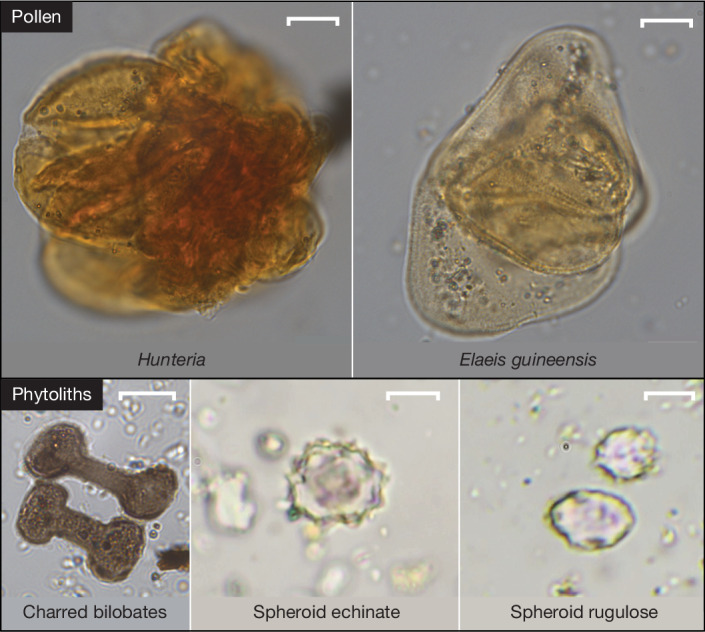
Key pollen and phytolith taxa found at Bété I. Examples of anther fragments (sample 30) from pollen taxa typical of rainforest (*Hunteria*) and flooded forest (*E. guineensis*) and phytoliths (respectively from samples 20, 30 and 22) preserved in Unit D of the Bété I sequence. Scale bar, 10 µm.

**Extended Data Fig. 9:**
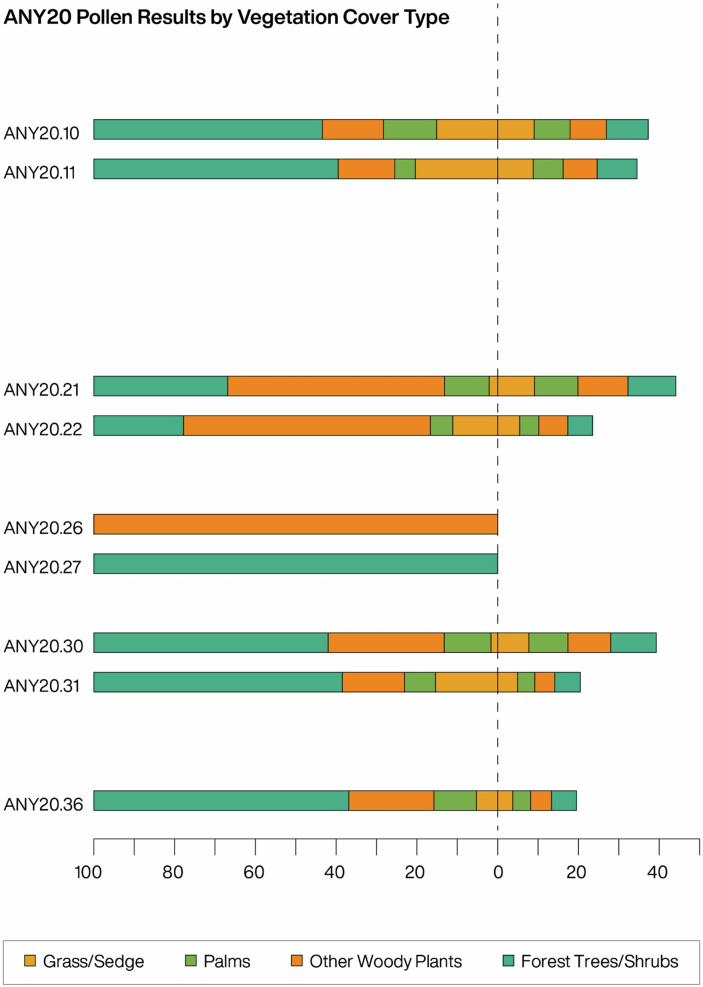
Barplot showing proportional representation of key groups of pollen (left) and log values of pollen concentrations (right). Note that samples 26 and 27 do not have sufficient pollen concentrations to produce meaningful proportions.

The pollen assemblages (Supplementary Figs. 19–26, Supplementary Tables 16 and 17 and Extended Data Fig. 9) in these samples were dominated by dicot pollen types (70–80%), followed by grasses (10–20%) and palms (5–10%). The pollen types were attributable to species, in some cases (*Elaeis guineensis* Jacq.), and to the genus (*Hunteria*) or family (Poaceae) level in others. There was a consistent presence of pollen types typical of wet–humid West African rainforests, riparian forests and swamp forests. Early riparian forest succession is signalled by the co-occurrence of *E. guineensis* (oil palm), dense shrubs/trees belonging to the genera *Alchornea* and *Macaranga*, and gap-colonizing trees, such as *Anthocleista* (Supplementary Fig. 22 and Supplementary Table 17). *Canarium schweinfurthii* and *Pentadesma* are both large trees frequently found in the later stages of forest succession in seasonally inundated conditions near rivers and lakes. These pollen types were more common in Unit D (specifically in the middle of D2 and the top of D3) and were absent from Unit C, being replaced by *Uapaca*, which is a diverse genus with many riparian-affiliated species. Unit C also yielded taxa typical of riparian forests (*Parinari* sp.), as well as types widespread in the Guineo–Congolian forest zone (*Rauvolfia* sp.). The Unit D samples contained more *Hunteria* pollen, probably attributable to *Hunteria umbellata*, which is common in wet–moist West African tropical rainforests^23^, particularly in forests adjacent to waterways^42^. The presence of anther fragments from *E. guineensis* and *Hunteria* (Fig. 3) in sample 30 from the top of Unit D3 presents strong evidence for whole flowers/anthers falling directly from the plant and being incorporated into the sedimentary matrix, supporting the local rainforest signal.

**Fig. 4:**
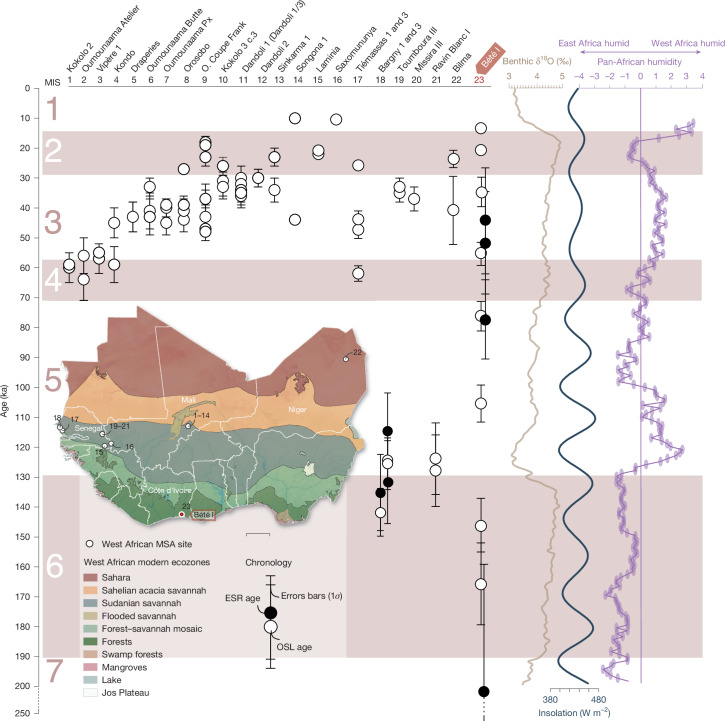
West African dated Stone Age sites with key off-site palaeoenvironmental proxy records. Insolation curve (dark blue) from ref. ^46^. Inter-regional African humidity curve (purple) from ref. ^47^. Benthic curve (light brown) built from ref. ^48^. Synthesis of dated occupations from West Africa from refs. ^48–50^ (for more details, see Supplementary Information Section SI-5). The Later Stone Age sites are not included. Error bars associated with OSL and ESR ages represent ± 1*σ* uncertainties.

The SG-OSL dates of around 20–12 ka for the MSA assemblage in Unit C and 150–55 ka for the assemblages differentiated by a large tool component in Unit D document persistent human occupation of the Anyama area after the Middle Pleistocene. However, it is the lithic assemblages in Unit D that are of particular interest, as they are associated with a wet and forested environment. The palaeoenvironmental proxies from Unit D show no evidence for open and dry grassland, sparse savannah, or wooded savanna vegetation cover, despite the tendency for grasses to be over-represented in microfossil records. The sedimentary, biomarker and microfossil results are remarkably consistent, showing evidence for alluvial deposition in a tropical forest environment and consisting of riparian, swamp and rainforest taxa. Currently, the assemblages of Bété I from Unit D dating from MIS 6 are the oldest found outside the sahelian and sudanian savannah biomes of West Africa (Fig. 4). On a broader scale, the Unit D lithic assemblages are contemporaneous with other MIS 6 MSA stone tool assemblages found in other African sites located in different ecoregions, such as savannahs, or close to modern coasts (*n* = 16) (Fig. 1 and Supplementary Table 18 in Supplementary Information Section SI-5 and references therein) in West Africa (Bargny 1 and Bargny 3), in northern Africa (Bizmoune, Ifri n’Ammar, Wadi Lazalim (site 16/29), Bîr Tirfawi, Taramsa 1), in eastern Africa (Sai Island 8-B-11, EDAR 135, Abdur) and in South Africa (Amanzi Springs, Florisbad, Pinnacle Point 13B, Wonderwerk, Border Cave, Bundu Farm).

Several independent lines of evidence have confirmed the association between humans and tropical wet broadleaf forest at Anyama, starting at least 150 ka. The consistent forest signal over time also indicates that this area of West Africa possibly acted as a rainforest refugium during arid periods (Supplementary Information Section SI-4). This corroborates projections of Middle and Late Pleistocene vegetation showing reduced, but persistent, rainforest cover at lower latitudes^43^. These data confirm a deep-time connection between human evolution and wet tropical forests, and highlight the importance of Africa’s many biomes and diverse ecoregions in this process^9,44^. The assemblages from Unit C, featuring Levallois flakes and points, side and end retouched pieces, add to the emerging evidence of a chronologically persistent MSA in West Africa towards the end of the Late Pleistocene that is probably a key regional characteristic^7,45^.

The assemblage in Unit D, featuring large tools alongside a small tool component, may support long-held views that the diverse heavy-duty tool assemblages seen in Central and West Africa are convergent adaptive solutions to tropical forest habitation^6,16^. The combination of an MIS 6 age with the ecological context of the Unit D assemblage is without precedent elsewhere in Africa. As a result, the archaeological classification of the Unit D assemblage warrants circumspection and further study (Supplementary Information Section SI-1). This is particularly the case because West Africa remains under-researched compared to other regions of the continent, and its archaeological sequence and specific regional characteristics are yet to be fully understood (Fig. 2 and Supplementary Information Section SI-1). Most importantly, however, our results confirm a deep-time connection between human evolution and tropical forest biomes, opening up a new chapter in the human past in which our species occupied dense, wet tropical forests much earlier than widely thought. This association confirms the predictions of the pan-African model of human evolution, and highlights the importance of Africa’s many regions and ecosystems in this process^9^.

## Methods

### Geochronology

Owing to the absence of organic/bone remains or minerals from volcanic deposits, the use of dating methods such as uranium (U) series, ESR combined with uranium-series, or argon/argon was not possible. The chronology of the Bété I site was therefore constrained using a combination of OSL and ESR dating methods, both applied to optically bleached quartz grains through a range of techniques and measurement procedures (single/multiple aliquots (SA/MA), single/multiple grains (SG/MG), multiple centre (MC) approach). Eight samples were dated along the sequence using SA-OSL and SG-OSL, whereas three replicate ESR ages (aluminium (Al), Ti–H and Ti–mix signals) were calculated for five of these samples.

#### Luminescence dating and palaeodose evaluation

Eight sediment samples (Supplementary Table 2) from the Bété I excavated sequence were collected using opaque metal tubes (25 cm in length and 4 cm in diameter). Three sediments were sampled from Unit C, one at the interface between Units C and D and five from Unit D. The samples were taken from sections extended following the first excavations of the site. The samples cover the uppermost 5 m of the stratigraphic sequence. Sample preparation was carried out at the Sheffield Luminescence Dating Laboratory (University of Sheffield, UK) (for the detailed preparation procedure and analysis of the samples, see Supplementary Information Section SI-3). The OSL measurements were undertaken both at the small aliquot (2 mm diameter; SA) and SG levels. The OSL dose reconstruction using the SA regenerative-dose (SAR) protocol^51^ involved a single saturating exponential fit for younger samples and an exponential and linear fit for older samples. Samples were accepted for further analysis based on the following criteria: (1) the OSL signal measured 3*σ* above background; (2) the SAR growth curve passed within 1*σ* errors of the regenerative dose points; (3) the recycling values were within ±10% of unity for SA and ±20% for SG; and (4) the error on the test dose used in the SAR protocol was less than 10% for SA and 20% for SG. Equivalent doses (*D*_e_) were determined using the central age model^52^, minimum age model^52^ and finite-mixture model^53,54^. The final selected *D*_e_ for each sample was divided by the dose rate (Supplementary Information Section SI-3 and the ‘Dose rate evaluation’ section) to derive the burial ages.

#### ESR dating and dose evaluation

One sediment sample from Unit C (ANY20-03), one from the interface between Units C and D1 (ANY20-04) and three from Units D1 and D3 (ANY20-09, ANY20-08 and ANY20-05) delivered sufficient quartz material for ESR dating. The MC approach^55^ was employed, while the standard multiple aliquot additive dose method was used for the dose determination. Each quartz sample was divided into 14 aliquots—one natural (non-irradiated), one ultraviolet-bleached and 12 laboratory gamma-irradiated aliquots, irradiated up to about 23 kGy. The Al and Ti signals were repeatedly measured in each sample, three to four times over different days, and were corrected by their corresponding receiver gain value, temperature factor^56^, number of scans and aliquot mass. The noise was extracted and subtracted from the Ti ESR intensities following ref. ^57^. Dose response curves for each signal were obtained from the mean ESR intensity values and associated 1 s.d. derived from the repeated measurements. The experimental points were fitted using Microcal OriginPro (OriginLab Corporation) using a Levenberg–Marquardt algorithm by chi-square minimization, and several fitting functions were tested for *D*_e_ determination (Supplementary Information Section SI-3). The final *D*_e_ uncertainties included a dose rate calibration error (1*σ*) of 2.3%. The detailed analytical procedure can be found in Supplementary Information Section SI-3.

#### Dose rate evaluation

For the ESR and OSL dating analyses, the total dose rate was derived from laboratory measurements. For each dated sample, approximately 3–5 g of dry milled sediment were analysed by inductively coupled plasma mass spectrometry to quantify the potassium (K), thorium (Th), U and rubidium (Rb) concentrations. These concentration values were used to calculate the alpha, beta and gamma dose rate components using conversion factors from ref. ^58^. In addition, approximately 30 g of this same raw sediment, previously dried and powdered, were analysed by high-resolution gamma spectrometry using a high-purity germanium detector to identify possible disequilibrium in the U^238^ decay chain. Dose rate values were calculated for a grain-size fraction of 180–212 µm (nominal sieve opening sizes) and an assumed thickness removed by hydrofluoric acid etching of 10 ± 5 µm (ref. ^59^). The values were corrected using beta and alpha attenuation values for spherical grains^60,61^. An internal dose rate of 30 ± 10 μGy per annum was considered, as commonly used in OSL and ESR dating application studies^62^. An alpha efficiency of 0.07 ± 0.01 (ref. ^63^) was used. The ESR and OSL dose rates and ages were calculated using the dose-rate and age calculator from ref. ^64^ (v.1.2) and the errors were 1*σ*. For both datasets, we used the same cosmic dose-rate values using the formula from ref. ^65^, with depth, altitude and latitude corrections applied^66^. Palaeomoisture contents were applied based on estimations of the average long-term burial water contents, with an associated absolute uncertainty of ±5% (see Supplementary Information Section SI-3.4 for further details).

### Sedimentology and palaeoecology

Thirty-seven sediment samples were collected from along the sedimentary sequence (Fig. 2) for sedimentology, stable isotope, plant wax biomarker, phytolith and pollen analyses.

#### Sedimentology

For laser particle-size analysis, sieved sediment samples (approximately 1 g, less than 2 mm) were bathed in sodium hexametaphosphate solution (0.5%) for 24 h and then agitated in an ultrasonic bath, with the samples rinsed in purified water before analysis in a Malvern Mastersizer 3000. Characterization of the grain-size results was conducted using GRADISTAT software^67^. For loss-on-ignition analysis, sediment samples (approximately 10 g) were weighed (to three decimal places—0.001 g) and heated in a muffle furnace to 105 °C, 550 °C and 950 °C (allowing the samples to cool to 105 °C for weighing between steps) to calculate the proportions of water, total organic matter, carbonates and mineral residue. Magnetic susceptibility was measured in the laboratory using a Bartington MS3 magnetic susceptibility meter coupled with an MS3B sensor to analyse approximately 12-g samples, weighed on precision scales, to allow calculation of the mass specific values, presented as *Χ*_mass_ (10^−8^ m^3^ kg^−1^).

#### Stable isotope

The preparation of the bulk organic matter stable isotope samples, and their associated analysis, was as follows. Bulk sediment samples were weighed and sieved through a 2-mm mesh to remove stones and large macrobotanical materials. Then, 2 M hydrochloric acid (HCl) was added to 1 g of each sieved sample to remove any carbonates before the samples were rinsed three times using deionized water, interspersed with centrifugation. The remaining residues were then freeze-dried for 48 h. Duplicates of 1-mg aliquots of dry sample were weighed out into tin capsules. The δ^13^C measurements of the samples were measured using a Thermo Scientific Flash 2000 elemental analyser coupled with a Thermo Delta V Advantage mass spectrometer at the Isotope Laboratory, MPI-GEA, Jena. The isotopic values are reported as the ratio of the heavier isotope to the lighter isotope (^13^C/^12^C) as δ values in parts per mil relative to the Vienna Pee Dee Belemnite (VPDB) standard. The results were calibrated against international standards—IAEA-CH-6 (δ^13^C = −10.49 ± 0.47‰) and USGS40 (δ^13^C = 26.38 ± 0.042‰). The international standard USGS61 (δ^13^C = −35.05‰) was used to check machine precision. On the basis of replicate analyses, the long-term machine error over a year was ±0.2‰ for δ^13^C. The overall measurement precision was recorded throughout.

#### Plant wax biomarker analysis

Dry, homogenized sediments (approximately 20 g) were extracted using a Büchi SpeedExtractor E-916 pressurized speed extractor and 9:1 (v/v) dichloromethane to methanol at 100 °C and 103 bar (1,500 psi) in three, 10-min cycles. The total lipid extract (TLE) was separated into neutral, acid and polar fractions by aminopropyl column chromatography. The acid fraction was methylated to produce FAMEs and isolated using silica gel column chromatography. The FAMEs were then analysed using an Agilent 7890B gas chromatography (GC) system equipped with an Agilent HP-5 capillary column (30 m long, 0.25 mm internal diameter and a 0.25-μm film) coupled to a 5977 A Series mass selective detector (MSD) at the Max Planck Institute of Geoanthropology (Jena, Germany). The FAMEs were identified by comparing their mass spectra and retention times against an external standard mixture. See Supplementary Information Section SI-4 for further details on the biomarker molecular characterizations.

#### Phytoliths

The phytolith samples were processed using a standard phytolith extraction protocol as follows: (1) the sample was screened through a 0.5-mm mesh to remove coarse-sized particles; (2) approximately 2 g of dried raw sediment was weighed out; (3) the calcium carbonates were dissolved using a dilution of 10% HCl, the samples then being washed in distilled water three times; (4) the clay was removed using a settling procedure and sodium hexametaphosphate (Calgon) as a dispersant. Distilled water was added and the samples were left for 75 min before the suspension was poured off. This was repeated at hourly intervals until the samples were clear. The samples were then transferred into crucibles and left to dry at a temperature of less than 50 °C; (5) after drying, the samples were placed in a muffle furnace for 2 h at 500 °C to remove the organic matter; (6) the phytoliths were then separated from the remaining material using a heavy liquid calibrated to a specific gravity of 2.3. The phytoliths were transferred to centrifuge tubes and washed three times in distilled water. They were then placed in small Pyrex beakers and left to dry; (7) approximately 2 mg of phytoliths per sample were mounted onto microscope slides, using the mounting medium Entellan. The microscope slides were assessed using a Meiji MT4300L transmitted light microscope using ×100 and ×400 magnifications. The phytoliths were counted and categorized into types, and further classified as deriving from either woody (dicotyledon) or non-woody (monocotyledon) taxa.

The weight percentage of the phytoliths produced by each sediment sample was calculated by dividing the weight of the phytoliths extracted from the sample by the original sample weight multiplied by 100 (Supplementary Fig. 11). This number is an approximation because other siliceous materials may also have been extracted at the same time (for example, diatoms and sponge spicules), and some residual material is likely to remain in the extracted samples.

#### Pollen

The samples for pollen analysis (Supplementary Table 11) were shipped to the IASCE Paleoecology Laboratory at the University of South Florida, Tampa. The samples were weighed and their volumes were estimated using displacement. Two tablets of *Lycopodium* sp. spores were added to each sample (batch no. 100320201) and dissolved with 10% HCl. The samples were centrifuged–decanted–rinsed until pH neutral, and then placed on a shaker overnight in Calgon solution (10% sodium hexametaphosphate). The fine materials were isolated using gravity separation and screening through 250-µm sieves. Degraded and other complex organics were digested in a 10-min hot bath at 80 °C in a 10% potassium hydroxide solution. The samples were then centrifuged, the solution decanted, and a small (approximately 0.5 ml) volume of concentrated (27%) HCl was added to the samples. The samples were centrifuged–decanted–rinsed again until their pH became neutral and the supernatant was clean. The samples were rinsed in 99% glacial acetic acid, centrifuged and decanted, and then were treated using the acetolysis reaction series. Acetolysis was triggered by the addition of a 9:1 solution of acetic anhydride and sulfuric acid, each at stock concentrations (99.5% and 48%, respectively). The samples were left in a 90 °C hot bath for 6 min, centrifuged and decanted, rinsed in glacial acetic acid, and then centrifuged–decanted–rinsed until pH neutral. Microbotanical fossils were recovered from the remaining residue using density separation in a solution of 5% HCl and zinc bromide at a specific gravity of 2.3 g ml^−1^. At this density, we were able to retrieve both pollen and phytoliths from the samples. This material was retrieved from density separation in ethanol using a pipette, and then transferred into glycerin and stored in dram vials.

The samples were mounted onto glass slides in glycerin and were fixed under a coverslip using fingernail polish. Analysis was conducted using a binocular light microscope to count the number of *Lycopodium* tracer spores, native fern spores and pollen encountered during vertical transects across the microscope slide at ×400 magnification. The pollen and spores were photographed and identified at ×400 and ×1,000 magnification using published reference material^68–70^, online reference material (African Pollen Database) and unpublished digitized reference material collected from Göthe Universität (Frankfurt, Germany), the Muséum national d’Histoire naturelle (MNHN, Paris, France) and Centre de Recherche et d’Enseignement en Géosciences de l’Environnement (CEREGE Arbois, Aix-en-Provence, France). The tracers, spores and pollen were tallied until either a total of 200 pollen grains or 100 *Lycopodium* spores were encountered.

### Reporting summary

Further information on research design is available in the Nature Portfolio Reporting Summary linked to this article.

## Online content

Any methods, additional references, Nature Portfolio reporting summaries, source data, extended data, supplementary information, acknowledgements, peer review information; details of author contributions and competing interests; and statements of data and code availability are available at 10.1038/s41586-025-08613-y.

## Supplementary information

Supplementary Information This file contains supporting information on the site background (Section SI-1), sedimentological (Section SI-2), geochronological (Section SI-3) and palaeoecological (Section SI-4) analyses, and chronological comparisons to other African sites (Section SI-5), and includes Supplementary Figs. 1–26, Tables 1–18 and references.

Reporting Summary

## Data Availability

All relevant data are provided with the paper and its Supplementary Information. Figures were created using Adobe Illustrator v.25.2.3, ArcMap v.10.5 and GIMP v.2.10.38.
