# Supplementary material for: Humans in Africa’s wet tropical forests 150 thousand years ago
Source: Nature. 2025 Feb 26;640(8058):402–7. doi: 10.1038/s41586-025-08613-y (PMC11981921; doi:10.1038/s41586-025-08613-y)
Supplement: Supplementary file 1 — This file contains supporting information on the site background (Section SI-1), sedimentological (Section SI-2), geochronological (Section SI-3) and palaeoecological (Section SI-4) analyses, and chronological comparisons to other African sites (Section SI-5), and includes Supplementary Figs. 1–26, Tables 1–18 and references. [file 41586_2025_8613_MOESM1_ESM.docx]

**Humans in Africa’s wet tropical forests**

**150 thousand years ago**

**Supporting Information**

[SI 1 - Background Information](#_Toc178429920)

[1.2 Pleistocene stone tool assemblages from African tropical forests](#_Toc178429921)

[1.3 Previous excavations and work at Bété I](#_Toc178429922)

[1.4 Stone tool technology at Anyama](#_Toc178429923)

[SI 2 – Sedimentology](#_Toc178429924)

[SI 3 - OSL and ESR dating of quartz grains](#_Toc178429925)

[3.1 Material](#_Toc178429926)

[3.2 Luminescence analyses](#_Toc178429927)

[3.3 ESR analyses](#_Toc178429928)

[3.4 Dose rate evaluation](#_Toc178429929)

[3.5. Extended discussion of the chronological results](#_Toc178429930)

[3.5.2. Ti ESR D_e_ evaluation](#_Toc178429931)

[3.5.3. Comparison of the D_e_](#_Toc178429932)

[3.5.4. Dose rate evaluation](#_Toc178429933)

[3.5.5 OSL equivalent dose evaluation](#_Toc178429934)

[3.5.7. Sensitivity test with the variation of water content](#_Toc178429935)

[SI 4 - Palaeoecological Proxies](#_Toc178429936)

[4.1 Bulk Isotope Analysis](#_Toc178429937)

[4.2 Plant Wax Biomarker Analysis](#_Toc178429938)

[4.2.1. Molecular Characterization](#_Toc178429939)

[4.2.2 Extended Results](#_Toc178429940)

[4.2. Phytolith analyses](#_Toc178429941)

[4.3. Pollen Analyses](#_Toc178429942)

[4.3.1 Laboratory Methods](#_Toc178429943)

[4.3.2 Analytical Methods](#_Toc178429944)

[4.3.3. Palynological Results](#_Toc178429945)

[4.3.4 Discussion](#_Toc178429946)

[4.3.5 Conclusions](#_Toc178429947)

[SI 5 – Chronological comparison with others African sites](#_Toc178429948)

[References](#_Toc178429949)

# SI 1 - Background Information

***1.1 Evidence for the earliest habitation of Africa’s tropical forests***

Our species, *Homo sapiens*, is thought to have emerged shortly before 300 thousand years ago (or ka) in Africa^1–3^. However multiple regions of Africa are implicated in this process^3–6^. Middle Stone age (MSA) tools associated with the earliest fossils of our species^1,2^ are found in many different regions of Africa from this stage onwards (e.g.^7,8^). Yet, the lack of chronological constraints or clear palaeoenvironmental proxy records from many sites means it has been difficult to determine the extent of ecological diversity experienced by the earliest members of our species. This is particularly the case in areas of modern-day closed-canopy forest. Long considered a barrier to Pleistocene humans (see debates e.g. in ^9–16^), the possibility that early populations of African hunter-gatherers adapted to dense tropical forests has been resurrected by discoveries in South and Southeast Asia. For example, specialized monkey-hunting^17^ and rainforest resource reliance^10,18^ in Sri Lankan rainforests has now been documented at ~45 ka, alongside the production of specialist microlithic tools argued to be adapted to hunting in dense tropical rainforest. Humans arrived in Southeast Asia between 73 and 63 ka based on finds in Sumatra^19^ and by 50-45 ka there is evidence for them practicing a mixed hunting strategy in the varied tropical environments around the Niah Caves, Borneo^20^. Similar evidence for rapid use of tropical forest environments by our species also exists in Oceania^21^ and South America^22^.

These early ages for rainforest habitation and use elsewhere raise the question as to why there is no clear evidence for the early human exploitation of tropical forests in Africa, where our species first emerged. Given growing evidence for a unique ecological plasticity defining our species, in Africa and beyond^23^, it seems more likely that evidence for ancient rainforest habitation in Africa remains simply undiscovered. Currently, the earliest secure evidence for rainforest exploitation in Africa comes from sites in the Ituri rainforest, dating to around 18 ka^11,23^, with younger dates of 12-13 ka further west at the site of Iho Eleru in Nigeria^24,25^. It has also been suggested that Later Stone Age (LSA) making humans may have been living in forested environments in Africa as early as 40 ka, but issues surrounding stratigraphy and chronology complicate these inferences^26^. At the site of Panga Ya Saidi in Kenya, there is evidence that MSA humans exploited a diverse mix of environments which included the hunting of monkeys in tropical forests ^27,28^. However, clear pre-LSA evidence for dedicated reliance on tropical forest resources, and permanent occupation, akin to that seen in Asia, for example, is currently remains lacking^10,13^.

Notably, past African rainforest fragmentation means that it is not possible to ascertain a clear association between the MSA populations and past tropical forest habitation without palaeoecological proxies and robust chronologies^29–31^. Nevertheless, an array of diverse MSA assemblages featuring large cutting tools (LCTs) have been found across Equatorial Africa, and argued to relate to the presence of dense forests in this region^11^.

## 1.2 Pleistocene stone tool assemblages from African tropical forests

Discussions on Pleistocene stone tool assemblages from African tropical forests have often centred on two stone tool industries, the so-called Sangoan and Lupemban technocomplexes, thought to represent an adaptation to closed woodland environments due to their distribution and large cutting tool component^32,33^. The Sangoan, first described from surface collections at the type site of Sango Bay in Uganda^34,35^, refers to stone tool industries that occur at the interface of the Earlier Stone Age (ESA) to MSA in sub-Saharan Africa. Assemblages attributed to the Sangoan have been variously described as late Acheulian adaptations, transitional between the Acheulean and the MSA, or as belonging to the early MSA. They are generally characterised as featuring ‘rugged’ or ‘heavy-duty’ core tools, dominated by thick bifaces, picks, choppers, and core scrapers, referred to collectively as large cutting tools (LCTs). Sangoan assemblages are typically thought to date to the later Middle Pleistocene^36^, though such ‘rugged’ or ‘heavy duty’ tools remain a persistent feature in some regions, such as Central Africa, throughout the Upper Pleistocene^37^.

Because the Sangoan was originally defined from sites found within the Victoria Basin and the present-day equatorial forests of Central Africa, there has been a long-standing perception that is represents a regionally constrained adaptation to woodland environments^32,33,38^. However, assemblages with a mosaic of LCTs and MSA-type artefacts from western^39^, northern ^40,41^ and southern Africa^42–44^, often of uncertain date or from insecure stratigraphic contexts, have since been attributed to the Sangoan, which has problematized and diluted the clarity of the term and what it refers to. The wide distribution of sites now assigned to the Sangoan, as well as the apparent technological variability within and between assemblages, also challenges the environmental determinism embedded within the original definition. It remains unclear whether the Sangoan is a discrete, early facies of the MSA, a broad transitional phase between the ESA and MSA, or whether many of these assemblages represent palimpsests of ESA and MSA technologies. Assemblages historically described as ‘Sangoan’ may indeed reflect all these possibilities. If the Sangoan is in fact a discrete technological entity, it is also uncertain whether it is widely distributed across Africa or whether it is more spatially constrained.

Stratified and robustly dated Sangoan occurrences are rare, complicating our understanding of their ecological and behavioural context. Kalambo Falls in Zambia represents one of the few stratified contexts, although the site lacked a reliable chronology at the time it was first reported^45,46^. Excavations at Site C North have since established the presence of LCTs and prepared core technologies between ~500-300 ka, however due to the small sample size the assemblage was instead described as transitional between Mode 2/3, rather than Sangoan^47,48^. To date, the only Sangoan artefacts associated with faunal and plant fossils were found at the site of Simbi (Kenya)^49^, however the available evidence indicates a grassland paleohabitat, further challenging the Sangoan-forest association. In addition, the stratigraphic layers from which the lithic assemblage derived have not been directly dated, but rather the site chronology is based on ^39^Ar/^40^Ar dates on volcanic minerals bracketing the Sangoan between 50 and 200 ka. In central and eastern Africa, assemblages attributed to the Sangoan are followed by or contemporaneous with MSA assemblages containing Levallois core and flake components and bifacially shaped lanceolate points, referred to as the Lupemban Industry after the Lupemba stream in Zaire^33,50^. At Twin Rivers in Zambia, the Lupemban has been suggested to date to sometime between ~266-132 ka^51,52^, although other Lupemban assemblages in this area derive from ambiguous or poorly documented contexts^33^. The uncertainty surrounding the relationship of these two industries has led to the use of the term Sangoan-Lupemban, and the Sangoan Lupemban Industrial Complex has also been used to describe assemblages in Kenya^33,50^. There is also a large degree of technological variation between sites defined as Sangoan, some of which appear to feature quite different technology, suggesting that the broad use of the term is problematic, as with many other technocomplexes or named stone tool industries (see e.g.^53,54^). For example, Kalambo Falls features a small tool component that is described as compatible with an MSA designation. Elsewhere, where this small tool component is absent or negligible, it has been suggested that the Sangoan may be a functional variant of the ESA^36^. It may even be possible that the ecological diversity of Africa’s interior regions (see e.g.^31^) is driving variability in toolkits that reflect environmental differences beyond the well-trodden regions of grassland and wooded grassland in southern, eastern and northern Africa. In the contemporary tropical rainforests around the Gulf of Guinea (Côte d'Ivoire, Ghana, Nigeria and Cameroon), assemblages with a small and large tool component described as ‘Sangoan’ are primarily defined by the presence of large cutting/heavy-duty tools and Levallois debitage. However, the characterization and definition of this cultural facies has long been debated because of a lack of reliable dates and secure stratigraphic contexts^39,55–57^.

There is also considerable underdetermination of site formation processes at many sites attributed to the Sangoan. The secondary and palimpsest nature of many of these contexts means that disentangling spatial and temporal associations from behavioural and non-behavioural processes is often not possible with the quality of the available data. Instead, the co-occurrence of LCTs with MSA-type artefacts is frequently used to assign assemblages to industries such as the Sangoan without critical appraisal of the depositional complexities or scales of time involved in their formation. It is likely not a coincidence that many assemblages designated as Sangoan derive from highly time-averaged deposits at open-air sites, usually in contexts with low rates of sediment accumulation and low artefact densities, and often with limited stratigraphic control and chronological resolution. The issues of site formation and temporal resolution, combined with the focus on the LCT component and a general lack of reporting of assemblage-scale technological information, mean that it is often hard to determine the behavioural and technological context of Sangoan sites. Additionally, the Sangoan and MSA are rarely found in the same sequence, making it difficult to contextualise any cultural, chronological and palaeoenvironmental changes through time. Finally, it has been noted that the majority of these sites are found on the edge of contemporary tropical rainforest in regions that may have become grassland during drier climatic periods in the past^30,36^, making ‘on-site’ palaeoenvironmental assessment essential.

It is therefore important that these terms are used with caution and great circumspection, particularly in regions beyond their traditional usage. Whether the LCT component of either transitional ESA-MSA and MSA assemblages reflects ecological conditions is, however, a slightly different question. Despite the association of the LCT component of Sangoan assemblages with woodworking activities^58–60^, very few studies have examined micro-wear traces on these tools to test this hypothesis, and consequently this functional association has not been confirmed^61^. A use-wear study on Lupemban bifacial tools from Equatorial Guinea indicated they were used not only for working wood material but also for a diverse range of other activities^62^. To date, micro-wear on Sangoan tools has only been examined at the site of Sai Island in Sudan^63^. The micro-wear patterns on these LCTs do not appear to support a woodworking or plant processing function, but rather indicate digging in a hard substrate^63^. Sai Island represents a very different ecological setting to sites in West and Central Africa, however, and caution is required when extrapolating these results to sites such as Bété I and III at Anyama, where a functional association with the forested environment remains a possibility, although it is unproven to date.

## 1.3 Previous excavations and work at Bété I

Anyama is located toward the northern extent of the sediment basins that extend inland ~50km from the present coast line, beyond which sediment cover overlying Pliocene weathering crusts and more ancient peneplains is highly limited^39,64^. The modern drainage network was likely formed after the formation of Pliocene surfaces, but these still exert control over the location of major drainages^39^. Whereas the sediment basins close to the coast are dominated by Holocene marine sediments, further inland they comprise gravelly, loosely sorted sandy loams of the Continental Terminal overlain by homogenous clayey *Terre de Barre* red sands. These deposits, and their subsequent incision, drive modern patterns of relief in the landscape surrounding Anyama sites.

The archaeological sites at Anyama (**Figure S1**) were first discovered in the early 1980s by Chenorkian and Paradis^65,66^, through the investigation of an old quarry that had produced steep sediment sections for inspection. The local bedrock was identified between 5-7m above the present valley floor, where the small Bété River flows, which subsequently lent its name to the archaeological sites. Lithic artefacts, including side-scrapers, flakes, core-axes, picks, Levallois and multiplatform cores, were identified in ~10m of red *Terre de Barr*e sediments overlying ~2m coarse sand and gravel unit. Notably, they suggested the deposits were likely Quaternary in age. The artefacts were reported as stratified beneath later ‘Epipalaeolithic’ and Neolithic material^65,66^. However, although the combination of LCTs with a Levallois core and flake component elicited comparisons with the Sangoan, Chenorkian and Paradis^65,66^ advocated caution regarding the use of this term in West Africa, and doubted its equivalence to the use of the term elsewhere. The quarry sequence was proposed to represent an alluvial terrace formed during a marine highstand, potentially linked to the Last Interglacial^66^.


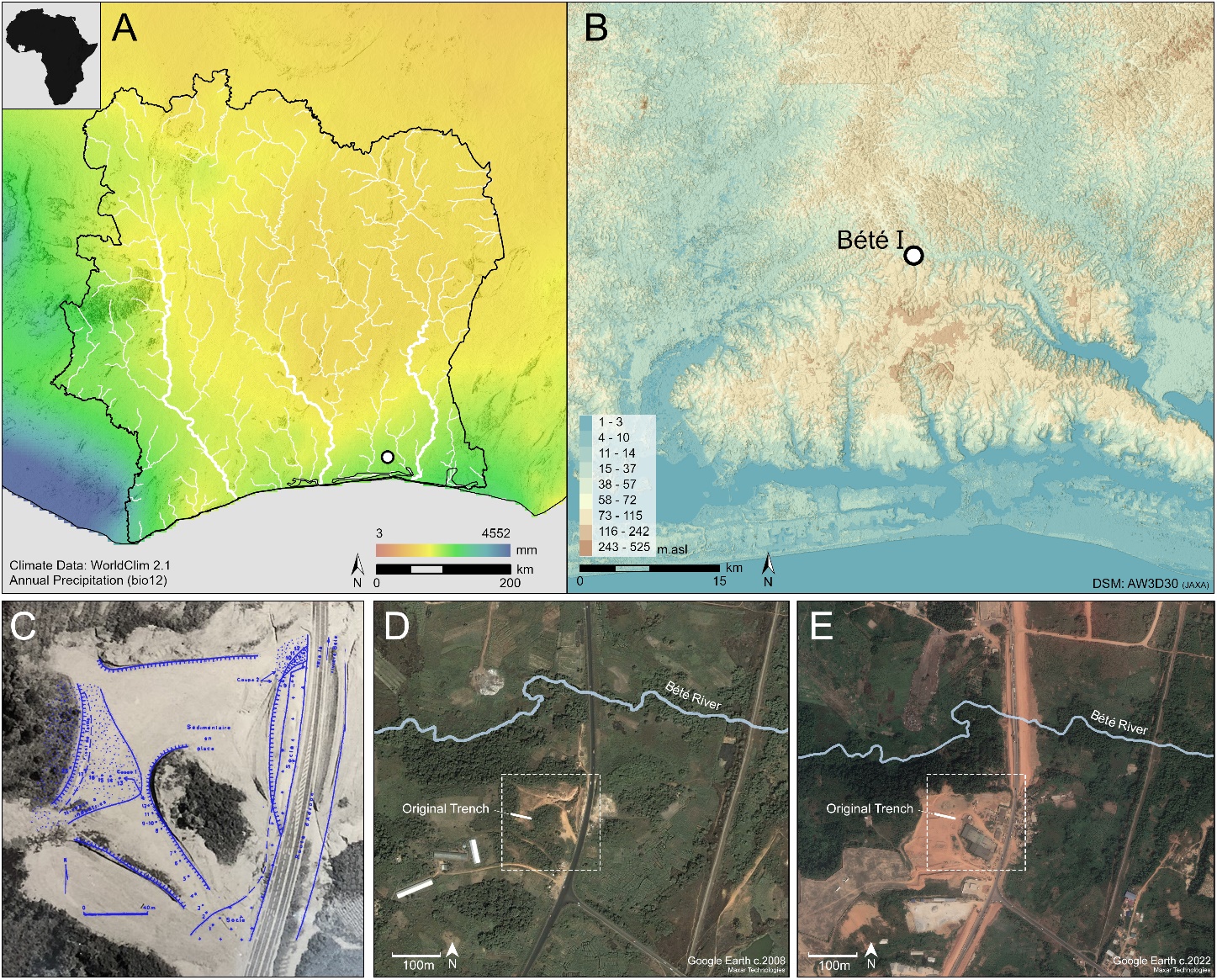


**Figure S1**: **Location and details of quarry site at Anyama.** (A) Map of Côte d'Ivoire, showing modern annual precipitation^67^and river systems, (B) digital surface model showing location of Bété I within the wider region, (C) historical aerial imagery of the Anyama Quarry with Bété I sequence (c. 1980s, courtesy of Prof. Guédé) with site plan^64^ showing trench locations. (D) Google Earth imagery from 04/2008, showing conditions at the site and the Bété River, and (E) imagery from 01/2022, showing the expansion of the quarry and destruction of the site. Maps made using ESRI ArcGIS Pro 3.2. Climate data in A and B are from^68^.

Subsequent investigation by Guédé and Tastet (1986) further clarified the extent and diversity of sediment deposits across the quarry site. This included including examination of deeper sequences of Terre de Barre deposits, ca 17m thick, and 9m of Continental Terminal deposits, and differentiating the deposits into 6 units, labelled A-F, resolved in part through the initial excavations at the site (**Figure S2**), focused on Unit C. Investigation of the valley floor deposits revealed a 2m thick sequence with a 1.5m silt horizon returning radiocarbon dates of 3394-3686 cal BP (3300±60; Geotop-728) and 3276-4067 cal BP (3390±140; Ly-2672), and an underlying gravel deposit dating to 9155-9550 cal BP (8460±80; Geotop-729), overlying unaltered bedrock. This may be indicative of a terminal Pleistocene or early Holocene incision for the present drainage, and potentially for the terrace deposits.


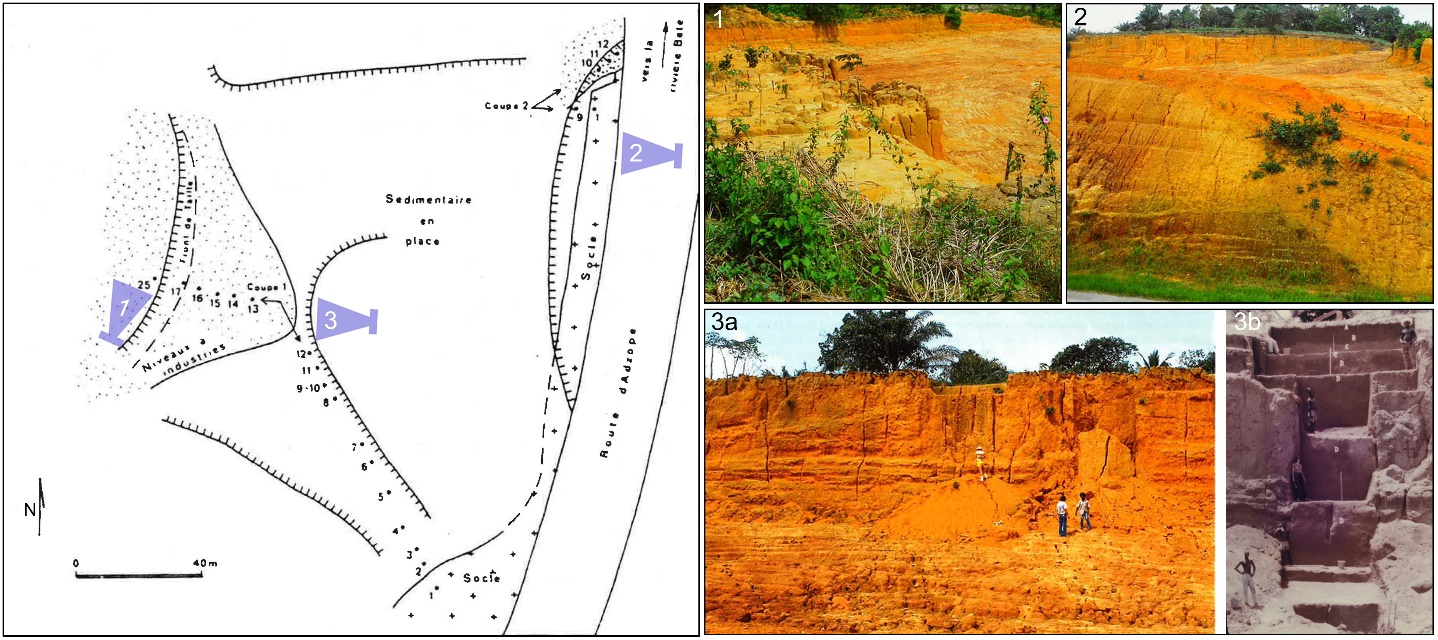


**Figure S2**: **Historical photos of excavations at Bété I.** Site plan and photos from^64^: (1-3a) Photos of Bété I in November 1983 taken by P. Allsworth-Jones during a visit to the site, and (3b) photo of main trench at Bété I during excavation in 1990s (courtesy of Prof. Guédé).

Investigations by a joint Russian-Ivorian mission conducted further detailed work at the site until the early 1990s, with a suite of articles published in Russian up until the early 2000’s^39^. During the 1993 excavations, 14 geological horizons were recorded at across the Anyama quarry walls and step trenches, with Units A-F retained to describe the sediments of the excavation area. This expanded on the previous excavation site, reaching the base of Terre de Barre/Unit D deposits, revealing the contact with Continental Terminal/Unit E deposits at the base of the 15.7m deep trench (**Figure S2**). The sediment sequence was studied by S. N. Sedov and including “particle size analysis and a complex of chemical analyses including determination of gross chemical composition by X-ray fluorescence, pH of aqueous and saline (KST PM) extracts by potentiometric method, content of exchangeable hydrogen by Kappen, exchangeable bases in the acetate-ammonium extract, humus by Tyurin, iron and aluminium compounds extracted by oxalate (by Tamm) and dithionite (by Mera-Jackson) extracts. In addition to chemical analyses, polarisation microscopes were used to examine the material in petrographic and micromorphological terms” (^39^:11-12), with key conclusions reported as personal communications in^39^; here we summarise the salient points of Sedov’s findings.


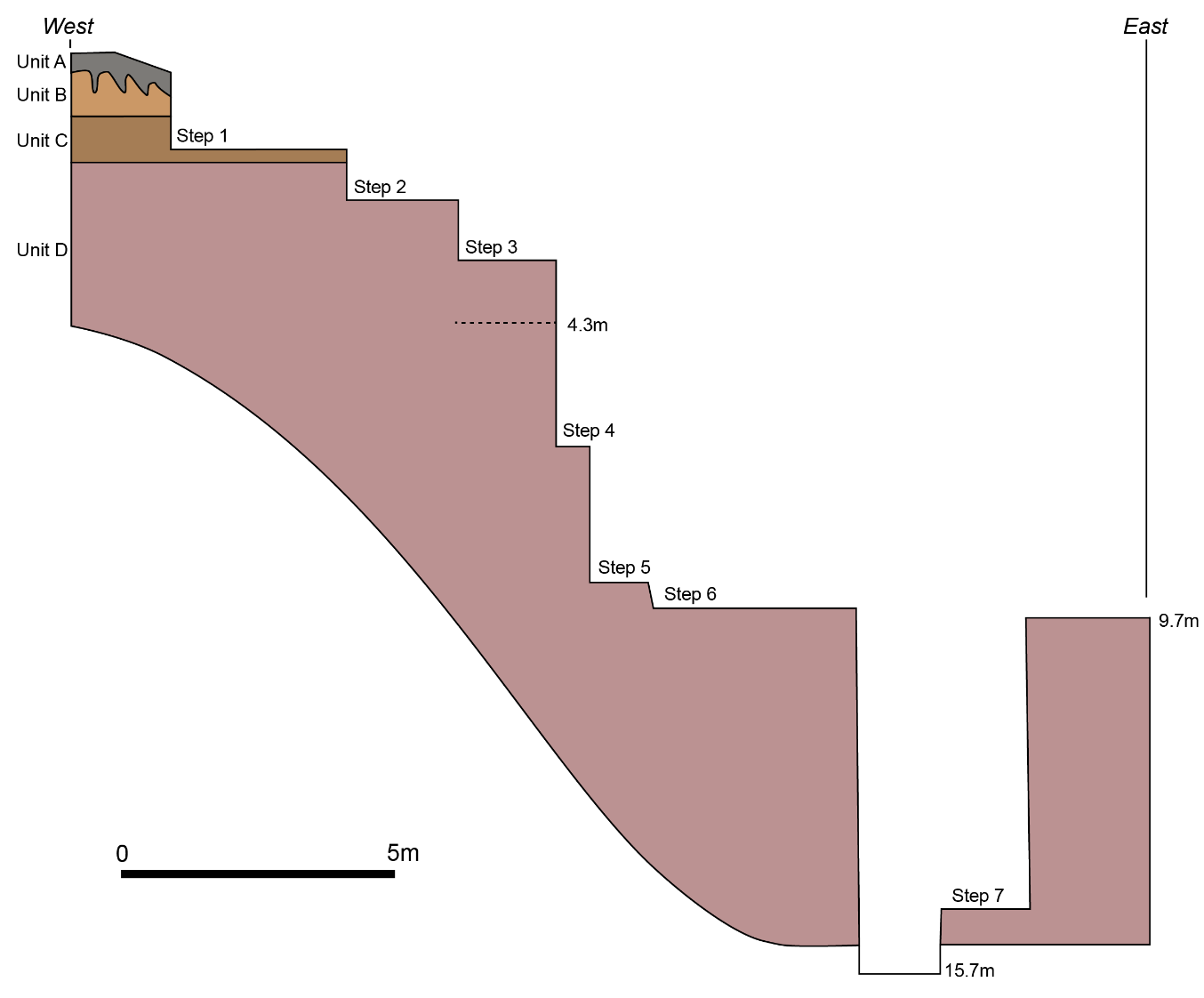


**Figure S3**: Longitudinal section across the Bété I excavation area (Steps 1-3), and the cleaned Quarry wall (Steps 4-6) and Test Pit 1 (Step 7), redrawn from Figure 6 in Lioubin and Guédé^39^, differentiating the Terre de Barre deposits of Units A-D and highlighting the steps re-examined in this study (Steps 1-4) and the lowermost artefact bearing level at 4.3m.

The Precambrian basement was identified by Sedov as chloritic shale, which is directly overlain by two weathering horizons, together identified as Unit F/Layer 1, with yellow-brown loams retaining original structural elements of the parent rock, and white spotted red loams lacking structural features but sharing quartz veins, both having undergone extensive weathering. An erosive contact is observed with overlying deposits, with up to 9m of pebbles and coarse sands attributed to the Continental Terminal and identified as Unit E/Layers 2-7. Two members were differentiated, with a lower member described as a better sorted, homogeneous sand (Layers 2-4), often lacking gravel, whereas the upper member is a polydisperse sand unit with silts and gravels, the latter typically weakly rolled vein quartz fragments (Layers 5-7). These were interpreted by Sedov to have formed as a result of temporary water flows with intensive reworking of sediment deposits.

The contact between Unit E and Unit D (Layers 8-11) likely marks erosion onto a ferruginous cementation zone and is directly overlain by a thin gravel layer, marking the only natural gravels present in the Terre de Barre deposits overlying Unit E. Units D and C (Layer 12 *Terre de Barre* sediments are described as sandstone with increasing presence of finer fractions (i.e. silts, clays) with depth from surface, with a maximum depth of 17m. The lower layers of Unit D comprise coarser dull red sediments with yellow spots at the base (Layer 8) overlain by more homogenous and brighter coloured coarse red sediments (Layer 9). The upper layers of Unit D and Unit C (Layers 10-12) share a reddish colour with increasing frequency of white spots with dark red halos towards the surface and were interpreted as root pores or small burrows. The uppermost deposits reported from the sequence comprise a compact brown subsoil (Unit B; Layer 13) overlain by a loose lumpy humus horizon (Unit A; Layer 14).

Sedov argued that differentiation within the *Terre de Barre*/ Unit C-D sequence was the result of *in situ* pedogenic processes, with lassivage (removal of clays in suspension) and eluvial-gley processes (removal of iron compounds) influencing downward sediment composition in the sequence. Downward transport of clays was also identified through the presence of sintered clay films observed in thin sections. Petrographic studies indicated that sand fractions were predominately composed of quartz, with the relatively low birefringence, high aluminium content, and low cation exchange capacity suggestive that finer components are dominated by kaolinite. Sedov concluded that these strata were likely formed from the redeposition of transported ancient weathering crusts, with the slow alluvial accumulation of sediments during which each layer would have been exposed as a surface horizon.

As reported by Sedov, the upper 15 m of Unit D deposits contained what they initially termed as Sangoan artefacts in four layers, documented as coming from the upper part of layer 9, continuing into layers 10 to 12. Layer 13 corresponded to the 1m thick red sands of the Unit C, with typical MSA artefacts. Layers 14 corresponded to the 60 cm thick brownish sands of the Unit B with microlithic tools, and the 20 cm humic horizon in Unit A on the top containing pottery, supporting Chenorkian and Paradis^65,66^ initial observations.

The Russian-Ivorian mission dated the sediment sequence by applying the Radiothermoluminescence (RTL) dating method on sandy-clay sediment^39^. Of the three ages obtained, two were respectively at the base and top of the Unit E (1400 ± 350 ka and 309 ± 62 ka). The single RTL age from Unit D at ca 1.5-2 m below the deepest quartz tools provided a *terminus post quem* (i.e maximum age) of 254 ± 51 ka for cultural deposits at the site. This age, indicated that the assemblages of the Unit D may have been formed at the end of the Middle Pleistocene. However, the sediment in which the archaeological assemblages are embedded were not directly dated. Importantly, the detailed parameters employed for the age calculation were not provided in the monograph of the archaeological sites of Anyama^39^, precluding thus any critical evaluation of the published results. Consequently, these dating results should be treated with extreme caution and be regarded as mostly indicative.

Similarly, there was an attempt to understand the palaeoenvironments associated with Pleistocene archaeology. The geological data obtained from Unit D^39^ appeared to suggest a relatively slow rate of accumulation. Phytolith analyses published by Lioubin and Guede^39^ appeared to show open and semi-open environments based on the predominance of herbaceous plants (mainly cereals) for most of the unit. However, in most samples (including from the middle and lower parts of the deposit) cuticular casts were found - specific phytoliths typical only of the surface horizon. This indicated to the authors that the accumulation of the *Terre de Barre* sediment proceeded rather slowly, corresponding to a dry period, and each layer was for some time a surface horizon where humans may have settled^39^.

## 1.4 Stone tool technology at Anyama

The stone tool assemblages were described in Lioubin and Guédé^39^ and illustrated examples are displayed in **Figure S5**. The published analyses included counts of typological categories but technological data that would enable comparative study of these artefacts were not presented. Most of the tools were lost during the Civil War (2010-2011), limiting any revaluation of the site to the available documentation or new field sampling. The destruction of the original site between 2020 and 2021 has further curtailed our ability to examine behavioural records from the site^69,^ or to further re-assessing the technological characterization of the assemblages. Given these constraints, below, we synthesise previous reports of the stone tool assemblages from Bété I. In addition, other localities that have been excavated, such as Bété II, III and IV are included below.

Stone tool assemblages were originally recovered from Units B, C and D, and peaks in their frequency correspond closely with the presence of artefacts evident in the examined sediment section during our 2020 campaign. The majority of artefacts were found within the upper 4.3 m of sediments, with isolated artefacts identified below to a total depth of 6.4 m. Raw material use is focused on local quartz, comparable to those identified in plate-like veins at the junction of Quaternary and Precambrian deposits. Higher quality materials with fewer visible flaws appear to have been preferentially selected in the Unit C assemblage, while artefacts from the Unit D assemblage were manufactured on larger quartz clasts with more abundant flaws. A small assemblage of 50 microlithic tools was recovered from 12 m^2^ of the Unit B deposits (1985), including chisels, scrapers, and three cores. The deposits of interest in this study are Units C and D.

Unit C yielded the largest assemblage from Bété I, and was primarily excavated in 1982-3 over 32 m^2^ (n=477), and in 1986 over 12 m^2^ (n=63 [excluding debris]). Further investigation at Bété II and III yielded a further 9 and 59 artefacts respectively from Unit C. At all three locations, core technology was focussed on radial reduction schemes, typically from heavily reduced cores, with fewer unidirectional cores, supplemented by the presence of a bidirectional core at Bété I and III and Levallois cores-on-flakes at Bété III. Several flakes with faceted platforms (*chapeau de gendarme*), as well as two Levallois points found at Bété III further supports the presence of Levallois reduction. Only a single flake of blade proportions (length>2x width) was found across these assemblages, indicating the absence of dedicated focus on laminar reduction. A range of retouched tools are present at Bété I, including bifacial points, bifacial point tips, scrapers, notched tools, chisels, a pick, and a chopper, with comparable inventories at Bété II and III. The presence of fine knapping debris suggests that lithic reduction and discard activities were undertaken at the site, and indicates that no substantive post-depositional winnowing of the assemblage has occurred. The young age of the Unit C deposit, along with the Levallois elements and the bifacially retouched points and tips, suggests that a MSA designation is appropriate for this assemblage. The presence of a pick and a chopper is noteworthy and may be indicative of a specific and persistent functional requirement for heavy duty tools and/or adaptation to the local forested environment, although it is not currently possible to confirm this.

We have identified a number of peaks in artefact frequency in Unit D that can be observed at the D2/3 interface, in the middle of D2, and, to a lesser extent, at the base of D1. The appearance of concentrations of artefacts at the interface between sediment horizons in Unit D is suggestive of more intensive occupations at the site during hiatuses of deposition. In total, 513 artefacts were excavated at Bété I from Unit D and are reported alongside an additional 47 artefacts from sites II-IV as a single assemblage. The majority of artefacts recovered were considered flakes, fragments or debris, alongside 17 cores, an anvil, and 72 tools. Four large cores (17-12 cm) include a centripetal Levallois core, three cores with discrete striking platforms but few removals, and a bidirectional core, whereas smaller cores include eight unidirectional cores, a disc-shaped core, a sub-prismatic core and an amorphous core. An additional Levallois core was recovered prior to excavations at the site[^36^](https://www.zotero.org/google-docs/?UW6avi), but is also attributed to Unit D. Nine large flakes (16-10 cm) are reported, with the majority of debitage typically smaller (8-3.5 cm). The debitage is considered to have resulted from production of diverse heavy-duty tools, with some selected for use or retouching, indicated by the presence of ten scrapers. Heavy-duty tools were also recovered, including a diverse array of bifaces (n=15) and picks (n=20), both with pointed and chiselled ends present, core scrapers (n=8), choppers (n=6), and polyhedrons (n=2). The available evidence thus indicates that Unit D contains both heavy duty LCTs as well as prepare cores component, although the latter is a very minor part of the assemblage. Given that the loss of the stone tool collection precludes a re-evaluation of this assemblage, as well as the aforementioned issues with the Sangoan’, we have opted against the use of this term here. Instead, we recognise that the Unit D assemblage is unique in terms of its age, technological character, and ecological context.


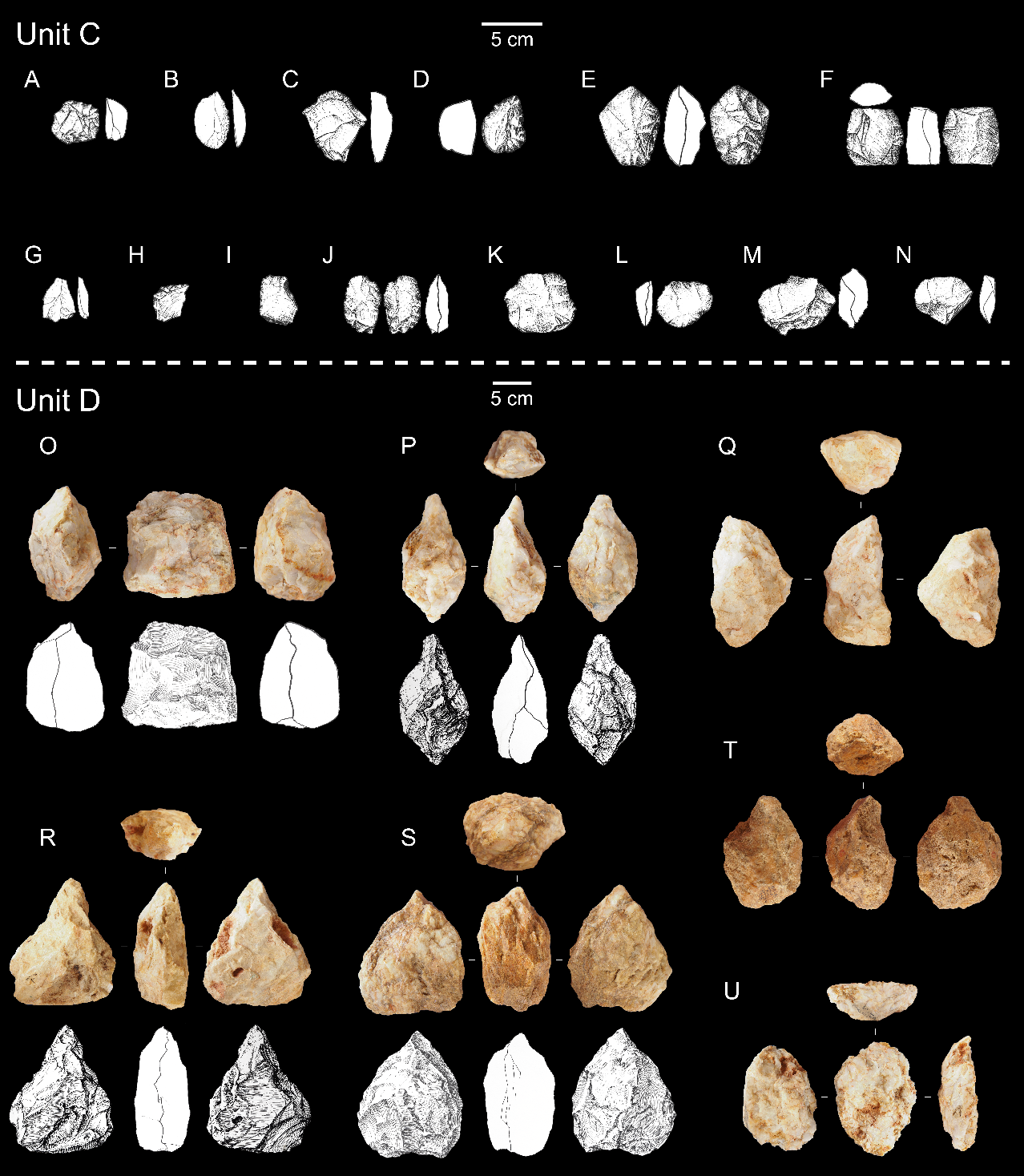


**Figure S4**: Stone tools from Units C and D at Bété I and III from Lioubin and Guédé^39^, and photos taken of the remaining artefact collection at the Institut des Sciences Anthropologiques de Développement (ISAD) in 2021. Unit C: A) ‘end-scraper’, B) ‘point’, C) ‘end-scraper à museau’, D) ‘double-ended carinated end-scraper’, E) ‘small handaxe’, F) ‘fragment of a bifacial foliate piece’, G) ‘point Levallois’, H) ‘combination tool’, I) ‘end-scraper with spine’, J) ‘short foliate biface’, and K-N) ‘cores’. Unit D: O) ‘side and end chopper’, P) ‘biface - trihedral’, Q) bifacial LCT (our term), R) ‘pick with double-flat cross-section of the body and centred quadrihedral distal point’, S) ‘pick with double-flat cross section of the body and centred trihedral distal point’, T–U) bifacial pieces (our term). A-N and O, P, R and S are from Lioubin and Guédé[^36^](https://www.zotero.org/google-docs/?QIm94X).

# SI 2 – Sedimentology

In 2020, we revisited the original site and cleaned the uppermost four steps of the previously excavated trench at Bété I spanning the top 5.65m of the sediment sequence for recording and sampling (**Figure S5**) Field recording of sediment units matched directly with the sequence as reported by^39^, comprising four discrete sediment units referred to as Unit A-D. To refine the description of the depositional sequence at Bété I and interpretation of alluvial origin to the sediments, we applied laser particle size, loss on ignition, and magnetic susceptibility analyses (see below), with the synthesized results presented as **Figure 2.** Data acquisition are detailed in **Table S1**.

For laser particle size analysis, sieved sediment samples (~1g, <2mm) were bathed for 24 hours in sodium hexametaphosphate (0.5%) solution and agitated in an ultrasonic bath, with samples rinsed in purified water prior to analysis in a Malvern Mastersizer 3000. Characterisation of the grain size results were conducted using Gradistat^70^. For loss on ignition studies, sediment samples (~10 g) were weighed (to three decimal places, i.e. 0.001g) and heated in a muffle furnace to 105°C, 550°C, and 950°C (allowing the sediments to cool to 105°C for weighing between steps) to calculate the proportions of water, total organic matter, carbonates, and mineral residue. Magnetic susceptibility was measured in the laboratory using a Bartington MS3 magnetic susceptibility meter coupled with the MS3B sensor to analyse ~12 g samples, weighed on precision scales to enable calculation of mass specific values presented as Χ_mass_ (10^-8^m^3^/kg), with data collected using BartSoft v4.2.

Unit A is comprised of a ~27 cm thick unit of brownish grey, bimodal, very poorly sorted medium to fine silty sand with a substantial organic component (including visible charcoals). Unit B is comprised of a ~30 cm thick pale greyish/brownish yellow, bimodal, very poorly sorted fine silty to muddy medium sands. The sediment is typically comprised of 2% organic components. Magnetic susceptibility values rise from 23.8 to a peak at 25.5 before marking the start of a decline with depth toward the Unit C sequence. A level but gradual contact is observed with the underlying sediment unit. Unit C and is comprised of a ~1m thick deposit of mid brownish red, trimodal, very poorly sorted muddy medium sands. Minor bioturbation was identified and attributed to the presence of termite activity, whilst multiple lithic artefacts were also observed in the sections. A minor increase (2.3 to 3.1%) in organics is observed with depth, whereas magnetic susceptibility decreases (21.5 to 19.1 10^-8^m^3^/kg) gradually with depth.

In the field, the contact with underlying deposits was observed as diffuse and obfuscated by a gradual increase in mottling, and this is supported by results of laboratory analysis which identify gradual decreases in particle size and magnetic susceptibility and increases in organic components. On this basis we identified a discrete ~25 cm thick transition horizon, labelled Unit C-D, comprised of trimodal, very poorly sorted medium sandy muds with mean particle sizes of 204.2 to 161 µm, 4.5-5.1% organic components and magnetic susceptibility values decreasing from 17.5 to 16.7 in this unit.


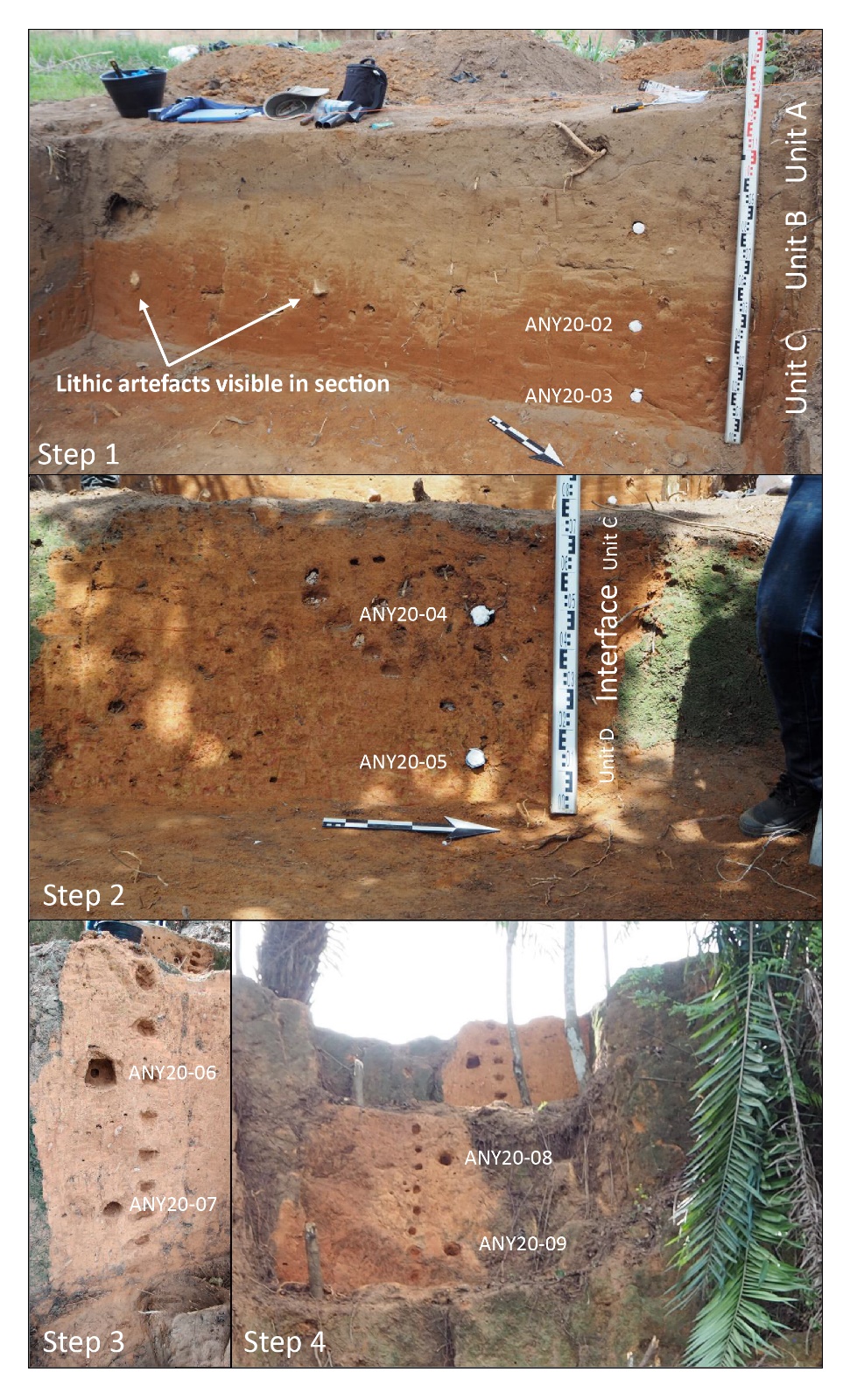


**Figure S5:** Photos illustrating sediment exposures and geochronological sample locations on the uppermost four steps of the step trench at Bété I.

Unit D extends throughout the lower examined sequence, where mottling of pale yellowish, pinkish and orangish red sediments vary and include dappled, linear and haloed features, which may result from combinations of weathering and past bioturbation. Isolated lithic artefacts were identified at multiple levels in the lower two trench steps. Sedimentological analyses enable three discrete horizons to be distinguished within Unit D. The uppermost, Unit D1, is composed of trimodal, very poorly sorted medium sandy muds to clays, marking a continued decrease in mean particle size as a result of sharp increase in proportion of fine clays (size) and decrease in silts, as well as a notable peak in organic components of 6.8%, and continued gradual decline in magnetic susceptibility values. Units D2 and D3, both characterizable as bi- or trimodal very poorly sorted medium to fine sandy clays, continue gradual decreases in mean particle size and organic components with depth. Unit D2 marks a distinct departure from overlying and underlying deposits due to a pronounced peak in magnetic susceptibility values at 23.66 x 10^-8^m^3^/kg.

These findings are consistent with those of previous researchers, and particularly align with the detailed descriptions of the sediment sequence by Sedov, presented by Lioubin and Guédé^39^. The downward increase in the clay fraction is consistent with the suggestion for lassivage processes, with the broad similarity in sand fractions supporting the interpretation of a consistent alluvial depositional regime throughout the upper Terre de Barre deposits (Units A-D) overprinted by in situ weathering processes. Our age estimate for Unit C deposits helps to constrain the longevity of this depositional sequence, suggesting continued alluvial deposition at the end of the Pleistocene, and combined with an early Holocene age for deposits in the present valley floor (see above), indicates incision of this sequence coincident with the onset of the Holocene. More limited evidence of weathering processes is evident in Unit C deposits, than in underlying Unit D deposits. While this may be a factor of time, other potential influences include differences in climatic regime, with Unit D deposits forming during or exposed to warmer, wetter conditions during MIS 5, a shift in ground water conditions relating the incision of the modern valley may also play a role. The gradual transition we see between Units C and D, termed C/D, may potentially reflect patterns of bioturbation, a weathering front associated with groundwater levels, or potentially both.

**Table S1:** Results of sedimentological studies based on laser particle size, loss on ignition, and magnetic susceptibility analyses. The IDs refer to the numbers along the sequence displayed in **Figure 2**. These analyses for units A and B were carried out in order to provide information on the entire sequence, even though units C and D are of primary interest in this study.

| **Sample number** | **Depth**  **(m)** | **Unit** | **Organic Content (%) LOI** | **LF Average (Mag Sus,** **10^-8^m^3^/kg)** | **All Clays (%)** | **Silt**  **(%)** | **Sand**  **(%)** |
| --- | --- | --- | --- | --- | --- | --- | --- |
| 1 | 0.1 | A | 2.25 | 20.20 | 7.56 | 40.79 | 51.65 |
| 2 | 0.2 | A | 2.28 | 20.62 | 9.85 | 32.39 | 57.76 |
| 3 | 0.3 | A | 8.41 | 22.04 | 8.14 | 31.41 | 60.45 |
| 4 | 0.4 | B | 2.00 | 23.30 | 8.95 | 23.11 | 67.94 |
| 5 | 0.5 | B | 1.98 | 25.00 | 12.42 | 27.93 | 59.65 |
| 6 | 0.6 | B | 2.01 | 23.38 | 10.97 | 21.68 | 67.35 |
| 7 | 0.7 | B | 1.63 | 21.90 | 11.91 | 21.61 | 66.48 |
| 8 | 0.8 | C | 2.33 | 19.88 | 15.71 | 27.39 | 56.90 |
| 9 | 0.9 | C | 2.58 | 20.94 | 15.44 | 23.76 | 60.80 |
| 10 | 1 | C | 2.83 | 19.28 | 20.43 | 20.05 | 59.52 |
| 11 | 1.1 | C | 2.82 | 17.58 | 20.79 | 18.73 | 60.48 |
| 12 | 1.2 | C | 3.02 | 18.32 | 22.32 | 17.27 | 60.41 |
| 13 | 1.3 | C | 3.11 | 18.48 | 22.59 | 17.24 | 60.17 |
| 14 | 1.4 | C/D | 5.05 | 16.62 | 25.7 | 24.01 | 50.29 |
| 15 | 1.5 | C/D | 4.49 | 15.98 | 30.36 | 21.22 | 48.42 |
| 16 | 1.6 | D1 | 5.42 | 14.52 | 32.67 | 23.6 | 43.73 |
| 17 | 1.7 | D1 | 5.09 | 14.60 | 38.8 | 22.65 | 38.55 |
| 18 | 1.8 | D1 | 6.30 | 13.44 | 39.33 | 20.65 | 40.02 |
| 19 | 1.9 | D1 | 6.82 | 13.88 | 39.46 | 15.82 | 44.72 |
| 20 | 2 | D1 | 5.63 | 14.00 | 45.54 | 17.00 | 37.46 |
| 21 | 2.2 | D1 | 5.59 | 14.26 | 41.37 | 13.05 | 45.58 |
| 22 | 2.4 | D2 | 4.65 | 16.12 | 41.82 | 12.28 | 45.90 |
| 23 | 2.6 | D2 | 4.63 | 18.44 | 40.57 | 12.49 | 46.94 |
| 24 | 2.8 | D2 | 4.89 | 21.54 | 39.89 | 13.04 | 47.07 |
| 25 | 3 | D2 | 4.58 | 23.66 | 41.53 | 14.10 | 44.37 |
| 26 | 3.2 | D2 | 4.53 | 19.72 | 41.65 | 12.96 | 45.39 |
| 27 | 3.4 | D2 | 4.40 | 16.94 | 43.49 | 12.16 | 44.35 |
| 28 | 3.6 | D2 | 4.74 | 15.72 | 42.78 | 12.27 | 44.95 |
| 29 | 3.8 | D2 | 4.48 | 13.64 | 46.14 | 13.72 | 40.14 |
| 30 | 4 | D3 | 4.37 | 9.46 | 41.63 | 12.37 | 46.00 |
| 31 | 4.2 | D3 | 4.98 | 10.44 | 48.54 | 15.58 | 35.88 |
| 32 | 4.4 | D3 | 3.94 | 11.20 | 42.91 | 13.06 | 44.03 |
| 33 | 4.6 | D3 | 4.68 | 10.18 | 38.78 | 16.36 | 44.86 |
| 34 | 4.8 | D3 | 4.22 | 10.38 | 39.91 | 14.29 | 45.80 |
| 35 | 5 | D3 | 4.37 | 9.84 | 42.08 | 13.51 | 44.41 |
| 36 | 5.2 | D3 | 4.83 | 9.64 | 40.43 | 14.39 | 45.18 |
| 37 | 5.4 | D3 | 4.55 | 9.16 | 41.42 | 11.72 | 46.86 |

# SI 3 - OSL and ESR dating of quartz grains

## 3.1 Material

Eight sediment samples (**Table S2**) from the Bété I excavated sequence were collected using opaque metal tubes (25 cm in length and 4 cm in diameter), sealed with adhesive tape, and packed in black light-opaque bags to preserve the light-sensitive OSL signal during transport. Two sediments were sampled from Unit C. one at the transition between Units C and D, and five sediments from Unit D. The samples were taken from the sections of the first excavations of the site and named “steps”. The samples cover the uppermost five metres of the stratigraphic sequence. Unit C is a homogenous but poorly sorted mid-brownish red silty sand. Regarding Unit D, the sediments are a mottled poorly sorted yellow pink and orangish red silty sand; mottling may represent *in situ* weathering with differing patterns of chemical mobility; alternatively, bioturbation could have occurred during the process of weathering; mottling appears dappled and distinct from its appearance lower in the sequence. The division of the Unit D in subunits D1, D2 and D3 is based on the new sedimentary characterization detailed in **SI 2**.

Feldspars were looked for within all samples in order to utilise the post-IR-IRSL protocols which have higher saturation limits and are more suitable for older samples^71^ but the samples were found to contain none. Sample preparation was carried out at the Sheffield Luminescence Dating Laboratory (University of Sheffield, UK). The samples were prepared under subdued red lighting following the extract and clean coarse quartz procedure outlined in Bateman and Catt^72^. This included use of HCI solution to remove carbonate material, H_2_O_2_ to remove organic material and dry sieving to separate a reduced size fraction of 180-212 µm. Sodium polytungstate solution (2.70 g/cm^3^) and HF etching was used to isolate and clean quartz from other minerals.

**Table S2:** List of sediments samples analyzed in this work. The depth of each sample relates to the estimated ground surface prior to initial excavation at the site in the 1980's/90s

| **ID Field** | **OSL lab code/ESR lab code** | **Unit** | **Depth (m)** |
| --- | --- | --- | --- |
| ANY20-02 | Shfd20130 | C | 0.85 |
| ANY20-03 | Shfd20131/ANY20-03 | C | 1.15 |
| ANY20-04 | Shfd20132/ANY20-04 | Interface C/D | 0.90 |
| ANY20-05 | Shfd20133/ANY20-05 | D1 | 1.24 |
| ANY20-06 | Shfd20134 | D2 | 1.90 |
| ANY20-07 | Shfd20135 | D2 | 2.60 |
| ANY20-08 | Shfd20136/ANY20-08 | D3 | 3.60 |
| ANY20-09 | Shfd20137/ANY20-09 | D3 | 4.60 |

## 3.2 Luminescence analyses

Given the alluvial depositional context described above for the sampled sediments, the potential for inclusion of incompletely bleached grains was recognised. Whilst measurement of individual grains could have been made, such measurements suffer from an intrinsically weaker measurable signal above background with few grains providing measurable signals and often high uncertainties. In low dose environment (as found at this site – See section 3.4 for details), beta dose heterogeneity is also a possible problem^73^ which at the single grain level can lead to age under-estimations if minimum age models are applied. In contrast, measurements at the single aliquot level, containing 1500-2000 grains measured simultaneously may offer stronger measurable signals, but suffers from signal/grain averaging effects making it difficult or impossible to separate out poorly bleached and erroneously high palaeodose measurements from those relating to true burial age^74^. Based on these considerations, we initially decided to follow an intermediate approach that would benefit from the advantages of both procedures, by measuring very small aliquots containing around 100 grains. Previous single grain measurements (n=100) from a sample at an adjacent archaeological site located about 100 m away in the same sedimentary environment and catchment, showed that 50% of the signal would come from 3 grains, and 75% of the signal from <7 grains (**Figure S6**). Thus, whilst gaining on the one hand from a better signal to noise ratio with multi-grain aliquots, on the other hand we sought to minimise grain averaging effects to a level where if aliquots did contain incompletely bleached grains, these would be discernible in the resultant palaeodose replicate distribution.

**Figure S6**: Single grain quartz OSL shine down data from a sample from an adjacent archaeological site less than 100m away from the Anyama site showing how the bulk of the OSL signal is derived from very few gains (<7). Results shown with different line colours are from four different 100 grain single grain aliquots with the grains ranked by their OSL signal size.

In light of the above, for OSL measurement, quartz grains (180-212 µm) were mounted as a 2 mm diameter monolayer on 9.6 mm diameter stainless steel disks to form aliquots. All measurements were performed with a Risø DA-18 luminescence reader with a calibrated Sr^90^ beta source, blue LEDs (emitting at 470±30 nm) for OSL stimulations and luminescence detection was through a Hoya U-340 filter. Twenty-four multi-grain aliquots per sample were measured. Equivalent dose (D_e_) measurement was by the single aliquot regenerative (SAR) approach with OSL stimulations for 60s at 125^o^C^75,76^. Elsewhere, others have shown the capability of the quartz SAR protocol to accurately date sediments over 200 ka with D_e_ values in excess of 250 Gy^77^. The most appropriate SAR preheat temperature of 200 °C was derived from a dose recovery preheat plateau test on Shfd20131 (**Figure S7**), for which a dose recovery ratio close to unity was obtained (0.98 ± 0.02). An IR ratio test (as per^78^) validated the absence of any feldspar signal in the prepared quartz extracts. Data were analysed using Analyst Software^79^ using OSL from the first 1.2s of stimulation as the signal integral and OSL from the last 12s as the background integral. All samples display good luminescence characteristics with signal dominated by the rapidly decaying fast component (**Figure S8-A**). The SAR data was best fitted with a single saturating exponential curve for the youngest samples (Shfd20130-133) and a single saturating exponential + linear curve for the older samples (Shfd20134-137). D_e_ values from each aliquot were accepted based on the following criteria: i) a measurable OSL signal 3 sigma above background, ii) SAR growth curve within 1σ errors of regeneration points, iii) recycling values within ± 10 % of unity, and iv) error on the SAR test dose < 10 %. Despite measurement of large D_e_ values for samples from Unit D, SAR growth curves for most aliquot/samples showed good and continued OSL signal growth with increasing laboratory dose (**Figure S8-C**). Finally, samples Shfd20133 and Shfd20137 showed some aliquots in saturation (4/24). Implications are discussed below in Section 3.5.5.

Luminescence measurements data were analysed using Analyst Software^80^ with further statistical analysis and plotting of the D_e_ data (see section 3.5.5) using R studio using RLumShiny^81^ and Luminescence^82^ R packages.

Not withstanding the above, for comparative purposes all samples were also measured at the single grain level. Single grain measurements were conducted with a Risø reader TLDA-15 equipped with a single grain attachment with a 10mW Nd:YVO4 solid state diode-pumped laser emitting at 532 nm^83^. The luminescence emissions were detected through a Hoya U-340 filter. Single grains were measured on 9.6mm diameter aluminium discs containing 100 holes. D_e_ determinations used an identical SAR protocol to that of the single aliquot measurements except that stimulation was for only 1 s at 125^o^C. Single grain data analysis used OSL from the first 0.08s of stimulation as the signal integral and OSL from the last 0.48s as the background integral. Signal was again dominated by the rapidly decaying fast component (**Figure S8-B)**. Single grain D_e_ values from each aliquot were accepted following the criteria: i) an OSL signal measurable 3 sigma above background, ii) SAR growth curve within 1σ errors of regeneration points, iii) recycling values within ± 20 % of unity, and iv) the error on the test dose used within the SAR protocol was less than 20 %. As with the single aliquot measurements, the single grain SAR data found to be best fitted with a single saturating exponential curve for the youngest samples (Shfd20130-133) and a single saturating exponential + linear curve for the older samples (Shfd20134-137). Single grain SAR growth curves for most aliquot/samples showed good and continued growth (**Figure S8-D**). All samples included some grains which were in saturation the implications of which are discussed below in Section 3.5.5. Single grain dose recovery tests were undertaken on samples Shfd20133 and Shfd20136 with blue LED bleaching for 80s and laboratory doses of 78 Gy and 159 Gy respectively. These dose recovery ratios of 1.01±0.01 and 0.90±0.04 (1σ) respectively with OD values of 17% and 13% show the single grain quartz OSL approach applied here was appropriate.

**Figure S7: SA-OSL r**esults of different preheat temperatures in recovering a ~30 Gy beta radiation dose from sample Shfd20131. (a) Given to recovered dose ratio at different preheat temperatures. (b) recycling ratio (ratio between the first and last dose point) at the different preheat temperatures. Data points in both plots are the averages of three measurements performed for each pre-heat temperature and shading indicate conventional accepted limits for dose recovery and recycling. Uncertainties are calculated on the basis of three aliquots at each temperature. **
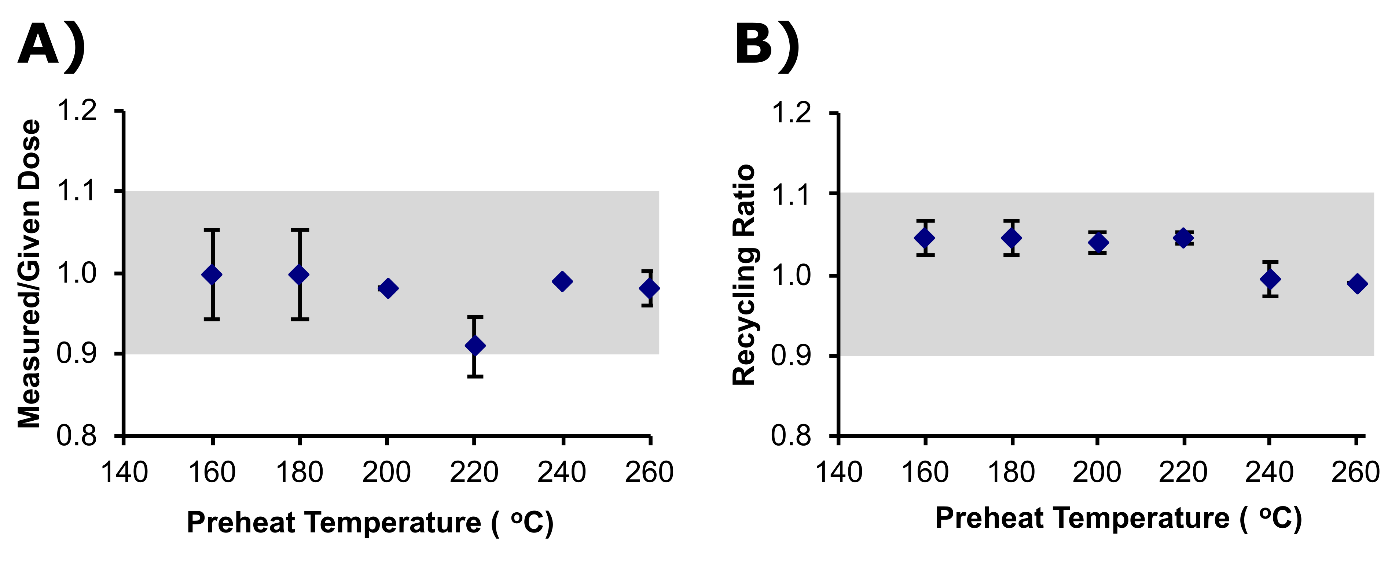
**

**Figure S8:** Examples of OSL data: (A) small aliquot measurements from samples Shfd20131 (ANY20-03) and Shfd20136 (ANY20-08) of rapid OSL decay of the naturally acquired signal indicating dominance of quartz fast component which has good bleaching and dose stability characteristics. Inset shows first 3 seconds of OSL stimulation; (B) single grain measurements from samples Shfd20130 (ANY20-02) and Shfd20136 (ANY20-08) also showing rapid OSL decay of the naturally acquired signal; (C) SAR growth curves from small aliquot measurements using the first 1.2s as signal and last 12s of signal measured as background. These show all samples have a good fit of regenerative points to a single saturating exponential for young samples and single saturating exponential + linear growth curve for old samples. (D) SAR growth curves from single grain measurements. This shows all samples had a good fit of regenerative points to a single saturating exponential for young samples and single saturating exponential + linear growth curve for old samples. The red line represents interpolation of the natural dose (D_e_). Errors on the SAR growth curves are based on counting statistics.


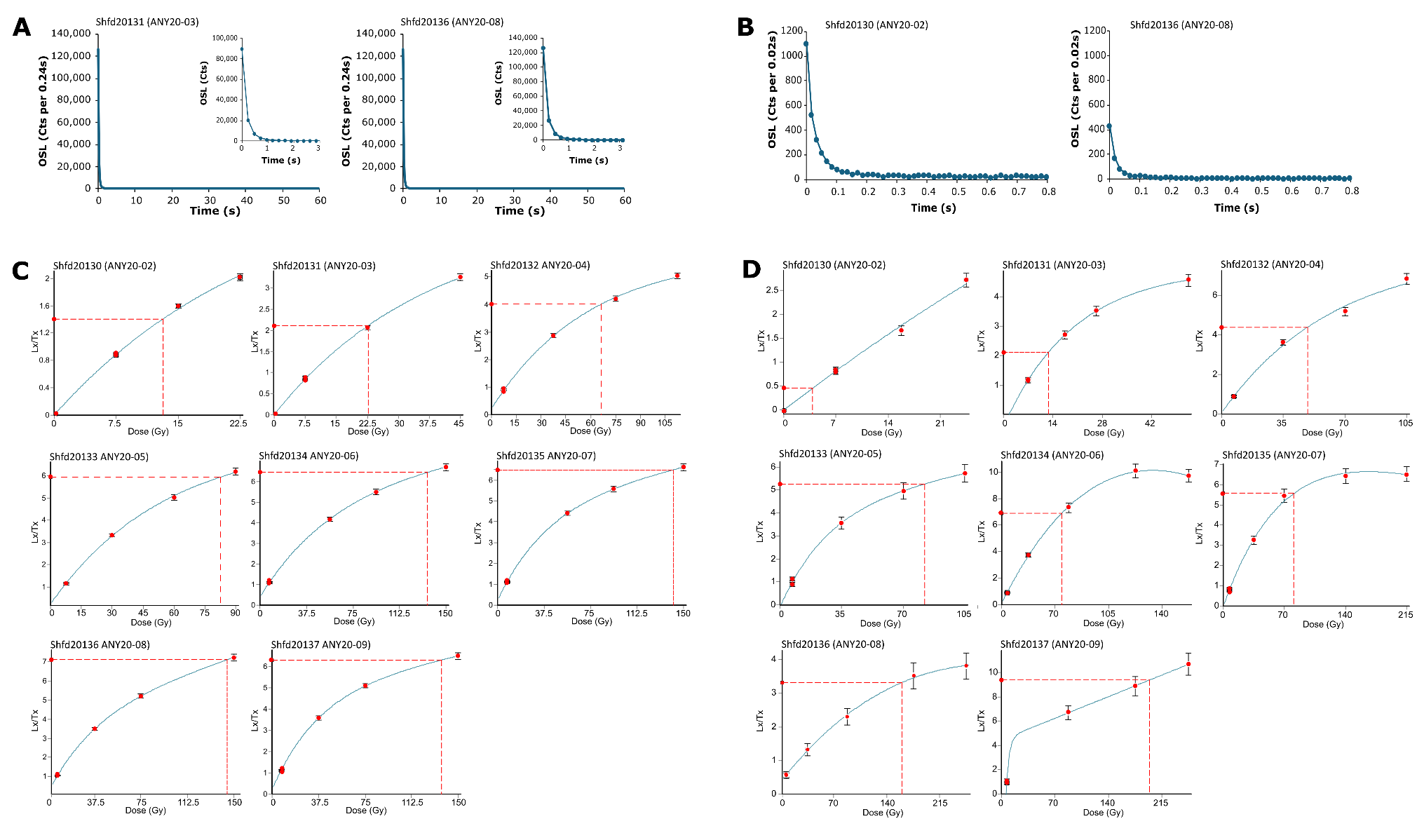


## 3.3 ESR analyses

Among all the sediment samples prepared for OSL, one sediment from Unit C (ANY20-03), another from the Interface C/D (ANY20-04) and three from Units D3 and Unit D1 (ANY20-09, ANY20-08 and ANY20-05) delivered sufficient quartz material for ESR dating. ESR dose evaluation following the Multiple Centre (MC) approach^84^ was carried out at the National Research Centre on Human Evolution (CENIEH, Burgos, Spain) using the standard Multiple Aliquot Additive Dose (MAAD) method. Each quartz sample was divided into 14 aliquots of approximatively ~70 to 200 mg (**Table S4**) including: 1 natural (non-irradiated), 1 UV-bleached and 12 laboratory-irradiated aliquots. Gamma irradiation was carried out using a calibrated Gammacell-1000 ^137^Cs gamma source (dose rate of about 6.4 Gy/min) and the following doses were given: 47, 112, 205, 419, 698, 1023, 1675, 2791, 4652, 7443, 13955 and 23258 Gy. One aliquot of each sample was UV-bleached using a Dr Hönle SOL-2 solar simulator for about 1500h to estimate the bleaching coefficient, which corresponds to the relative difference (in %) between the ESR intensity of the natural and bleached aliquots.

ESR measurements were performed at low temperature (~90 K) using an EMXmicro 6/1 Bruker X-band ESR spectrometer coupled to a standard rectangular ER 4104ST cavity and an ER4141VT Digital Temperature control system based on liquid nitrogen cooling. For constant experimental conditions, the temperature of the water circulating in the magnet is kept at 18 ºC by a water-cooled Thermo Scientific NESLAB ThermoFlex 3500 chiller while room temperature is set at 20 ºC by an air conditioning unit. Further details about the experimental setup and its performance may be found in^85,86^. According to the MC method^87^, the ESR dating signals associated with the Al and Ti centres were systematically measured. A first pre-screening of the Bété I quartz samples showed a well-defined Al signal intensity (**Figure S9-A**) from the peak-to-peak amplitude measurements between the top of the first peak (g = 2.0185) and the bottom of the 16th peak (g = 1.9928)^84^ and a relatively weak Ti signal whose intensity was hardly distinguishable from the background noise, as already pointed out by other relevant studies^86,88^. This is why, Al and Ti signals were acquired separately and acquisition parameters were optimised to increase the signal-to-noise (S/N) ratio for a better signal definition. In particular, we set up a new acquisition mode called ‘Twin windows’, which consists of working with two reduced acquisition windows on the targeted Ti-Li (window 2 ; **Figure S9-B**) Ti-H and Ti-mix (window 1 centred an Options C and D *sensu*^89^; **Figure S9-B**), in order to minimise sweeping time and increase the number of scans. Option C is made of a single contribution from the Ti-H signal, while the Option D is made of a mixture of Ti-Li and Ti-H signals (**Figure S9-B**). The latter will therefore be referred as Ti-mix throughout the manuscript.

For Al centre, ESR acquisition parameters were the following: 1 scan, 5 mW microwave power, 1024 points resolution, 100 kHz modulation frequency, 0.1 mT modulation amplitude, 40 ms conversion time, 10 ms time constant, and 40 s sweep time. Ti centres were measured as follows: 6 to 60 scans depending on the aliquot,5 mW microwave power, 200 points resolution, 100 kHz modulation frequency, 0.1 mT modulation amplitude, 60 ms conversion time, 10 ms time constant, and 12 s sweep time. The intensity of the Ti centres was measured following Duval and Guilarte^89^:

Each aliquot was measured 3 times after ~120° rotation in the cavity to consider the angular dependence of the signal due to evaluate variability of the ESR intensities and D_e_ values. All ESR intensities were corrected by their corresponding receiver gain value, temperature factor^90^, number of scans and aliquot mass. The noise was extracted and subtracted to the Ti ESR intensities following^88^. The mean ESR intensities derived from the repeated measurements were used for the final D_e_ determination.

Dose response curves (DRCs) for each signal were obtained from the mean ESR intensity values and associated 1 standard deviation derived from the repeated measurements. The experimental points were fitted using the Microcal OriginPro (OriginLab Corporation, Northampton, USA) using a Levenberg Marquardt algorithm by chi-square minimization. Several fitting functions were tested for D_e_ determination. For the Al centre, the exponential + linear (EXP+LIN) function was used[^65,66^](https://www.zotero.org/google-docs/?J5nvRI) with data weighted by 1/I² (I= ESR intensities). For the Ti centres, Ti-2 function^91,92^ with data weighting by 1/s² (s = experimental error) was used, and results were compared to those derived from the single saturating exponential (SSE) function^92^ (with data weighting by 1/I^2^). The final D_e_ errors include the uncertainty on the fitting and on the dose rate calibration (2.3%, 1σ)

**Figure S9:** examples of ESR signals obtained for samples ANY20-09 from single spectrum (A) and Twin windows (B) acquisition procedures. Window 1 is focused on Ti-H and Ti-mix signals (Options C and D), while Window 2 is centred on Ti-Li signal (Option E).
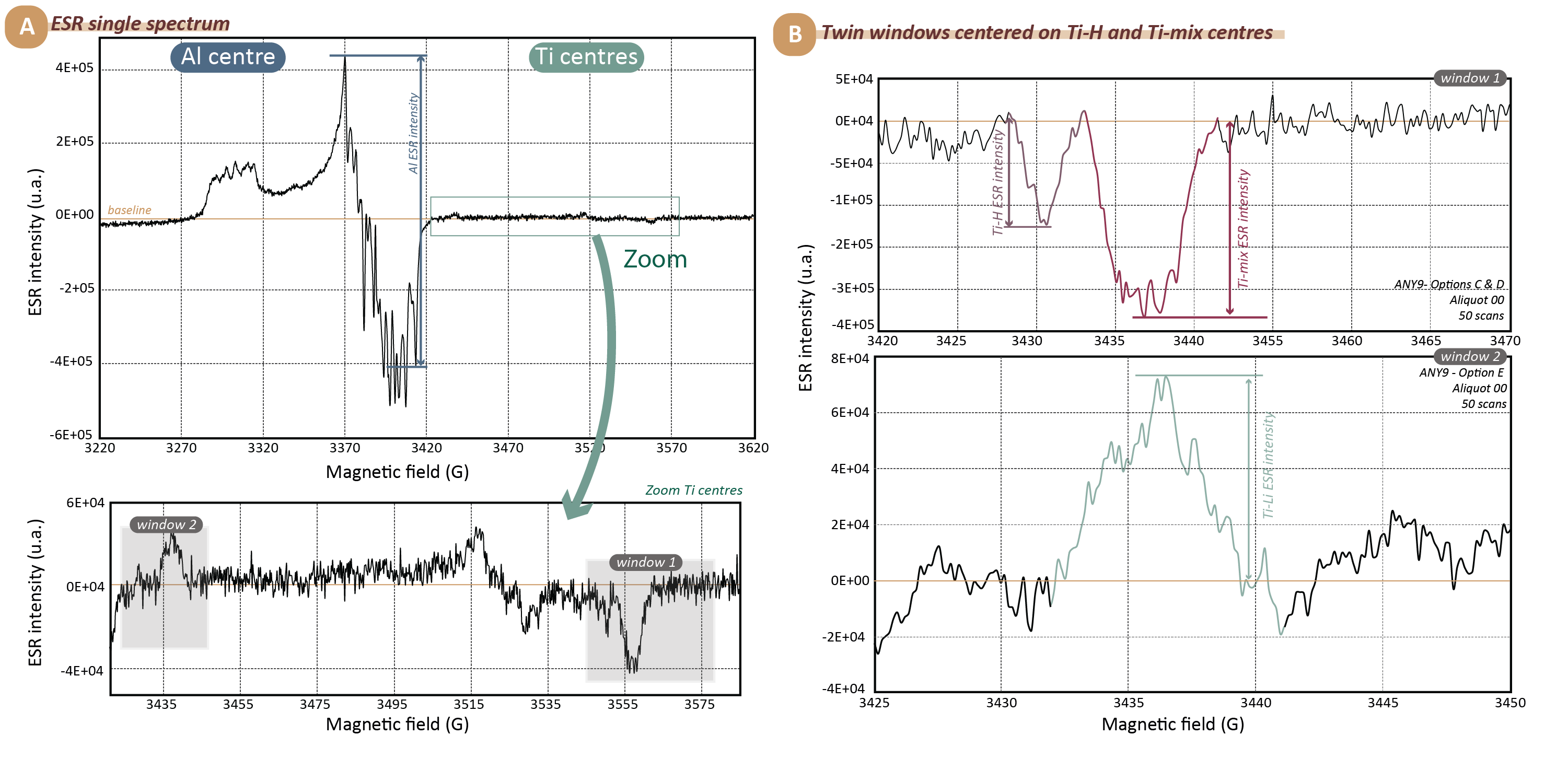


## 3.4 Dose rate evaluation

For ESR and OSL dating analyses, the total dose rate was derived from laboratory measurements. For each dated sample, ~3-5g of dry milled sediment were analysed in the SGS laboratories Ontario Canada by inductively coupled plasma mass spectrometry (ICP) to quantify the concentration of potassium (K), thorium (Th), uranium (U) and Rubidium (Rb). These values were used to calculate the external α, β and γ dose rate components using conversion factors from^93^. In addition, ~30 g of this same raw sediment, previously dried and powdered, were analysed by High Resolution Gamma Spectrometry (HRGS) using a HpGe detector in Archéosciences (Bordeaux, France) in order to identify possible disequilibrium in the ^238^U decay chain. Dose rate values were calculated for a grain size fraction of 180-212 µm (nominal sieve opening sizes) and an assumed thickness removed by HF etching of 10±5 µm^94^. Values were corrected with beta and alpha attenuation values for spherical grains^95,96^. An internal dose rate of 30 ± 10 μGy/a was considered, as commonly used in OSL and ESR dating application studies^97^. An alpha efficiency of 0.07 ± 0.01^98^ was used. For a matter of consistency between the ESR and luminescence dating procedures, the same long-term water content and depth values were employed for each shared sample. For both methods, ages and 1σ errors were calculated using DRAC^99^ (version 1.2) and the errors are 1σ, with cosmic dose rates derived from^100^ and including depth, altitude, and latitude corrections^101^.

Measured water contents of the sediment range from 3.2 to 12.5 % (% dry weight **Table S3**). These are likely underestimate of the long-term water content since burial. The sediment has most likely dried up over the last decades due to the original archaeological excavation carried out about 30 years ago, as well as the fact that our sampling occurred during the dry season. Our palaeoecological data shows the area was continuously humid over the Late Pleistocene with radiocarbon dating of the lowest terrace in the adjacent stream indicating landscape incision (leading to site drying) at the Pleistocene-Holocene transition. As a result, Holocene moisture contents could be expected to be lower than Pleistocene ones in which sediment show repeated flooding and alluvium deposition. Consequently, rather than rely on modern-day moisture values we instead assumed for the Holocene after the establishment of the current stream system in the catchment moisture contents of 8% for uppermost Unit C (based on the average unit value doubled to account for drying) and 12-13% for Unit D (based on the highest value measured rounded up). For earlier periods between initial burial and the Holocene, a higher moisture value of 20% was assumed based on the available geological evidence suggesting that frequent occurrence of flooding events. This value of 20 % is also based on WC (dry weight %) measured from sediments of the Units C and D sampled by EBA in November 2021 during the rainy season. For each sample initial ages based on modern-day moistures were used to estimate the duration of burial time at the lower Holocene and the higher Pleistocene moisture contents. These durations were used to weight a mean to derive the long-term average water contents for each sample. Given the likelihood of moisture fluctuations and acknowledging the uncertainties on the chronology, a conservatively large absolute uncertainty of ± 5% was estimated and applied to all the calculated long-term average moisture contents. (**Table S3**).

**Table S3: Moisture data used in the calculation of the average long-term water contents for the dose-rates for both ESR and OSL ages.** **The time-averaged moisture content (%) is the weighted arithmetic mean calculated between the assumed moisture post 10000 years (%) and the assumed moisture pre 10,000 years.**

| **ID field** | **OSL lab code** | **Depth (m)/Unit** | **Measured moisture**  **(% dry weight)** | **Assumed moisture post 10000 years (%)** | **Assumed moisture pre 10,000 years (%)** | **Time-averaged moisture content (%)** |
| --- | --- | --- | --- | --- | --- | --- |
| ANY20-02 | Shfd20130 | 0.85/C | 3.2 | 8 | 20 | 11 |
| ANY20-03 | Shfd20131 | 1.15/C | 4.6 | 8 | 20 | 15 |
| ANY20-04 | Shfd20132 | 0.90/C-D | 10.3 | 12 | 20 | 18 |
| ANY20-05 | Shfd20133 | 1.24/D | 11.8 | 12 | 20 | 18 |
| ANY20-06 | Shfd20134 | 1.90/D | 6.9 | 13 | 20 | 19 |
| ANY20-07 | Shfd20135 | 2.60/D | 5.4 | 13 | 20 | 19 |
| ANY20-08 | Shfd20136 | 3.60/D | 12.5 | 13 | 20 | 19 |
| ANY20-09 | Shfd20137 | 4.60/D | 12.3 | 13 | 20 | 19 |

## 3.5. Extended discussion of the chronological results

3.5.1 Al ESR D_e_ evaluation

The bleaching coefficient values vary from 24.3 % to 34.8%, indicating similar bleaching residuals for the samples (**Table S4**). The ESR data collected for the Al signal overall show a measurement repeatability within the usual standards^102^, with a variability of the ESR intensities of < 2.5%, including a D_e_ scatter less than 15% (<10 % for ANY20-08 and ANY20-09, >10% for ANY20-04 and ANY20-05). EXP+LIN function fitting results show regular (r² > 0.94) to high (r² > 0.99) goodness-of-fit depending on the sample considered.

**Table S4:** ESR data derived from the measurement of the Al centre. D_e_ are presented at 1-sigma. The ESR intensities repeatability is expressed as the relative standard deviation (coefficient of variation) of the average ESR intensities obtained from each set of ESR measurement. The D_e_ repeatability is evaluated via the variability of the D_e_ values obtained after each set of measurement, calculated as the relative standard deviation (coefficient of variation) from the mean value. Bl. Coeff (%) is the bleaching coefficient, which corresponds to the relative difference (in %) between the ESR intensity of the natural and bleached aliquots.

| **Al centre** | | | | | | | | | | | | | | |
| --- | --- | --- | --- | --- | --- | --- | --- | --- | --- | --- | --- | --- | --- | --- |
| **Sample ID** | **Measurement method** | | **Mean aliquot weight ± 1 s.d. (mg)** | | **Number of measurements** | | **Repeatability mean ESR intensities (%)** | **D_e_ repeatability (%)** | **Bl. Coef. (%)** | **EXP+LIN function**  **(W-1/I^2^)**  **D_max_=23 kGy** | | | |  |
|  | |  |  | |  | |  |  |  | D_e_ | Adj. r² | |  |  |
| ANY20-03 | | Single spectrum | | 100.4±0.04 | | 4 | 1.71 | 8.3 | 24.3 | 685±180 | | 0.967 |  |  |
| ANY20-04 | | Single spectrum | | 70.3±0.4 | | 4 | 1.05 | 12.7 | 25.4 | 810±257 | | 0.945 |  |  |
| ANY20-05 | | Single spectrum | | 200.5±0.3 | | 3 | 1.87 | 13.7 | 34.9 | 1203±158 | | 0.992 |  |  |
| ANY20-08 | | Single spectrum | | 199.2±4.1 | | 3 | 2.23 | 5.2 | 30.2 | 1438±263 | | 0.981 |  |  |
| ANY20-09 | | Single spectrum | | 200.5±0.3 | | 3 | 2.47 | 6.8 | 27.2 | 1267±248 | | 0.977 |  |  |

### 3.5.2. Ti ESR D_e_ evaluation

ESR intensities of the Ti centres were assessed through two options^103^: options C and D. Some previous studies^88,103–106^ have shown a relative consistency between Luminescence and ESR dating results based in either the Ti-H (Option C) or Ti-mix (Option D) signals for the last 300 ka while other studies (e.g. ^88,107^) have shown the consistency between OSL and Ti ESR ages is not obvious and can be sample dependant.

Fitting was carried out using a Ti-2 function (weighting by 1/s^2^) over the full dose range, and with a standard SSE function (weighting by 1/I^2^) as recommended by^92^ to fit the first part of the dose response curve, up to the maximum ESR intensities. To evaluate whether the maximum irradiation dose selected may have a significant impact on the calculated D_e_, the D_e_ were calculated with D_max_=4.5 kGy and D_max_=2.8 kGy. Numerical fitting results data are given in **Table S5**.

**Figure S10:** Dose response curves derived from the ESR signal of the Al centre measured in the quartz samples from Bété I. An EXP+LIN function was fitted through the experimental points up to D_max_ = 23 kGy. Vertical errors bars represent the associated 1 standard deviation of the mean intensities derived from the repeated measurements.


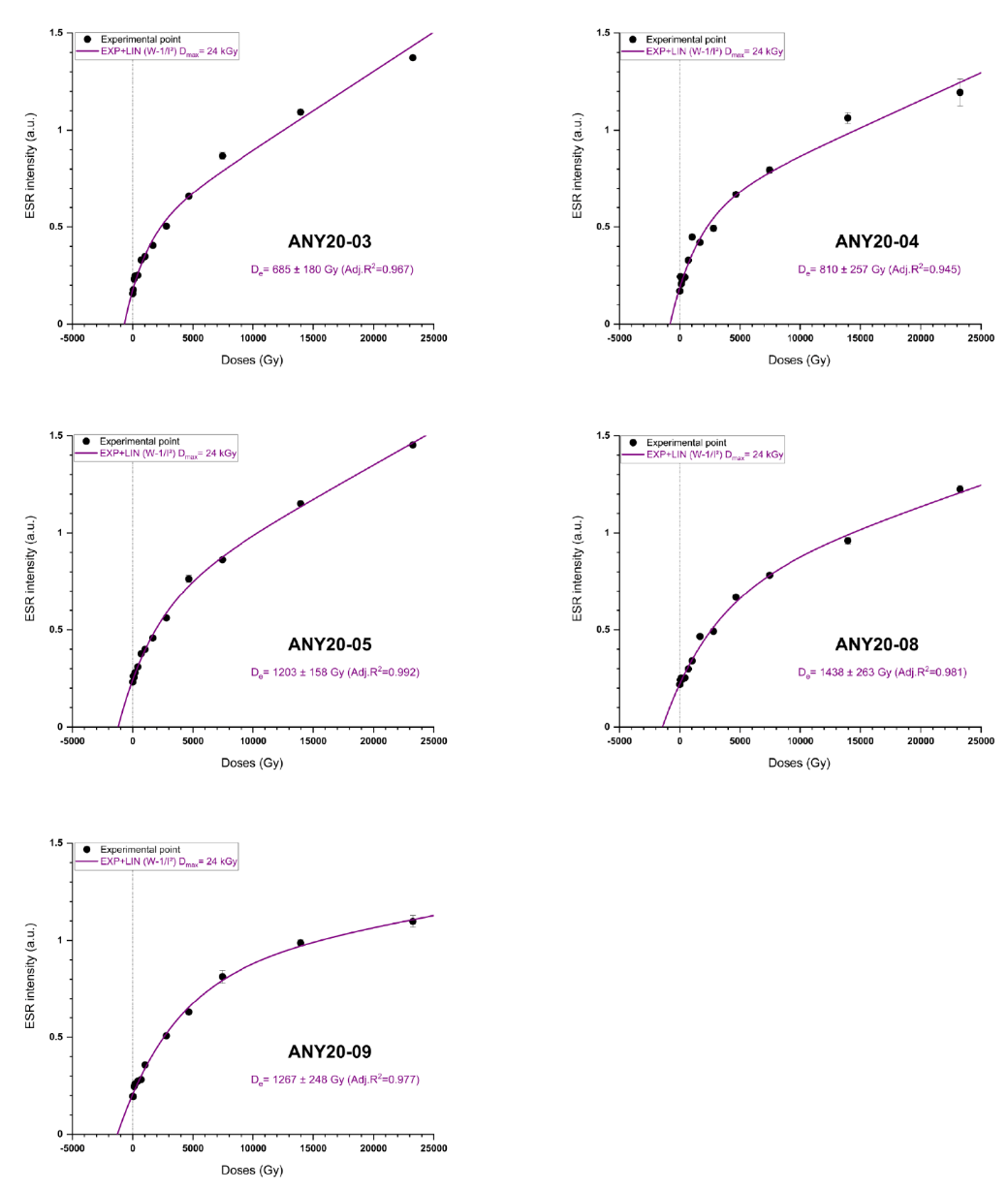


#### 3.5.2.1. Ti-H centre

Quality of the ESR data set

Repeated measurements of the Ti-H signal show the mean ESR intensity varying by 2.0 to 12.1 %, inducing a significant D_e_ scatter of 61.6 to 88.9 % depending on the sample considered (**Table S6**), which is beyond usual standards^102^. This low measurement repeatability is most likely due to the very weak signals measured in the samples^102^, as illustrated by the S/N ratio of 2 to 4 for the natural aliquots (**Table S6**).

Comparison of the fitting function

The D_e_ values resulting from the SSE function with D_max_ = 2.8 kGy and 4.5 kGy are consistent within error. In most cases (3/5), D_e_ results derived from D_max_ of 2.8 kGy are higher by about 5 to 9% than those from D_max_ of 4.5 kGy (**Table S5**). We consider the results derived from SSE fitting with D_max_ of 4.5 kGy as being more reliable because the goodness-of-fits for the five samples are, on average, higher (r^2^ between 0.84 and 0.96). For 4/5 samples, D_e_ obtained with Ti-2 function are 1σ-consistent with SSE (D_max_=4.5 kGy) results, ANY20-08 is the exception, with Ti-2 function returning an extremely low, and questionable, estimate of 47 ± 14 Gy, which is about 150 Gy lower than the SSE dose values. To sum up, despite the relatively low measurement repeatability and goodness-of-of fit achieved, fitting results appear to be relatively consistent and robust since the dose estimates are not significantly impacted by fitting conditions (i.e., functions, data weighting and D_max_).

Impact of the noise on the Ti-H D_e_ values

Following Ben Arous et al.^88,107^, we investigated the impact of high-frequency background noise in the Ti-H D_e_ evaluation. The difference observed between dose estimates derived from raw and noise-subtracted ESR intensities is inversely proportional to the magnitude of the D_e_ (**Table S5**). In other words, subtracting the background leads to a reduction of the D_e_ estimates by 27 to 50% for the uppermost three samples (ANY20-03, ANY20-04, ANY20-05), demonstrating the importance of such a correction for spectra showing very small S/N (2-3). In contrast, the D_e_ values remain within close range (10-36 Gy) for the oldest two samples ANY20-08 and ANY20-09, indicating that background noise has limited influence on the dose estimates. Moreover, fitting results based on uncorrected intensities show higher reliability, with higher goodness-of fit and smaller D_e_ errors (**Table S5**). Consequently, since noise subtraction does not bring any added value for these two samples, we used the D_e_ values of 240 ± 86 Gy and 253 ± 75 Gy for the Ti-H ESR age calculations of samples ANY20-08 and ANY20-09, respectively.

**Table S5**: Ti-H D_e_ values comparison with and without the noise subtraction. The data in italics are the value without noise subtraction.

| **Sample ID** | **ANY20-03** | | | **ANY20-04** | | | **ANY20-05** | | | **ANY20-08** | | | **ANY20-09** | | |
| --- | --- | --- | --- | --- | --- | --- | --- | --- | --- | --- | --- | --- | --- | --- | --- |
|  | **D_e_** | **± (%)** | **adj-r2** | **D_e_** | **± (%)** | **adj-r2** | **D_e_** | **± (%)** | **adj-r2** | **D_e_** | **± (%)** | **adj-r2** | **D_e_** | **± (%)** | **adj-r2** |
| D_e_ SSE D_max_ = 4.5 kGy | 47±15 | 31.9 | 0.934 | 52±22 | 42.3 | 0.888 | 107±25 | 23.4 | 0.961 | 204±93 | 45.6 | 0.840 | 243±103 | 42.4 | 0.869 |
|  | *100±28* | *28.0* | *0.943* | *105±31* | *29.5* | *0.941* | *147±31* | *21.1* | *0.965* | *240±86* | *35.8* | *0.894* | *253±75* | *29.6* | *0.930* |
| D_e_ SSE D_max_ = 2.8 kGy | 49±17 | 34.7 | 0.928 | 57±22 | 38.6 | 0.909 | 108±27 | 25.0 | 0.954 | 200±102 | 51.0 | 0.811 | 222±87 | 39.2 | 0.890 |
|  | *104±32* | *30.8* | *0.936* | *114±33* | *28.9* | *0.943* | *143±33* | *23.1* | *0.959* | *234±94* | *40.2* | *0.874* | *228±58* | *25.4* | *0.949* |

#### 3.5.2.2.Ti-mix signal

Quality of the ESR data set

Measurement repeatability is overall within usual standards for this signal^102^, with mean ESR intensities varying by 1.1 to 2.7%, resulting in a D_e_ scatter <15% for 4/5 samples, while ANY20-03 shows a much higher variability of 29 % (**Table S6**). This higher repeatability compared with the Ti-H signal is most likely related with the stronger ESR intensities of the Ti-mix signal^102^, as illustrated by the higher S/N values (4-10).

Comparison of the fitting function

The SSE D_e_ values derived from fittings carried out with D_max_ = 2.8 kGy and 4.5 kGy are consistent within error for 4/5 samples. However, the goodness-of-fit is on average higher with SSE fitting using D_max_ at 4.5 kGy (adj. r^2^ = 0.947-0.990) and even higher for the Ti-2 function (adj. r^2^ = 0.959-0.993). Note that for 3/5 samples, D_e_ values determined with the three fitting functions are 1σ consistent, indicating that fitting conditions do not significant bias dose estimates.

### 3.5.3. Comparison of the D_e_

The comparison of the D_e_ values derived from the Al and Ti-mix signals shows that only 2 samples return consistent results (the lowermost ANY20-08 and ANY20-09). No sample show an overlap of the Al and Ti-H dose estimates. The Al centre systematically provides higher D_e_ values by 50 to 70% than Ti centre for all the samples, while the Ti-H centre systematically provides the smallest dose estimates. Following the basic principles of the Multiple Centres approach and the recommendation in^11^, this suggests that the Al and Ti-mix signals have been incompletely reset during transport or redeposition of the sediment. In other words, the Ti-H signal, which has the fastest bleaching kinetics^84,108^, is more likely to be fully reset than any other ESR signals. Consequently, it may be regarded as yielding the closest estimation of the true burial dose, provided there is no evidence indicating that the Ti-H D_e_ value may be underestimated, which is the case here. Indeed, importantly, in the present study the magnitude of the dose estimates obtained for these 5 samples (50-250 Gy) falls within the range usually regarded as suitable (<300 Gy; e.g., ^104,109^) for the use of the Ti-H signal for dose determination. Consequently, the Ti-H centre was used for the final dose evaluation and ESR age calculations.

### 3.5.4. Dose rate evaluation

Radioelement concentrations for dose rate evaluation are given in **Table S7**. They show an overall relative homogeneity within each Unit and increase with depth. The two samples from Unit C contain 0.1 % of K, 1.3 ppm U, 6-7 ppm Th. The five others samples from Unit D contain 0.2 % of potassium, between 1.5-1.9 ppm uranium and 11-12 ppm thorium. Additional HGRS analyses were performed to evaluate a possible radioactive disequilibrium in the ^238^U decay chain (**Table S7**). U content determination was based on (i) the beginning (^238^U derived from the activities of ^234^Th, ^234^Pa and ^235^U), (ii) the middle (^226^Ra deduced from the measured activity of ^214^Pb and ^214^Bi) and (iii) the end (^210^Pb) of the ^238^U decay chain. ^210^Pb/^238^U and ^226^Ra/^238^U activity ratios were also calculated. ^210^Pb/^238^U are close to unity but between 0.76 and 0.89 for ^226^Ra/^238^U, indicating a potential slight disequilibrium in the middle of the chain, possibly resulting from Ra loss, which is a fairly mobile element. Given the short half-life of this element (1599 years), this Ra-loss may possibly be a very recent overprint associated with some late Holocene changes in the geochemical conditions of the surrounding environment, and is unlikely to be representative of the full burial history of the sample. Regardless, assuming that this disequilibrium has prevailed throughout the burial history has no significant impact on the dose rate: the values assuming equilibrium (from 1219±46 to 1169±44 μGy/a) and constant post-Ra disequilibrium (from 1171±42 to 1136±41 μGy/a) slightly differ by only 2-4 %, resulting in a limited impact on the calculated OSL and ESR age. HGRS and ICP analytical results are overall consistent. Final ages parameters calculations are based on the ICP-MS data for U and Th (**Table S6 and S7**), HRGS K content, the latter being close to the detection limit (~0.1 %) of ICP analysis.

**Table S6:** Final ESR data derived from measuring the Ti-H and Ti-mix signals. *Average S/N value calculated from the repeated measurement of he natural aliquot. The ESR intensities repeatability is expressed as the relative standard deviation (coefficient of variation) of the average ESR intensities obtained from each set of ESR measurement. The D_e_ repeatability is evaluated via the variability of the D_e_ values obtained after each set of measurement, and calculated as the relative standard deviation (coefficient of variation) from the mean value. Mean aliquot weights are reported in Table S4.

| **Ti-H signal (Option C)** | | | | | | | | | | |
| --- | --- | --- | --- | --- | --- | --- | --- | --- | --- | --- |
| **Sample** | **Measurement method** | **Number of repeated measurements** | **Repeatability ESR intensities (%)** | **D_e_ repeatability (%)** | **S/N*** | **Ti-2 function (W-1/s^2^)**  **D_max_=23 kGy** | | | **SSE function (W-1/I^2^)**  **D_max_ = 4.5 kGy** | |
|  |  |  |  |  |  | D_e_ | Adj. r² | D_e_ | | Adj. r² |
| ANY20-03 | Twin windows | 3 | 12.1 | 88.9 | 2 | 52±19 | 0.951 | 47±15 | | 0.934 |
| ANY20-04 | Twin windows | 3 | 8.7 | 69.0 | 2 | 60±33 | 0.819 | 52±22 | | 0.888 |
| ANY20-05 | Twin windows | 3 | 2.0 | 70.9 | 3 | 117±38 | 0.913 | 107±25 | | 0.961 |
| ANY20-08 | Twin windows | 3 | 6.6 | 67.9 | 3 | 47±14 | 0.928 | 240±86 | | 0.894 |
| ANY20-09 | Twin windows | 3 | 3.7 | 61.6 | 4 | 264±73 | 0.890 | 253±75 | | 0.930 |
| **Ti-mix signal (Option D)** | | | | | | | | | | |
| **Sample** |  | **Number of repeated measurements** | **Repeatability ESR intensities (%)** | **D_e_ repeatability (%)** | **S/N*** | **Ti-2 function (W-1/s^2^)**  **D_max_=23 kGy** | | **SSE function (W-1/I^2^)**  **D_max_ = 4.5 kGy** | | |
|  |  |  |  |  |  | D_e_ | Adj. r² | D_e_ | | Adj. r² |
| ANY20-03 | Twin windows | 3 | 2.1 | 29.0 | 4 | 481±39 | 0.987 | 542±74 | | 0.981 |
| ANY20-04 | Twin windows | 3 | 2.2 | 11.9 | 4 | 501±84 | 0.959 | 562±129 | | 0.947 |
| ANY20-05 | Twin windows | 3 | 1.1 | 9.0 | 7 | 675±43 | 0.993 | 634±62 | | 0.990 |
| ANY20-08 | Twin windows | 3 | 1.1 | 1.7 | 8 | 1150±105 | 0.993 | 1501±246 | | 0.980 |
| ANY20-09 | Twin windows | 3 | 2.7 | 4.4 | 10 | 1166±118 | 0.982 | 1116±153 | | 0.983 |

**Figure S11:** Dose Response Curves (DRCs) derived from the ESR signal of the Ti centres measured in the quartz samples from Bété I. Fitting was performed as follows: (see text for further information): a) with the Ti-2 function (data weighting by 1/s^2^), b) with the single saturating exponential (SSE) function (data weighting by 1/I^2^) with D_max_ = 4.5 kGy and c) D_max_ = 2.8 kGy. Vertical errors bars represent 1 standard deviation derived from the repeated measurements. DRCs display ESR intensities after noise subtraction.


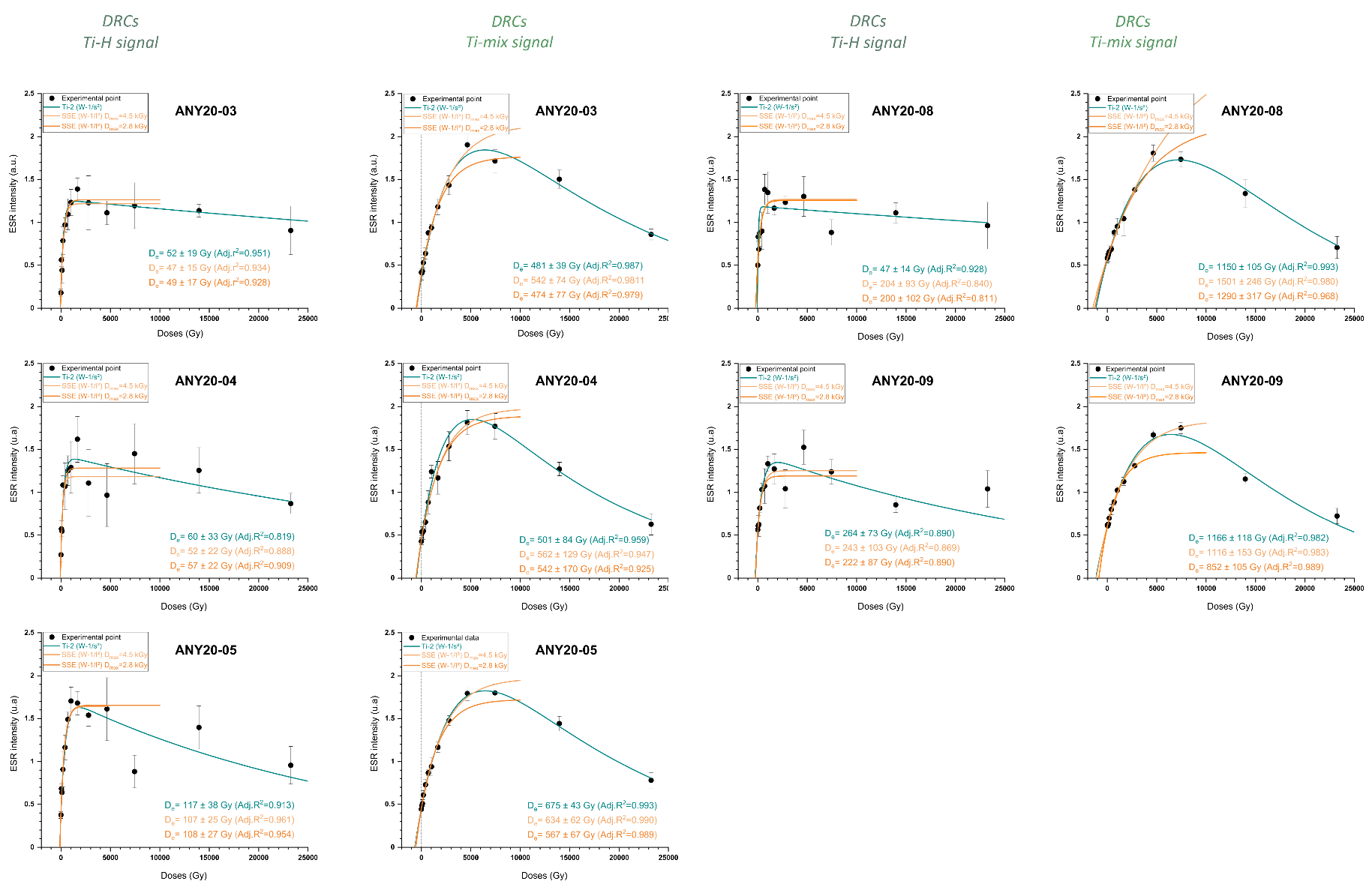


**Table S7:** Comparison of the radioelement contents measured by ICP-MS and HRGS on the 8 sediment samples dated in this study. a: Corresponds to the pre-^226^Ra and is deduced mainly from the ^234^Th and ^235^U peaks; b: Corresponds to the post-^226^Ra, calculated from the ^214^Pb and ^214^Bi peaks; c: Corresponds to the bottom of the chain, calculated from the ^210^Pb peak. Samples with (*) were dated by both OSL and ESR methods. ^210^Pb/^238^U and ^226^Ra/^238^U are the ratios of the specific activity (Bq/kg).

|  | | **ICP-MS measurements** | | | | **High Resolution Gamma Spectrometry (HRGS)** | | | | | | |
| --- | --- | --- | --- | --- | --- | --- | --- | --- | --- | --- | --- | --- |
| ID Field | Unit | U (ppm) | Th (ppm) | K (%) | Rb (%) | U-238 head^a^ | U-238 middle^b^ | U-238 end^c^ | Th-232 | K-40 (%) | ^210^Pb/^238^U | ^226^Ra/^238^U |
|  |  |  |  |  |  | (ppm) | | | |  |  | |
| ANY20-02 | C | 1.20±0.12 | 6.3±0.63 | 0.1± 0.01 | 6.2±0.62 | 1.24±0.09 | 0.99±0.02 | 1.30±0.11 | 6.4±0.07 | 0.07±0.01 | 1.04 | 0.80 |
| ANY20-03* | C | 1.28±0.13 | 6.8±0.70 | 0.1±0.01 | 7.1±0.71 | 1.46±0.08 | 1.11±0.02 | 1.50±0.10 | 6.9±0.07 | 0.05±0.01 | 1.03 | 0.76 |
| ANY20-04* | C/D | 1.59±0.16 | 10.0±1.0 | 0.2±0.02 | 10.0±1.0 | 1.76±0.11 | 1.51±0.03 | 2.02±0.13 | 9.9±0.11 | 0.09±0.01 | 1.15 | 0.86 |
| ANY20-05* | D1 | 1.93±0.20 | 12.1±1.2 | 0.2±0.02 | 12.0±1.2 | 1.89±0.12 | 1.60±0.03 | 2.13±0.14 | 11.45±0.12 | 0.11±0.01 | 1.13 | 0.84 |
| ANY20-06 | D2 | 1.57±0.16 | 10.9±1.1 | 0.2±0.02 | 10.2±1.0 | 1.61±0.12 | 1.37±0.03 | 1.65±0.14 | 10.52±0.12 | 0.13±0.01 | 1.02 | 0.85 |
| ANY20-07 | D2 | 1.59±0.16 | 10.6±1.1 | 0.2±0.02 | 9.6±1.0 | 1.60±0.11 | 1.34±0.03 | 1.78±0.13 | 10.04±0.11 | 0.08±0.01 | 1.11 | 0.84 |
| ANY20-08* | D3 | 1.55±0.16 | 10.6±1.1 | 0.2±0.02 | 9.3±0.93 | 1.67±0.11 | 1.43±0.03 | 1.60±0.13 | 10.48±0.11 | 0.10±0.01 | 0.96 | 0.86 |
| ANY20-09* | D3 | 1.49±0.15 | 10.2±1.0 | 0.2±0.02 | 8.3±0.83 | 1.50±0.11 | 1.34±0.03 | 1.60±0.12 | 10.28±0.11 | 0.11±0.01 | 1.07 | 0.89 |

### 3.5.5 OSL equivalent dose evaluation

*Single Aliquot (SA-OSL) data*

Analysis of the replicates aliquots from the single aliquot (SA) measurements from each sample showed samples ANY20-03 and ANY20-04 (Shfd20131-132) and samples ANY20-06 and ANY20-09 (Shfd20134-137) had unimodal and unskewed D_e_ distributions (some with a few aliquots with very low D_e_ values) and low overdispersion (OD; OD < 20%) once outliers were removed **Figure S12**; **Table S8**). Outliers were defined where D_e_ values fell outside the inter-quartile range. The latter was calculated as per ^110,111^ using the difference of the 25^th^ from the 75^th^ percentile of the full D_e_ dataset multiplied by 1.5 to give the fourth spread. This fourth spread value was added to or subtracted from the median D_e_ to give upper and lower interquartile boundaries. These samples are assumed to have been well bleached prior to burial with some very limited post-depositional disturbance or beta heterogeneity^73^. On this basis, once outliers were excluded, D_e_ values for age calculation purposes were extracted using the Central Age Model (CAM)^112^. The SA data for samples ANY20-02 (Shfd20130) and ANY20-05 (Shfd20133), whilst also unimodal, had high OD values (>30% after outliers removed) and broader D_e_ distributions with the latter sample also containing saturated aliquots (**Figure S12**). These samples are assumed not to have been fully bleached prior to burial. Therefore, D_e_ values for age calculation purposes were extracted from these two samples using the Minimum Age Model (MAM^112^) with a sigma-b of 0.2 based on the OD from dose recovery tests. Application of the MAM to exclude aliquots with higher D_e_ values meant that the exclusion of aliquots with D_e_ values in saturation in this sample also did not impact on the calculated age. SA data for samples ANY20-07 (Shfd20135) and ANY20-09 (Shfd20137) both had 4 saturated aliquots out of the 24 aliquots initially measured. As discussed above, the D_e_ distributions for these samples did not warrant application of the Finite Mixture Model (FMM^113^) as they are normally distributed. Therefore, the CAM was applied for age calculation purposes. However, as the CAM D_e_ values are below but close to the saturation value (as defined by 2D_0_), exclusion of saturated aliquots could lead to the computed average D_e_ being biased towards lower D_e_ values. The ages for these two samples may therefore be minimums. Final OSL ages are provided in **Table S8** with 1 sigma uncertainties.

*Single grain (SG-OSL) data*

Analysis of the replicates from the single grain (SG) measurements showed all samples had broad unimodal D_e_ distributions and OD values between 48%-12% once outliers (as defined above) were removed^114^ (Figure **S13**; **Table S8)**. It was observed that the D_e_ distributions of most samples also contained a few grains with very low D_e_ values. The presence of a similar numbers of low D_e_ values were also observed in the two dose recovery tests undertaken thereby ruling out that the low D_e_ values in the samples with burial age D_e_s are due to post-depositional disturbance or beta heterogeneity^73^. Instead it is taken to indicate that a limited number of grains, whilst meeting the quality assurance criteria outline in section 3.2, had other (uninvestigated) poor behavioural characteristics resulting in them not reflecting the true burial dose. Dose recovery experiments also encountered many saturated grains indicating that some grain in these samples had much lower than average saturation limits. Saturation is interpreted therefore as being due to grains being buried for sufficient antiquity to reach their saturation limits rather than indicating grains which had not be reset at all at burial and only buried for a shorter time. Due to the low D_e_ grains MAM was not applied to the SG data for age calculation purposes. Instead, based on the lack of evidence for partial bleaching, after outlier removal^110,111^, CAM was applied to all samples for age calculation purposes.

**Figure S12:** Abanico plots of D_e_ replicate small Single Aliquot (SA) OSL data centered on the Central Age Model D_e_ with the grey bar showing the selected D_e_ used for age calculation purposes. Open circles indicate aliquots considered as outliers^110^ (see text for details). Overdispersion (OD) as shown is prior to outlier removal and grey bar shows the 2 standard error estimate on the Central Age D_e_.


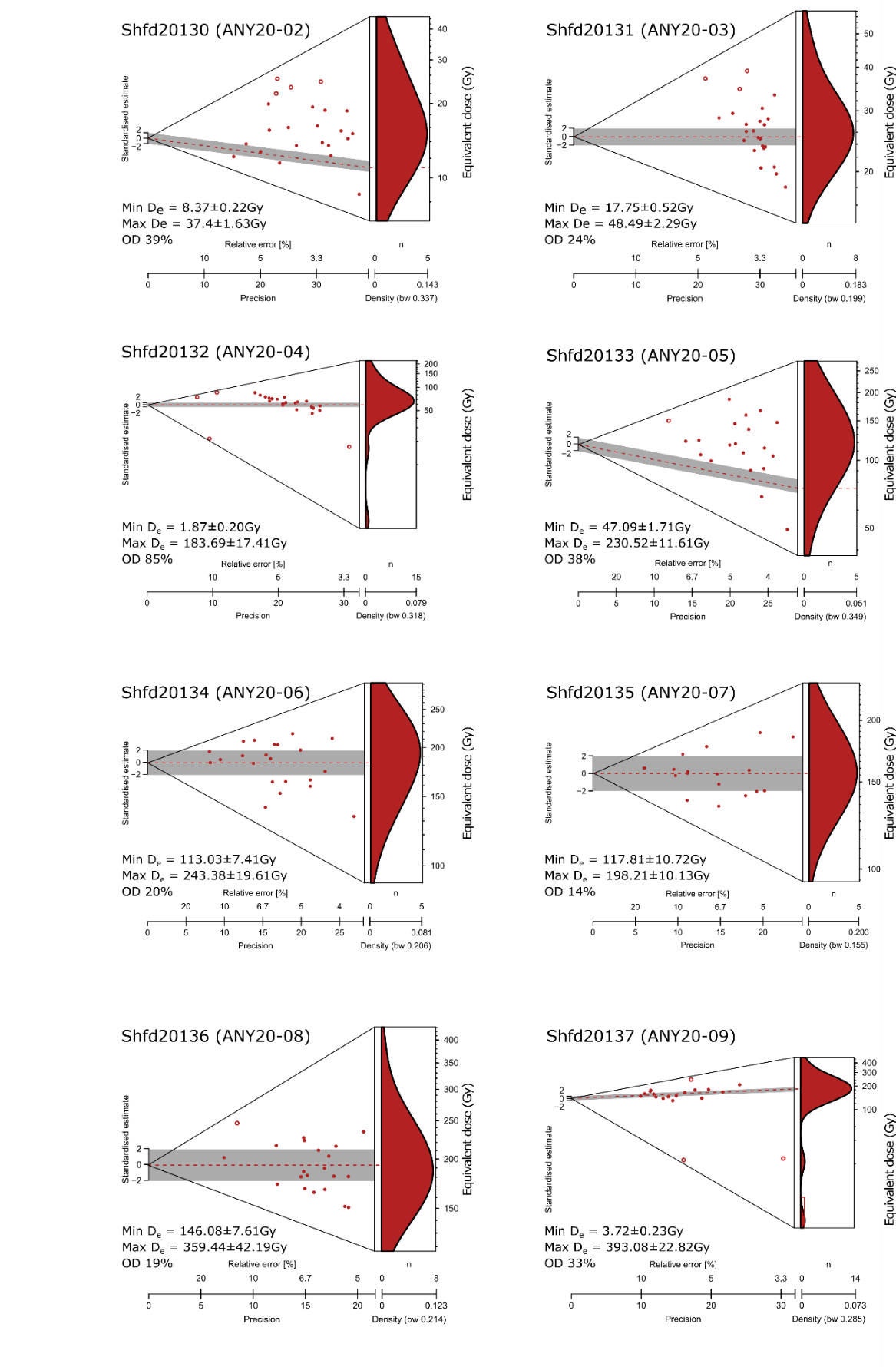


**Figure S13:** Abanico plots of D_e_ replicate Single Grain (SG) OSL data centered on the Central Age Model D_e_ also used for age calculation purposes. Open circles indicate aliquots considered as outliers^110^ (see text for details). Overdispersion (OD) as shown is prior to outlier removal and grey bar shows the 2 standard error estimate on the Central Age D_e._.


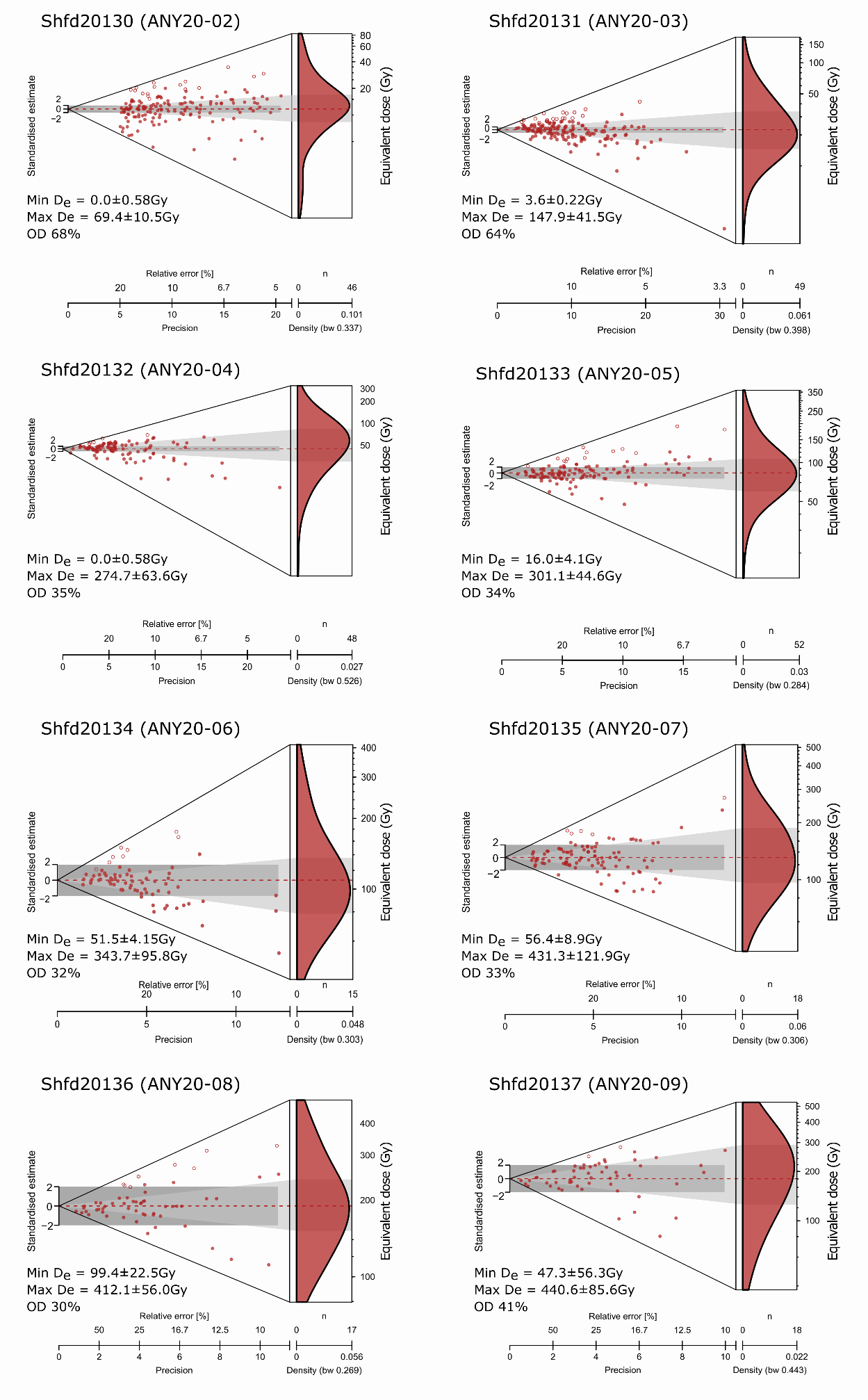


- - 1. Comparison between SA-OSL, SG-OSL and ESR chronologies for Bété I sequence

SA-OSL, SG-OSL and Multi-grain (MG)-MC-ESR age estimates presented in **Table S8** and **Table S9** were calculated using the D_e_ values derived from SA and SG measurements and from both the Al and Ti-H signals. Gamma, beta and alpha dose rates derived from ICP-MS and HRGS data. The calculated dose rates range from 917±48 and 1385±53 μGy/ka.

The Al ESR ages range from 684±218 ka to 1242±232 ka and are systematically older than the corresponding Ti-mix ESR ages (from 458±30 ka to 1033±111 ka) and older than the Ti-H ESR ages, from 44±19 ka to 227±68 ka. The ages obtained from the more rapidly and completely bleached Ti-H signal are considered to yield the closest estimate of the true burial age for the deposits^108^. Consequently, and in accordance with previous works focused on the last 300 ka^88,104,107,109^, we consider the Ti-H signal to provide the most reliable estimates to constrain the chronology of the deposits at Bété I.

SA-OSL results show coherence with depth, with ages bracketing the Unit C between 27±3 and 12±2 ka. The transition from Unit C and D appeared to be at 55±4 ka and the ages are bracketing the Unit D deposits between 54±8 and 165±11 ka. Two of the SA-OSL ages (134±7 ka from sample ANY20-07 and 165±11 ka from sample ANY20-09) are minimum ages and explained by the presence of saturated aliquots. The comparisons between the multi-grain (MG) SA-OSL and Ti-H ESR ages of samples from the base and top of the Unit D (ANY20-09, ANY20-08 and ANY20-05) show results that are systematically in agreement at 1σ (>165 ± 11 ka vs 227 ± 68 ka; 162 ± 9 ka vs 207 ± 75 ka; 54 ± 8 ka vs 77 ± 18 ka). We nevertheless acknowledge the relatively large errors associated to the Ti-H ESR estimates (between 23 and 36%), which mostly result from the relatively low goodness-of-fit of the ESR DRCs (section 3.5.2.1). This good consistency between the two semi-independent methods is significant as the bleaching times for the ESR signal measurements are much longer than for OSL^108^, which could have led to significant age over-estimated compared to the OSL if bleaching of the sediments prior to burial was an issue. On the contrary, the SA-OSL and ESR ages at the top of the sequence (ANY20-04 and ANY20-03) differ and are not consistent at a 1σ confidence level. The older ESR results at the top of the sequence may be equally resulting from an incomplete reset of the Ti-H signal during transport, and/or simply illustrating the limits of the ESR method and reflecting the difficulty to get reliable ESR dating results for sediments with very low D_e_ values (<100 Gy), as shown previously for other south African samples of the same time range (<50 ka)^88,107^.

In order to refine the SA-OSL chronology, and especially the minimum age estimates obtained for some of the samples, we performed additionally SG-OSL. Overall, the SG D_e_ values are slightly, but systematically, younger than their single aliquot counterparts for all samples reflecting the overall lack of grain averaging and better exclusion of saturated grains compared to the small SA data (**Figures S12-13, Table S8**). Comparison of SA and SG-OSL data show that these are within 1σ errors for 4 of the 8 samples (ANY20-02, ANY20-05, ANY20-08 and ANY20-09. In contrast, the SG-OSL age for ANY20-06 is ~70 ka younger than the SA-OSL estimates, despite having low OD values for both types of measurement. The SG-OSL chronology on its own appears robust: the overall antiquity of the human presence is similar for both levels of measurements as show by the good consistency at 1σ level between SA-OSL and SG-OSL ages of the sample ANY20-08 (respectively 162±9 ka and 146±9 ka). The age for sample ANY20-03 reduces from 27±3 ka (SA) to 20±1 ka (SG) and as this is the same unit as ANY20-02 (12±1 ka) the SG age is preferred. ANY20-04 reduces from 55±4 ka (SA) to 35±3 ka (SG). As this sample is from a transitional unit between C and D, an age much younger than the upper Unit D age (54±8 ka) makes better stratigraphical sense so the SG age is preferred. The comparatively older age estimates obtained from SA measurements most likely result from grain averaging effects (e.g.^115^).

To sum up, the SG-OSL chronology is regarded as providing more accurate estimates of the true burial age of the deposits, as it allows: (i) to evaluate any potential bias resulting from grain averaging effects, (ii) isolate saturated grains and obtain finite age results for the lowermost samples; and (iii) it resolves an age reversal in the SA-based chronostratigraphy, with the SG ages for Unit D increasing with depth.

### 3.5.7. Sensitivity test with the variation of water content

We performed age sensitivity tests by varying the moisture content from present-day values to 20% (% dry weight) and according to the time-averaged water content provided in **Table S3**. The results are displayed in **Figure S14** and **Tables S10 and S11**. SA-OSL, SG-OSL and Ti-H ESR ages were recalculated respectively with 10, 15 and 20% of water content show ages between ~2% and 14% higher than ages calculated with present-day water content (**Tables S10** and **S11**). However, the ages are all consistent at 1σ, implying that present-day water content or time-averaged water content do not have a significant impact on the SA/SG-OSL and ESR ages of the Bété I sequence. As the site was originally excavated over 30 years ago and current sampling took place during the dry season, measured water content probably underestimate the long-term water content. According to palaeo-ecological data presented in this work and our field observations, the area has been consistently wet (tropical forest along the Middle to Upper Pleistocene). Consequently, we consider that final OSL and ESR calculations based on time-averaged water content are the most reliable estimate of the true age of the deposits from Bété I.

**Table S8:** SA and SG**-**OSL age estimates and dose rate components. For single aliquot measurements n represents the number of 24 aliquots measured which met the quality control criteria with the number after outlier removal shown in parenthesis. For single grain measurements n represents the number of grains which met the quality control criteria with the number after outlier removal shown in parenthesis. 800 single grains were measured for sample Shf20131, 900 for samples Shfd20134 and Shfd20137 and 1000 for samples Shf20130, Shfd20132, Shfd20135 and Shfd20136. n (saturated) represents the number of small aliquots/grains excluded as the SAR growth curve failed to meet the naturally acquired signal or was in the low gradient part of the growth curve. These small aliquots/grains are interpreted as exceeding the limit of the technique in antiquity and/or those where no resetting prior to burial took place. n (zero dose) represents small aliquots/grains where the D_e_ is within error of zero which are interpreted as indicating recent exhumation and/or burial by pedoturbation processes or site disturbance. Overdispersion (OD) is the “spread” in the D_e_ distribution that remains after all the measurement errors specific to each aliquot have been taken into account^116^. OD values in parenthesis being the overdispersion after outliers were removed. Equivalent doses (D_e_) are shown as calculated by the Central Age Model (CAM), Minimum Age Model (MAM) and the dominant component from Finite Mixture Model (FMM) with the latter two based on all aliquots (no outliers excluded) and using an assumed σ_b_ value of 0.2. D_e_ used in final age calculation (as discussed in text) is shown in bold. Errors are displayed at 1 sigma. *: paired ESR samples. Minimum ages are in italic.

| **SAMPLE** | **ANY20-02 (Shfd20130)** | | **ANY20-03* (Shfd20131)** | | **ANY20-04* (Shfd20132)** | | **ANY20-05* (Shfd20133)** | | **ANY20-06 (Shfd20134)** | | **ANY20-07**  **(Shfd20135)** | | **ANY20-08* (Shfd20136)** | | **ANY20-09* (Shfd20137)** | |  |
| --- | --- | --- | --- | --- | --- | --- | --- | --- | --- | --- | --- | --- | --- | --- | --- | --- | --- |
| Depth (m) | 0.85±0.1 | | 1.15±0.12 | | 0.9±0.09 | | 1.24±0.12 | | 1.9±0.2 | | 2.6±0.3 | | 3.6±0.36 | | 4.6±0.46 | |  |
| Water (MW) content (dry weight %) | 11±5 | | 15±5 | | 18±5 | | 18±5 | | 19±5 | | 19±5 | | 19±5 | | 19±5 | |  |
| **SINGLE ALIQUOT** | | | | | | | | | | | | | | | | |  |
| n | 23 (19) | | 24 (21) | | 24 (19) | | 20 (18) | | 22 (22) | | 17 (17) | | 21 (20) | | 19 (16) | |  |
| n (saturated) | 0 | | 0 | | 0 | | 4 | | 1 | | 4 | | 2 | | 4 | |  |
| n (zero dose) | 0 | | 0 | | 0 | | 0 | | 0 | | 0 | | 0 | | 0 | |  |
| OD (%) | 39 (28) | | 24 (17) | | 85 (23) | | 38 (33) | | 20 (20) | | 14 (14) | | 19 (15) | | 102 (21) | |  |
| MAM D_e_ (Gy) | **10.98±1.5** | | 24.96±2.4 | | 2.46±0.71 | | **75.0±10.8** | | 182.8±15.2 | | 156.3±12.3 | | 189.8±16.7 | | 3.72±0.78 | |  |
| CAM D_e_ (Gy) | 14.46±0.94 | | **25.19±0.95** | | **65.5±3.5** | | 109.8±8.8 | | **182.8± 8.2** | | **156.1±6.0** | | **187.8±7.1** | | **184.0±10.3** | |  |
| FMM D_e_ (Gy) | 13.54±0.84 | | 25.43±1.4 | | 64.98±3.5 | | 108±10.3 | | 186.3±15.7 | | 156.34±8.1 | | 192.9±18.2 | | 192.9±9.9 | |  |
| Internal dose rate (μGy/a) | 30±1 | | 30±1 | | 30±1 | | 30±1 | | 30±1 | | 30±1 | | 30±1 | | 30±1 | |  |
| Alpha dose rate (μGy/a) | 16±2 | | 16±2 | | 22±2 | | 26±3 | | 23±2 | | 22±2 | | 22±2 | | 21±2 | |  |
| Beta dose rate (μGy/a) | 297±25 | | 290±25 | | 394±27 | | 478±33 | | 428±28 | | 395±27 | | 403±27 | | 395±27 | |  |
| Gamma dose rate (μGy/a) | 403±36 | | 411±36 | | 563±31 | | 682±37 | | 600±32 | | 580±31 | | 580±31 | | 561±30 | |  |
| Cosmic dose rate (μGy/a) | 177±18 | | 170±17 | | 176±18 | | 168±17 | | 154±15 | | 140±14 | | 123±12 | | 109±11 | |  |
| Total dose rate (μGy/a) | 923±48 | | 917±48 | | 1185±46 | | 1385±53 | | 1234±47 | | 1167±45 | | 1158±44 | | 1116±43 | |  |
| **Age (ka) OSL** | **12±2** | | **27±3** | | **55±4** | | **54±8** | | **148±9** | | ***134±7*** | | **162±9** | | ***165±11*** | |  |
| **SAMPLE** | | **ANY20-02 (Shfd20130)** | | **ANY20-03* (Shfd20131)** | | **ANY20-04* (Shfd20132)** | | **ANY20-05* (Shfd20133)** | | **ANY20-06 (Shfd20134)** | | **ANY20-07**  **(Shfd20135)** | | **ANY20-08* (Shfd20136)** | | **ANY20-09* (Shfd20137)** | |
| Depth (m) | | 0.85±0.1 | | 1.15±0.12 | | 0.9±0.09 | | 1.24±0.12 | | 1.9±0.2 | | 2.6±0.3 | | 3.6±0.36 | | 4.6±0.46 | |
| Water content (dry weight %) | | 11±5 | | 15±5 | | 18±5 | | 18±5 | | 19±5 | | 19±5 | | 19±5 | | 19±5 | |
| **SINGLE GRAIN** | | | | | | | | | | | | | | | | | |
| n | | 155 (134) | | 161 (135) | | 105 (99) | | 126 (112) | | 72 (66) | | 102 (97) | | 58 (49) | | 55 | |
| n (saturated) | | 17 | | 25 | | 34 | | 47 | | 50 | | 42 | | 49 | | 47 | |
| n (zero-dose) | | 0 | | 0 | | 4 | | 0 | | 0 | | 0 | | 0 | | 0 | |
| OD (%) | | 68 (47) | | 64 (48) | | 35 (31) | | 34 (12) | | 32 (15) | | 33 (30) | | 30 (23) | | 41 (39) | |
| MAM D_e_ (Gy) | | 1.98±0.21 | | 7.95±0.61 | | 9.2±1.1 | | 50.31±3.76 | | 82.87±8.75 | | 87.9±8.0 | | 140.2±17.8 | | 103.3±13.7 | |
| CAM D_e_ (Gy) | | **11.22±0.47** | | **18.56±0.85** | | **41.82±3.32** | | **75.98±2.84** | | **93.93±4.07** | | **123.6±4.9** | | **169.4±8.6** | | **184.9±13.6** | |
| FMM D_e_ (Gy) | | 12.65±0.51 | | 15.15±0.94 | | 60.26±6.64 | | 83.16±2.66 | | 89.49±4.15 | | 99.2±6.2 | | 250±36 | | 231.9±13.8 | |
| Internal dose rate (μGy/a) | | 30±1 | | 30±1 | | 30±1 | | 30±1 | | 30±1 | | 30±1 | | 30±1 | | 30±1 | |
| Alpha dose rate (μGy/a) | | 16±2 | | 16±2 | | 22±2 | | 26±3 | | 23±2 | | 22±2 | | 22±2 | | 21±2 | |
| Beta dose rate (μGy/a) | | 297±25 | | 290±25 | | 394±27 | | 478±33 | | 428±28 | | 395±27 | | 403±27 | | 395±27 | |
| Gamma dose rate (μGy/a) | | 403±36 | | 411±36 | | 563±31 | | 682±37 | | 600±32 | | 580±31 | | 580±31 | | 561±30 | |
| Cosmic dose rate (μGy/a) | | 177±18 | | 170±17 | | 176±18 | | 168±17 | | 154±15 | | 140±14 | | 123±12 | | 109±11 | |
| Total dose rate (μGy/a) | | 923±48 | | 917±48 | | 1185±46 | | 1385±53 | | 1234±47 | | 1167±45 | | 1158±44 | | 1116±43 | |
| **Age (ka) OSL** | | **12±1** | | **20±1** | | **35±3** | | **55±3** | | **76**±4 | | **106±6** | | **146±9** | | **166±14** | |

**Table S9:** ESR age estimates derived from Al and Ti centres and dose rate components. Errors are displayed at 1 sigma. In bold the data selected for the final ESR ages.

| **SAMPLE** | **ANY20-03** | **ANY20-04** | **ANY20-05** | **ANY20-08** | **ANY20-09** |
| --- | --- | --- | --- | --- | --- |
| Depth (m) | 1.15±0.12 | 0.9 ±0.09 | 1.24±0.12 | 3.6±0.36 | 4.6±0.46 |
| Water content (dry weight %) | 15±5 | 18±5 | 19 ± 5 | 19± 5 | 19±5 |
|  |  |  |  |  |  |
| D_e_ (Gy) Al | 684±180 | 810±257 | 1203±158 | 1438±263 | 1260±248 |
| D_e_ (Gy) Ti-mix (SSE W-1/I²; D_max_=4.5 kGy) | 542±39 | 562±66 | 634±34 | 1196±120 | 1116±80 |
| **D_e_ (Gy) Ti-H (SSE W-1/I²; D_max_=4.5 kGy)** | **47±15** | **52±22** | **107±25** | **240±86** | **253±75** |
| Internal dose rate (μGy/a) | 30±1 | 30±1 | 30±1 | 30±1 | 30±1 |
| Alpha dose rate (μGy/a) | 16±2 | 22±2 | 26±3 | 22±2 | 21±2 |
| Beta dose rate (μGy/a) | 290±25 | 394±27 | 478±33 | 403±27 | 395±27 |
| Gamma dose rate (μGy/a) | 411±36 | 563±31 | 682±37 | 580±31 | 561±30 |
| Cosmic dose rate (μGy/a) | 170±17 | 176±18 | 168±17 | 123±12 | 109±11 |
| Total dose rate (μGy/a) | 917±48 | 1185±46 | 1385±53 | 1158±44 | 1116±43 |
|  | | | | | |
| Age (ka) Al | 746±200 | 684±218 | 869±119 | 1242±232 | 1129±226 |
| Age (ka) Ti-mix (SSE W-1/I², D_max_=4.5 kGy) | 591±53 | 474±59 | 458±30 | 1033±111 | 1000±81 |
| **Age (ka) Ti-H (SSE W-1/I², D_max_=4.5 kGy)** | **51±17** | **44±19** | **77±18** | **207±75** | **227±68** |

**Figure S14:** SA-OSL, SG-OSL and ESR ages variation depending on the various WC values. The ages calculated with the present-day moisture are in purple and those calculated with the time-averaged moisture content (%) determined in **Table S3** are in green.


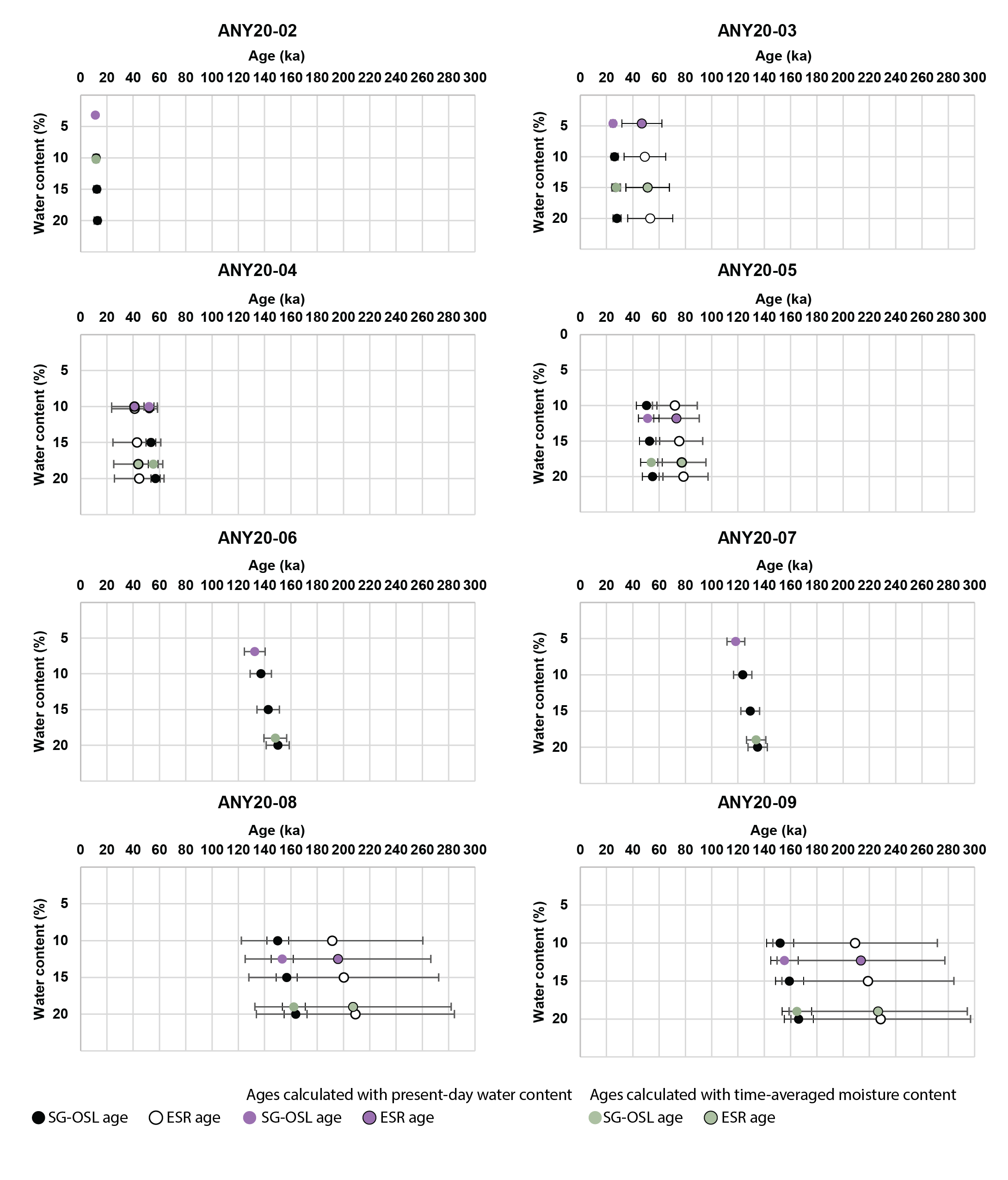


**Table S10**: SG-OSL ages recalculated with various long-term water content (WC) values WC (% dry weight).

|  |  |  |  | |
| --- | --- | --- | --- | --- |
| Sample ID | WC (% dry weight) | err (%) | SG-OSL age (ka) | err (ka) |
| ANY02 | 3.2 | 5 | 11 | 2 |
| Unit C | 9 | 5 | 12 | 2 |
|  | 10 | 5 | 12 | 2 |
|  | 15 | 5 | 12 | 2 |
|  | 20 | 5 | 13 | 2 |
| ANY03 | 4.6 | 5 | 25 | 2 |
| Unit C | 10 | 5 | 26 | 3 |
|  | 15 | 5 | 27 | 3 |
|  | 20 | 5 | 28 | 3 |
| ANY04 | 10 | 5 | 52 | 4 |
| Interface Unit C/D | 10.3 | 5 | 52 | 4 |
|  | 15 | 5 | 54 | 4 |
|  | 18 | 5 | 55 | 4 |
|  | 20 | 5 | 57 | 4 |
| ANY05 | 10 | 5 | 50 | 8 |
| Unit D1 | 11.8 | 5 | 51 | 8 |
|  | 15 | 5 | 53 | 8 |
|  | 18 | 5 | 54 | 8 |
|  | 20 | 5 | 55 | 8 |
| ANY06 | 6.9 | 5 | 132 | 8 |
| Unit D2 | 10 | 5 | 137 | 8 |
|  | 15 | 5 | 143 | 8 |
|  | 19 | 5 | 148 | 9 |
|  | 20 | 5 | 150 | 9 |
| ANY07 | 5.4 | 5 | 118 | 7 |
| Unit D2 | 10 | 5 | 124 | 7 |
|  | 15 | 5 | 129 | 7 |
|  | 19 | 5 | 134 | 7 |
|  | 20 | 5 | 135 | 7 |
| ANY08 | 10 | 5 | 150 | 8 |
| Unit D3 | 12.5 | 5 | 153 | 8 |
|  | 15 | 5 | 157 | 8 |
|  | 19 | 5 | 162 | 9 |
|  | 20 | 5 | 164 | 9 |
| ANY09 | 10 | 5 | 152 | 10 |
| Unit D3 | 12.3 | 5 | 155 | 10 |
|  | 15 | 5 | 159 | 11 |
|  | 19 | 5 | 165 | 11 |
|  | 20 | 5 | 166 | 11 |

**Table S11**: Ti-H ESR ages recalculated with various long-term water content (WC) values WC (% dry weight)

| **ESR ages** | | | | |
| --- | --- | --- | --- | --- |
| **Sample** | **WC (% dry weight)** | **err (%)** | **Age (ka)** | **err (ka)** |
| ANY20-03 | 4.6 | 5 | 47 | 15 |
| Unit C | 10 | 5 | 49 | 16 |
|  | 15 | 5 | 51 | 16 |
|  | 20 | 5 | 53 | 17 |
| ANY20-04 | 10 | 5 | 41 | 17 |
| Interface Unit C/D | 10.3 | 5 | 41 | 17 |
|  | 15 | 5 | 43 | 18 |
|  | 18 | 5 | 44 | 19 |
|  | 20 | 5 | 45 | 19 |
| ANY20-05 | 10 | 5 | 72 | 17 |
| Unit D1 | 11.8 | 5 | 73 | 17 |
|  | 15 | 5 | 75 | 18 |
|  | 18 | 5 | 77 | 18 |
|  | 20 | 5 | 79 | 19 |
| ANY20-08 | 10 | 5 | 191 | 69 |
| Unit D2 | 12.5 | 5 | 196 | 71 |
|  | 15 | 5 | 200 | 72 |
|  | 19 | 5 | 207 | 75 |
|  | 20 | 5 | 209 | 75 |
| ANY20-09 | 10 | 5 | 209 | 63 |
| Unit D3 | 12.3 | 5 | 214 | 64 |
|  | 15 | 5 | 219 | 65 |
|  | 19 | 5 | 227 | 68 |
|  | 20 | 5 | 229 | 68 |

# SI 4 - Palaeoecological Proxies

37 sediments samples (labelled from 1 to 37, **Table S1** and **S12**, **Figure 2**) along the sedimentary sequence were variously sampled for stable isotope, plant wax biomarker, macrobotanical analysis (phytoliths and pollen). Although not every sample was subjected to each type of analyses, the combination of data provides a firm basis for interpreting environmental change in the vicinity of the site and wider region.

## 4.1 Bulk Isotope Analysis

Preparation of bulk organic matter stable isotope samples, and associated analysis, took place as follows: bulk sediment samples were weighed and sieved with a 2 mm mesh to remove stones and large macrobotanical materials. 2M HCl was then added to 1 gram of each sieved sample to remove any carbonates before three rinses using deionised water interspersed by centrifuging. The remaining residues were then freeze-dried for 48 hours. 1 mg of dry samples were weighed out, in duplicate, into tin capsules. The δ^13^C measurements of the samples were measured using a Thermo Scientific Flash 2000 Elemental Analyser coupled to a Thermo Delta V Advantage mass spectrometer at the Isotope Laboratory, MPI-GEA, Jena. Isotopic values are reported as the ratio of the heavier isotope to the lighter isotope (^13^C/^12^C) as δ values in parts per mill (‰) relative to VPDB. Results were calibrated against international standards of (IAEA-CH-6: δ^13^C = -10.80 ± 0.47 ‰ and USGS40: δ^13^C = -26.38 ± 0.042) and a laboratory standard (δ^13^C = ~-15.1 ‰,). Based on replicate analyses long-term machine error over a year is ± 0.2‰ for δ^13^C. Overall measurement precision was studied through (**Table S12**).

**Table S12:** Stable carbon (δ^13^C) isotope ratios of bulk organic sediment samples from the Bété I sequence. The samples number are presented according to **Figure 2**.

| **Sample number** | **Depth (m)** | **Sedimentary Unit** | **δ^13^Ci (‰) VPDB** | **δ^13^Cii (‰) VPDB** | **δ^13^Cav (‰) VDPB** | **SD** | **%Ci** | **%Cii** | **%Cav** | **Weight sample (mg)** |
| --- | --- | --- | --- | --- | --- | --- | --- | --- | --- | --- |
| 1 | 0.1 | A | -25.7 | -25.7 | -25.7 | 0.0 | 2.3 | 3.2 | 2.8 | 3.0 |
| 2 | 0.2 | A | -25.4 | -25.6 | -25.5 | 0.1 | 2.5 | 1.8 | 2.1 | 5.6 |
| 3 | 0.3 | A | -26.1 | -26.1 | -26.1 | 0.0 | 1.1 | 1.4 | 1.3 | 4.7 |
| 4 | 0.4 | B | -25.9 | -26.0 | -26.0 | 0.1 | 1.8 | 1.7 | 1.7 | 6.0 |
| 5 | 0.5 | B | -26.7 | -26.6 | -26.7 | 0.1 | 0.0 | 0.0 | 0.0 | 10.2 |
| 6 | 0.6 | B | -25.5 | -25.5 | -25.5 | 0.0 | 1.1 | 1.2 | 1.1 | 9.6 |
| 7 | 0.7 | B | -26.8 | -26.0 | -26.4 | 0.6 | 0.0 | 0.6 | 0.3 | 5.3 |
| 8 | 0.8 | C | -26.4 | -26.3 | -26.3 | 0.0 | 1.3 | 1.2 | 1.3 | 2.9 |
| 9 | 0.9 | C | -26.1 | -26.5 | -26.3 | 0.3 | 0.1 | 0.1 | 0.1 | 14.3 |
| 10 | 1 | C | -25.4 | -25.4 | -25.4 | 0.0 | 0.7 | 0.7 | 0.7 | 9.2 |
| 11 | 1.1 | C | -26.4 | -26.6 | -26.5 | 0.1 | 0.0 | 0.0 | 0.0 | 14.4 |
| 12 | 1.2 | C | -25.5 | -25.6 | -25.6 | 0.0 | 0.6 | 0.7 | 0.6 | 10.2 |
| 13 | 1.3 | C | -25.3 | -25.4 | -25.4 | 0.1 | 0.5 | 0.5 | 0.5 | 14.3 |
| 14 | 1.4 | C/D | -26.6 | -27.1 | -26.9 | 0.4 | 0.0 | 0.0 | 0.0 | 16.3 |
| 15 | 1.5 | C/D | -26.2 | -26.6 | -26.4 | 0.3 | 0.6 | 0.2 | 0.4 | 15.2 |
| 16 | 1.6 | D1 | -26.3 | -26.3 | -26.3 | 0.0 | 1.0 | 0.9 | 0.9 | 9.9 |
| 17 | 1.7 | D1 | -26.3 | -26.4 | -26.4 | 0.0 | 1.0 | 0.8 | 0.9 | 10.8 |
| 18 | 1.8 | D1 | -26.5 | -26.1 | -26.3 | 0.3 | 0.4 | 0.7 | 0.6 | 9.2 |
| 19 | 1.9 | D1 | -26.7 | -27.1 | -26.9 | 0.3 | 0.1 | 0.1 | 0.1 | 14.8 |
| 20 | 2 | D1 | -26.1 | -26.0 | -26.1 | 0.1 | 0.3 | 0.4 | 0.3 | 14.2 |
| 21 | 2.2 | D1 | -26.7 | -26.9 | -26.8 | 0.1 | 0.2 | 0.1 | 0.1 | 15.8 |
| 22 | 2.4 | D2 | -26.5 | -26.7 | -26.6 | 0.1 | 0.2 | 0.1 | 0.2 | 14.9 |
| 23 | 2.6 | D2 | -26.5 | -26.8 | -26.7 | 0.2 | 0.3 | 0.1 | 0.2 | 14.2 |
| 24 | 2.8 | D2 | -26.6 | -26.5 | -26.5 | 0.0 | 0.2 | 0.2 | 0.2 | 15.4 |
| 25 | 3 | D2 | -26.3 | -26.7 | -26.5 | 0.3 | 0.3 | 0.2 | 0.2 | 15.0 |
| 26 | 3.2 | D2 | -26.6 | -26.5 | -26.6 | 0.0 | 0.2 | 0.1 | 0.2 | 14.4 |
| 27 | 3.4 | D2 | -26.4 | -26.6 | -26.5 | 0.1 | 0.1 | 0.2 | 0.1 | 14.6 |
| 28 | 3.6 | D2 | -26.4 | -26.5 | -26.4 | 0.1 | 0.2 | 0.2 | 0.2 | 14.4 |
| 29 | 3.8 | D2 | -27.1 | -26.9 | -27.0 | 0.1 | 0.1 | 0.1 | 0.1 | 14.4 |
| 30 | 4 | D3 | -27.5 | -27.3 | -27.4 | 0.1 | 0.2 | 0.1 | 0.1 | 14.8 |
| 31 | 4.2 | D3 | -27.6 | -27.6 | -27.6 | 0.0 | 0.5 | 0.5 | 0.5 | 10.3 |
| 32 | 4.4 | D3 | -27.5 | -27.4 | -27.5 | 0.1 | 0.2 | 0.1 | 0.1 | 14.2 |
| 33 | 4.6 | D3 | -27.3 | -27.2 | -27.3 | 0.0 | 0.5 | 0.4 | 0.5 | 15.1 |
| 34 | 4.8 | D3 | -27.4 | -27.6 | -27.5 | 0.2 | 0.0 | 0.0 | 0.0 | 14.1 |
| 35 | 5 | D3 | -26.9 | -27.3 | -27.1 | 0.3 | 0.1 | 0.3 | 0.2 | 13.9 |
| 36 | 5.2 | D3 | -27.3 | -27.7 | -27.5 | 0.3 | 0.2 | 0.1 | 0.2 | 13.8 |
| 37 | 5.4 | D3 | -27.2 | -27.1 | -27.2 | 0.1 | 0.3 | 0.3 | 0.3 | 17.0 |

## 4.2 Plant Wax Biomarker Analysis

Dry, homogenized sediments (~20 g) were extracted with a Büchi SpeedExtractor E-916 Pressurized Speed Extractor (PSE) using 9:1 (v/v) Dichloromethane:Methanol at 100 °C and 103 bar (1500 psi) in three, 10-minute cycles. Solvent containing the total lipid extract (TLE) was concentrated to ~1 mL using a Büchi Syncore­Plus evaporator and then evaporated to dryness using a steady stream of N_2_. The TLE was separated into Neutral, Acid, and Polar fractions by Aminopropyl column chromatography using 4 mL each of 2:1 Dichloromethane:Isopropanol, 4 % Acetic Acid in Diethyl Ether, and Methanol, respectively. Normal (*n*-) alkanes were isolated from the Neutral fraction using silver nitrate infused silica gel column chromatography with 4 mL Hexane. The Acid fraction was methylated with 1 mL of 2 % HCl in Methanol at 50 °C for 24 hours to convert *n*-alkanoic acids to Fatty Acid Methyl Esters (FAMEs). The FAMEs were then isolated with 1 mL of 5 % aqueous NaCl and 4 mL Hexane. The Hexane (containing FAMEs) was transferred to new vials, dried with N_2_, and then the FAMEs were further isolated from other acids using silica gel column chromatography with Hexane and DCM eluents. The DCM fraction containing the FAMEs was characterized with gas chromatography and mass spectrometry.

All plant wax biomarkers (i.e., FAMEs) were identified using Agilent MassHunter Qualitative Analysis version 8.0.0 by comparing mass spectra and retention times with an in-house external standard mixture. FAME concentrations were quantitated by comparing to an external standard mixture (Indiana F8, Arndt Schimmelmann, University of Indiana). All molecular characterization were run using PAST 4.03.

### 4.2.1. Molecular Characterization

The FAMEs were analyzed with an Agilent 7890B Gas Chromatograph (GC) System equipped with an Agilent HP-5 capillary column (30 m length, 0.25 mm i.d. and 0.25 μm film), and coupled to a 5977A Series Mass Selective Detector (MSD) at the Max Planck Institute of Geoanthropology (Jena, Germany). Samples were injected in pulsed split mode at 290 °C, and the GC oven was programmed from 60 °C (1 min hold) to 150 °C at 10 °C/min, then to 320 °C at 6 °C/min (10 min hold). Helium was the carrier gas with a constant flow of 1.1 mL/min. The MS source was operated at 230 °C with 70 eV ionization energy in the electron ionization (EI) mode and a full scan rate of *m/z* 50-650. FAMEs were identified by comparing mass spectra and retention times with an external standard mixture.

Average chain length, or the weight-averaged number of carbon homologues of the C_20_-C_34_ FAMEs, was calculated as follows:

Where C*_x_* is the abundance of the chain length with *x* carbons. ACL has been shown to be higher in C_4_ grasses^117^, but also correlates with higher growing season temperature and aridity[^118–120^](https://www.zotero.org/google-docs/?B0JFsD).

The carbon preference index (CPI), which examines the odd-over-even carbon number predominance, was calculated using the abundances of even and odd chain lengths from C_20_ to C_34_ and the following formula:

There is a wide range of CPI observed in modern African plants ranging from <1 to 99, with 96.0 % of all CPI values being greater than or equal to 1, 81.2 % greater than or equal to 2, and 60.7 % greater than or equal to 5^121^. CPI is used to examine the even-over-odd carbon number predominance and to distinguish sedimentary organic matter deriving from terrestrial plants and that from bacterial or petroleum sources^122–125^, and as an indicator for hydrocarbon maturity or degradation^126^. FAMEs deriving from land plants display carbon chains typically with CPI >5.0, while petrogenic and marine inputs or mature samples are characterized by considerably lower CPI values of ≤1.0.

The submerged/terrestrial ratio (STR), which differentiates plant waxes produced by either submerged or terrestrial plants, was calculated as follows:

The aquatic plant ratio (P_aq_), which differentiates fatty acids produced by submerged/floating plants from emergent and terrestrial plants, was calculated as follows:

Generally, *P*_aq_ values of <0.1 indicate terrestrial plant input, while values ranging between 0.1 – 0.7 signal emergent macrophytes or a mixture of inputs, and values of >0.7 point to submerged macrophyte inputs^127^. Additionally, a STR_24_ of <0.25 and a STR_24+26_ of <0.50 indicate terrestrial plant input, while values >0.25 and >0.50 signal input from submerged/floating species^128^.

### 4.2.2 Extended Results

The C_24_ FAME is most-abundant in 21 of the 31 ancient samples, while C_22_ is dominant in 9. Sample 17 has C_26_ as the most abundant FAME (**Table S13; Figure S15**). The carbon preference index (CPI) of the C_20_-C_34_ FAMEs ranges between 1.6 and 7.5 (3.8 ± 1.1, *n*=31). These values are, for the most part, typical of plant-derived CPI values and indicate that no significant plant wax degradation occurred. The relative contributions of the C_24_ and C_30_ FAMEs, biomarkers generally used as indicators for submerged/floating and terrestrial plants, respectively, are highly correlated but anti-phased (Spearman’s correlation, *r_s_* = -0.77, *p* = <0.001), co-varying in opposite directions throughout the sequence (**Figure S15**). This anti-phased relationship in the relative abundances between the C_24_ and C_30_ FAMEs may suggest that these two biomarkers came from multiple biosynthetic sources, with their proportions possibly reflecting variability in inputs from wetland and terrestrial plants, respectively. Meanwhile, the C_28_ and C_30_ FAMEs are positively correlated (Spearman’s correlation, *r_s_* = 0.47, *p* = 0.008), suggesting a similar biosynthetic source (**Figure S15**). Interestingly, C_22_ and C_24_ are anti-phased through much of the sequence, except for samples taken between 1.0 and 2.0 m depth, which co-vary in the same direction. C_24_ and C_26_ are anti-phased between 1.0 and 2.0 m, but co-vary in the same direction in the other sections of the sequence. Therefore, it appears that the C_22_, C_24_, and C_26_ FAMEs were synthesized by multiple photosynthesizing lifeforms, including aquatic, emergent, and terrestrial plants. Although these δ^13^C data suggest a C3-dominated environment, there is possibly some contribution from C_4_ plants. However, as some emergent plants, such as *Typha angustifolia*, have isotopic signals with values around -21 ‰, they can be misinterpreted as a C_4_ plant signal in palaeo-archives. As both Paq and STR suggest the presence of emergent or submerged plants, we therefore cannot rule out the influence of these plant types on δ^13^C.

**Figure S15:** Biomarker signatures of the Bété I archaeological sediments. See Section 4.2.1 for ACL and CPI calculations. Two versions of the Submerged/Terrestrial Ratio were calculated; STR_24_ and STR_24+26_. Submerged aquatic plants tend to have values greater than 0.25 and 0.50 for STR_24_ and STR_24+26_, respectively. Two versions of the Aquatic Plant Ratio (P_aq_) were also calculated to compare with STR. Terrestrial plants typically have sedimentary P_aq_ values of less than 0.1 while aquatic plants tend to have values greater than 0.7. Values ranging between 0.1 and 0.7 typically represent a mixed input that includes terrestrial, emergent, and aquatic plants. The percentages of compound abundances are relative to the dominant FAMEs (C_22_ + C_24_ + C_26_ + C_28_ + C_30_). Higher values reflect relatively increased inputs of the respective compounds.


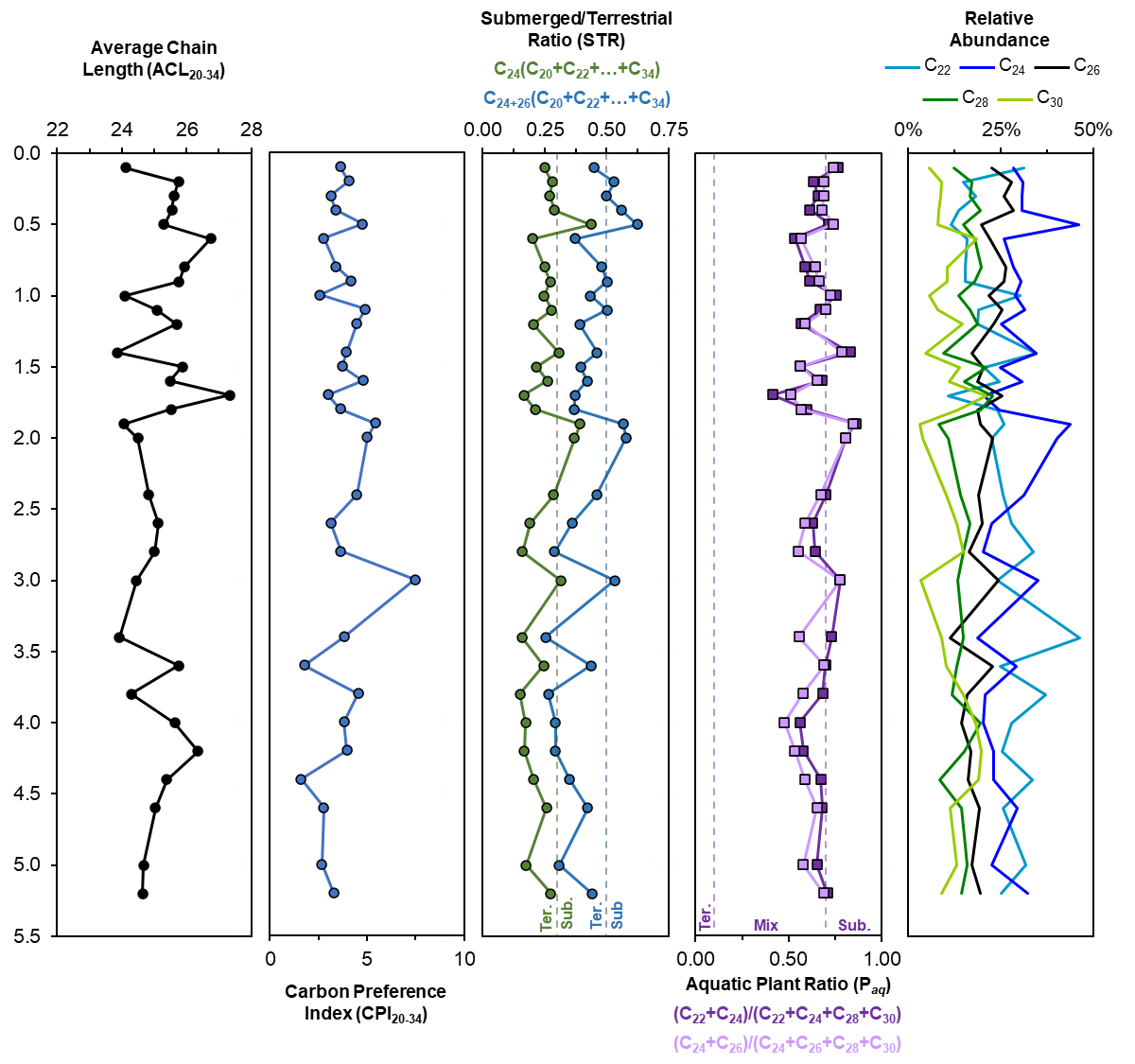


**Table S13:** Plant Wax Biomarker (FAME) metrics for each sample according to their chain length distributions. The sample numbers are presented according to **Figure 2.**

| **Sample number** | **Depth (m)** | **C Range** | **C Max** | **CPI** | **ACL** | **STR_24_** | **STR_24+26_** | **P**_aq_ **A** | **P**_aq_ **B** |
| --- | --- | --- | --- | --- | --- | --- | --- | --- | --- |
| 1 | 0.1 | 20-30 | 22 | 3.6 | 24.1 | 0.25 | 0.45 | 0.77 | 0.74 |
| 2 | 0.2 | 20-34 | 24 | 4.1 | 25.8 | 0.28 | 0.53 | 0.64 | 0.69 |
| 3 | 0.3 | 20-34 | 24 | 3.1 | 25.6 | 0.27 | 0.50 | 0.66 | 0.69 |
| 4 | 0.4 | 20-32 | 24 | 3.4 | 25.6 | 0.29 | 0.56 | 0.62 | 0.68 |
| 5 | 0.5 | 20-32 | 24 | 4.8 | 25.3 | 0.44 | 0.62 | 0.72 | 0.74 |
| 6 | 0.6 | 20-32 | 24 | 2.8 | 26.7 | 0.20 | 0.37 | 0.53 | 0.57 |
| 8 | 0.8 | 20-34 | 24 | 3.4 | 25.9 | 0.25 | 0.48 | 0.59 | 0.64 |
| 9 | 0.9 | 20, 22-32 | 24 | 4.2 | 25.8 | 0.27 | 0.50 | 0.62 | 0.66 |
| 10 | 1 | 20-30 | 22 | 2.6 | 24.1 | 0.25 | 0.43 | 0.75 | 0.72 |
| 11 | 1.1 | 20, 22-32 | 24 | 4.9 | 25.1 | 0.28 | 0.50 | 0.67 | 0.70 |
| 12 | 1.2 | 20, 22-30, 32 | 24 | 4.5 | 25.7 | 0.20 | 0.39 | 0.57 | 0.59 |
| 14 | 1.4 | 20-30 | 24 | 3.9 | 23.9 | 0.31 | 0.46 | 0.83 | 0.79 |
| 15 | 1.5 | 20-34 | 24 | 3.7 | 25.9 | 0.22 | 0.40 | 0.56 | 0.56 |
| 16 | 1.6 | 20-34 | 24 | 4.8 | 25.5 | 0.26 | 0.42 | 0.68 | 0.65 |
| 17 | 1.7 | 20, 22-30, 32 | 26 | 3.0 | 27.3 | 0.17 | 0.37 | 0.41 | 0.51 |
| 18 | 1.8 | 20-34 | 24 | 3.6 | 25.5 | 0.21 | 0.37 | 0.60 | 0.57 |
| 19 | 1.9 | 20, 22-30 | 24 | 5.4 | 24.1 | 0.39 | 0.57 | 0.86 | 0.85 |
| 20 | 2 | 20, 22-32 | 24 | 5.0 | 24.5 | 0.37 | 0.58 | 0.81 | 0.81 |
| 22 | 2.4 | 20, 22-30 | 24 | 4.5 | 24.8 | 0.29 | 0.46 | 0.70 | 0.67 |
| 23 | 2.6 | 20-32 | 22 | 3.1 | 25.1 | 0.19 | 0.36 | 0.63 | 0.59 |
| 24 | 2.8 | 20-32 | 22 | 3.7 | 25.0 | 0.16 | 0.29 | 0.64 | 0.55 |
| 25 | 3 | 20, 22-30, 32 | 24 | 7.5 | 24.4 | 0.31 | 0.53 | 0.78 | 0.78 |
| 27 | 3.4 | 20-31 | 22 | 3.8 | 23.9 | 0.16 | 0.25 | 0.73 | 0.56 |
| 28 | 3.6 | 20-32 | 24 | 1.8 | 25.8 | 0.24 | 0.44 | 0.70 | 0.69 |
| 29 | 3.8 | 20-26, 28-30, 32 | 22 | 4.6 | 24.3 | 0.15 | 0.26 | 0.68 | 0.58 |
| 30 | 4 | 20-34 | 24 | 3.8 | 25.6 | 0.17 | 0.29 | 0.56 | 0.48 |
| 31 | 4.2 | 20-34 | 22 | 4.0 | 26.4 | 0.17 | 0.29 | 0.58 | 0.53 |
| 32 | 4.4 | 20-30 | 22 | 1.6 | 25.4 | 0.21 | 0.35 | 0.67 | 0.59 |
| 33 | 4.6 | 20-32 | 24 | 2.8 | 25.0 | 0.26 | 0.42 | 0.68 | 0.65 |
| 35 | 5.0 | 20-32 | 22 | 2.7 | 24.7 | 0.17 | 0.31 | 0.65 | 0.58 |
| 36 | 5.2 | 20-32 | 24 | 3.3 | 24.6 | 0.27 | 0.44 | 0.71 | 0.69 |

STR_24_: C_24_/(C_20_+C_22_+…+C_32_+C_34_)

STR_24+26_: (C_24_+C_26_)/(C_20_+C_22_+…+C_32_+C_34_)

P_aq_ A: (C_22_+C_24_)/(C_22_+C_24_+C_28_+C_30_)

P_aq_ B: (C_24_+C_26_)/(C_24_+C_26_+C_28_+C_30_)

**Figure S16:** Typical gas chromatograph for Bété I FAME distributions. Generally, mid-chain homologues (e.g., C_20_-C_26_ FAMEs) characterize submerged and floating aquatic macrophytes and long-chain homologues (e.g., C_26_-C_32_) characterize terrestrial vegetation^129^. There is overlap in the distribution, however, as seen in FAME C_26_. Compounds C_16_ and C_18_ are ubiquitous in all lifeforms and are not diagnostic of biosynthetic origin.


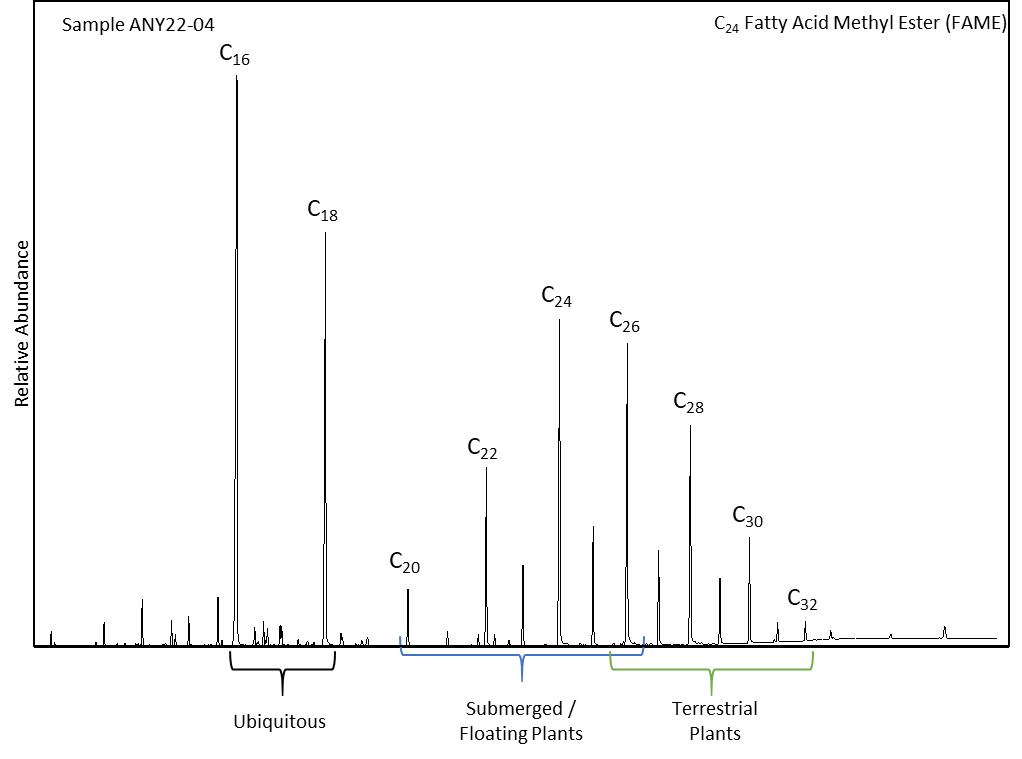


## 4.2. Phytolith analyses

The phytolith samples were processed using a standard phytolith extraction protocol as follows: (1) the sample was screened through a 0.5 mm mesh to remove coarse sized particles; (2) approximately 2gm of dried raw sediment was weighed out; (3) calcium carbonates were dissolved using a dilution of 10% hydrochloric acid and then washed in distilled water three times; (4) clay was removed using a settling procedure and sodium hexametaphosphate (Calgon) as a dispersant. Distilled water was added and the samples left for seventy-five minutes before pouring off the suspense. This was repeated at hourly intervals until the samples were clear. Samples were then transferred into crucibles and left to dry at a temperature of less than 50°C; (5) after drying, samples were placed in a muffle furnace for two hours at 500°C to remove organic matter; (6) phytoliths were then separated from the remaining material using a heavy liquid calibrated to a specific gravity of 2.3. Phytoliths were transferred to centrifuge tubes and washed three times in distilled water. They were then placed in small Pyrex beakers and left to dry; (7) approximately two milligrams of phytoliths per sample were mounted onto microscope slides, using the mounting agent Entellan. Microscope slides were assessed using a Meiji MT4300L transmitted light microscope using x100 and x400 magnifications. The phytoliths were counted and categorised into types. Phytoliths were further classified as deriving either from woody (dicotyledon) or non-woody (monocotyledon) taxa.

The weight percentage of phytoliths produced by each sediment sample is calculated by dividing the weight of phytoliths extracted from the sample by the original sample weight multiplied by 100 (**Figure S17**). This number is an approximation because other siliceous materials may also be extracted at the same time (e.g. diatoms and sponge spicules), and some residual material is likely to remain in the extracted samples. It is clear that sample 10 has a higher percentage of phytoliths in comparison to samples 21, 27 and 30. The phytolith data in **Figure S18** were plotted using C2 software.

**Figure S17:** Weight percent of phytoliths produced in samples 10, 11, 21, 22, 26, 27, 30, 31 and 36.

**
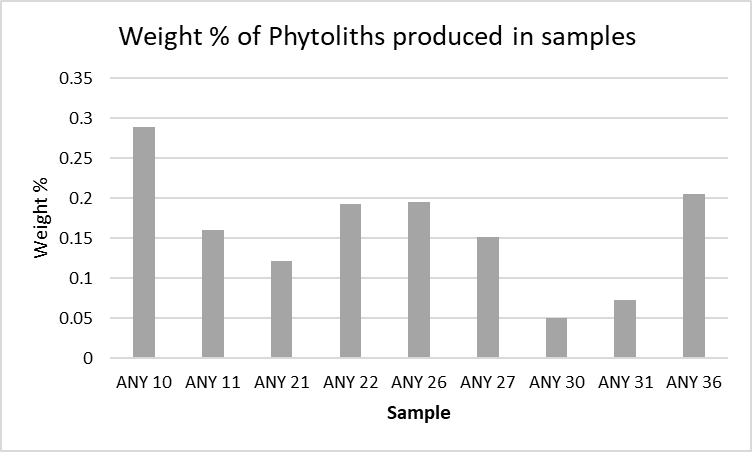
**

**Table S14:** Phytolith counts including total phytoliths counted for each slide and numbers of fragmented silica aggregate, diatoms and indication of taphonomy (e.g. burning, melting and degradation).


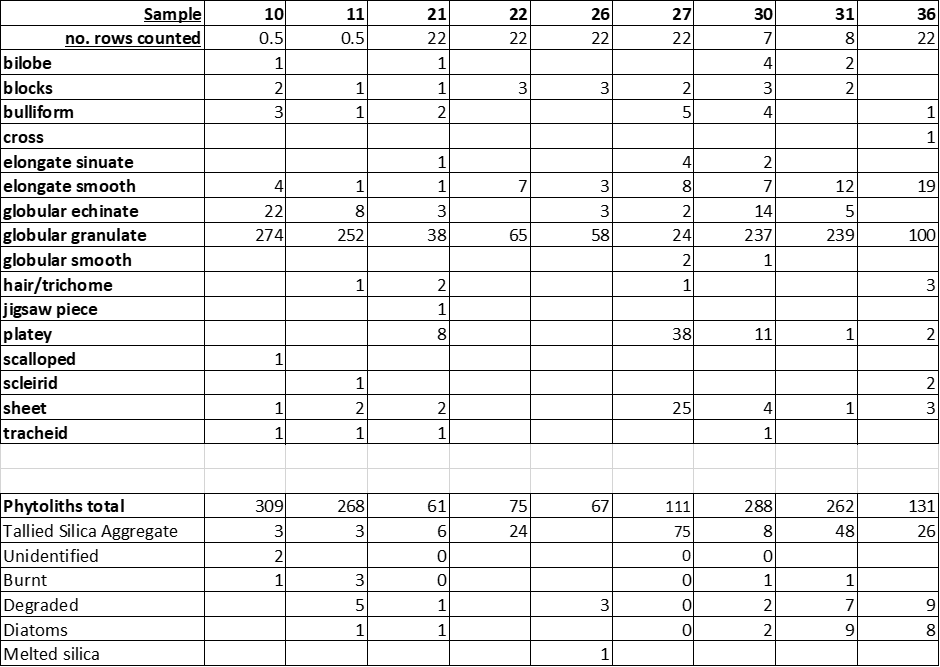


**Figure S18:** C2 diagram of percentages of phytoliths types. NB. Samples 21, 22, 26, 27 and 36 are calculated on phytolith counts below the statistically viable minimum number of recorded phytoliths so interpretation should be with caution.


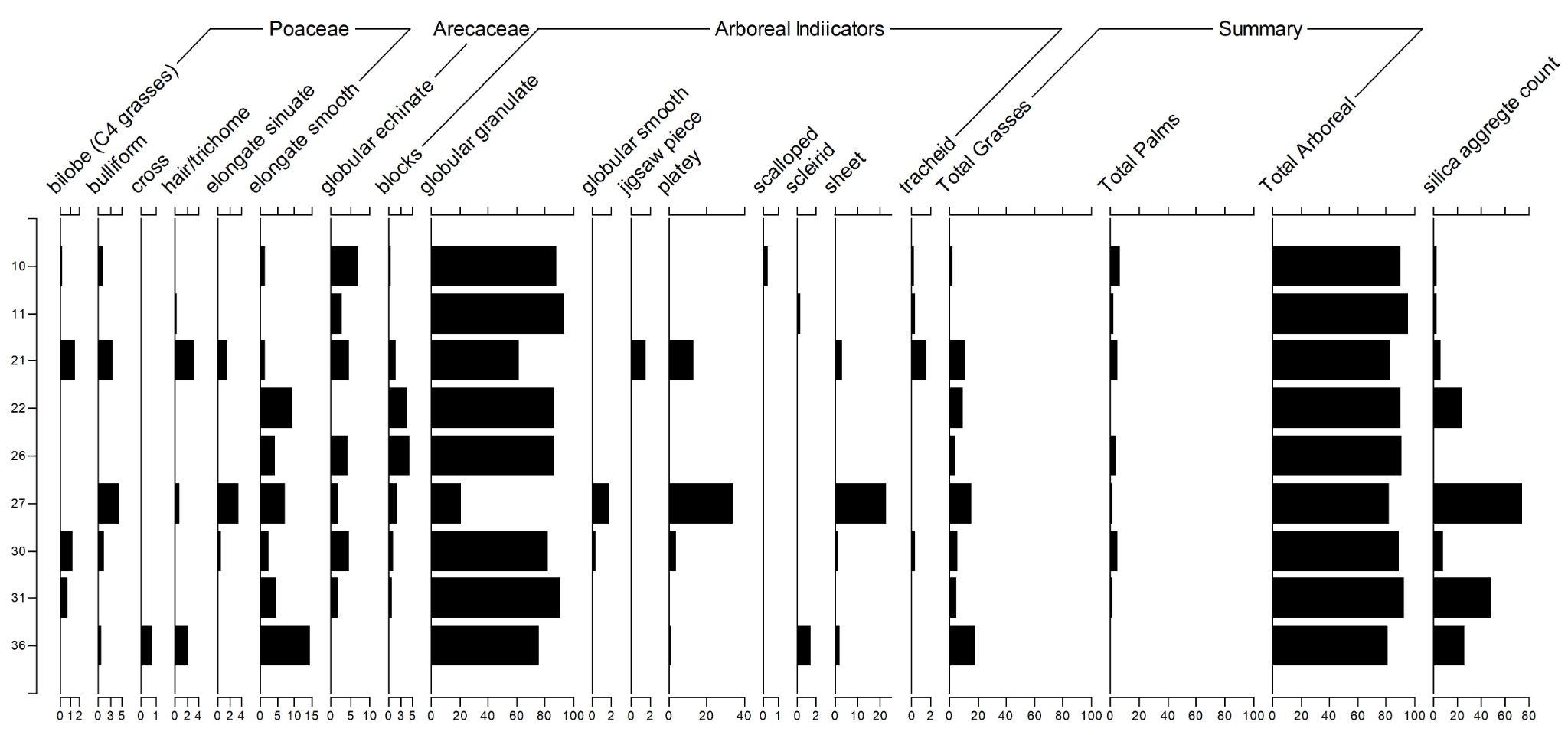


All of the arboreal phytolith types identified are produced in most dicotyledons so cannot be attributed to genus or species. All samples (**Figure S18**) are dominated by arboreal phytolith types (82-96%). Sample 10 has the most palm phytoliths (7%), samples 22 and 36 have no palm phytoliths. Sample 36 has the highest percentage of grasses (18%), followed by sample 27 (~16%) the lowest grasses being in the uppermost samples (10 and 11), there is a peak in grasses in sample 27, the remainder having between 4 and 11% grass phytoliths. Most of the grass phytoliths identified are produced in most species of grasses, however elongate smooth and sinuate can be attributed to grass leaves and stems (rather than the inflorescence). Short celled bilobe phytoliths were identified in three of the samples, these are predominantly produced in C4 grasses. Silica aggregate is produced in the bark of trees and is dominant in sample 27, and high in samples 22, 31 and 36.

Because samples 10, 11, 30 and 31 (the four samples with >250 identifiable phytoliths and therefore statistically viable) are not likely to be stratigraphically contemporary (Sample 30/31 are nearly 3m deeper than samples 10/11) we can examine the ratio of grasses, tree and palms to see if there is a change in vegetation composition by depth. Samples 30 and 31, the stratigraphically older samples, are likely to be an arboreal dominated environment with more grasses in comparison to sample 10/11 which are also arboreal dominated but with less grasses. The least palms are in sample 31 and the most in sample 10. There is also more silica aggregate (from tree bark) in the deeper samples in comparison to the samples at the top of the analysed sequence.

## 4.3. Pollen Analyses

### 4.3.1 Laboratory Methods

Analysed samples (**Table S15**) were shipped to the IASCE paleoecology laboratory at the University of South Florida, Tampa. Samples were weighed and their volume was estimated using displacement. Two tablets of Lycopodium sp. spores were added to each sample (Batch #100320201) and dissolved with 10% Hydrochloric acid (HCl). Samples were centrifugated-decanted-rinsed until pH neutral, and then placed on a shaker overnight in calgon solution (10% sodium hexametaphosphate). Fine materials were isolated using gravity separation and screening through 250µm sieves. Degraded and other complex organics were digested in a 10-minute hot bath at 80º C in a 10% Potassium Hydroxide solution. Samples were then centrifuged, the solution was decanted, and a small (~0.5 ml) volume of concentrated (27%) HCl was added to the samples. Samples were centrifugated-decanted-rinsed until their pH became neutral and the supernatant is clean. Samples were rinsed into 99% Glacial Acetic Acid, centrifugated-decanted, and then were treated using the Acetolysis reaction series. Acetolysis is triggered by the addition of a 9:1 solution of Acetic Anhydride and Sulfuric Acid, each at stock concentrations (99.5% and 48% respectively). Samples were left in a 90º C hot bath for 6 minutes, centrifugated-decanted, rinsed in Glacial Acetic Acid, and then centrifugated-decanted-rinsed until pH neutral. Microbotanical fossils were recovered from the remaining residue using density separation in a solution of 5% HCl and Zinc Bromide at a specific gravity of 2.3 g/ml. At this density, we were able to retrieve both pollen and phytoliths from the samples. This material was retrieved from the density separation in Ethanol with a pipette and then transferred into glycerin and stored in dram vials.

**Table S15**: sample numbers, depths, weights, and number of Lycopodium tracer spores added.

| **Sample number** | **Depth cm** | **Weight g.** | **Lycopodium Added** |
| --- | --- | --- | --- |
| 10 | 100 | 5 | 57138 |
| 11 | 110 | 5 | 57138 |
| 21 | 210 | 5 | 57138 |
| 22 | 220 | 5 | 57138 |
| 26 | 260 | 5 | 57138 |
| 27 | 270 | 5 | 57138 |
| 30 | 300 | 5 | 57138 |
| 31 | 310 | 5 | 57138 |
| 36 | 360 | 5 | 57138 |

### 4.3.2 Analytical Methods

Samples were mounted on glass slides in glycerin and were fixed under a cover slip with fingernail polish. Analysis was conducted using a binocular light microscope by counting the number of *Lycopodium* tracer spores, native fern spores, and pollen encountered during vertical transects across the microscope slide at 400x magnification. Pollen and spores were photographed and identified at 400x and 1000x magnification using published reference material^130–132^, online reference material (African Pollen Database, available here: https://africanpollendatabase.ipsl.fr), and unpublished digitised reference material held at Göthe Universität, Pierre et Marie Curie Université, and CEREGE. Tracers, spores, and pollen were tallied until either a total of 200 pollen were encountered or 100 Lycopodium spores were encountered. All the plots related to the pollen analysis were generated with R Statistical Software using the ‘wesanderson' package^133^.

### 4.3.3. Palynological Results

The Bété I samples (**Table S16**) show two phases of enhanced pollen preservation and concentration at 210 and 300 cm, separated by three nearly sterile samples. There is a high number of indeterminable pollen in the samples and these were classified as either entirely indeterminable or as indeterminable eudicotyledons. The latter share some morphological traits which set them apart from monocots like grasses, sedges, and palms, but which were not preserved well enough to assign them to a pollen morphotaxon. In the samples with greater preservation, the pollen types represented in the samples include fern, grass, sedge, tree palms, climbing palms, annual weeds, trees/shrubs, and climbers. Below, these results are explored in greater detail starting with assessments of fossil pollen recovery followed by a discussion of taxonomic representation in the samples.

**Table S16:** Bété I pollen, tracer spore, and fern spore counts.

| Sample number | Lyco | Ferns | Indet. | Pollen |
| --- | --- | --- | --- | --- |
| 10 | 11 | 29 | 35 | 53 |
| 11 | 55 | 84 | 70 | 157 |
| 21 | 5 | 18 | 55 | 190 |
| 22 | 98 | 1 | 5 | 18 |
| 26 | 110 | 0 | 3 | 3 |
| 27 | 111 | 2 | 5 | 2 |
| 30 | 15 | 67 | 104 | 181 |
| 31 | 326 | 26 | 24 | 26 |
| 36 | 271 | 28 | 11 | 19 |

Frequencies of *Lycopodium* tracer spores and fossil pollen/fern spores show clear differences between pollen concentrations in the samples (**Figure S19**). Pollen concentrations are calculated using the product of the ratio of the known weight of the samples and the known number of tracer spores against the ratio of fossil pollen to tracer spores encountered during analysis. Concentrations are useful for assessing the influx/preservation of pollen between samples. Given both the low preservation of these samples and the high rate of indeterminable pollen, the results will be presented as concentrations, rather than percents or other ratios derived from frequency alone. These results are presented below (**Figure S19**) on normal and logarithmic scales.

**Figure S19:** Barplot showing frequency of pollen, ferns, and tracer spores encountered during analysis.
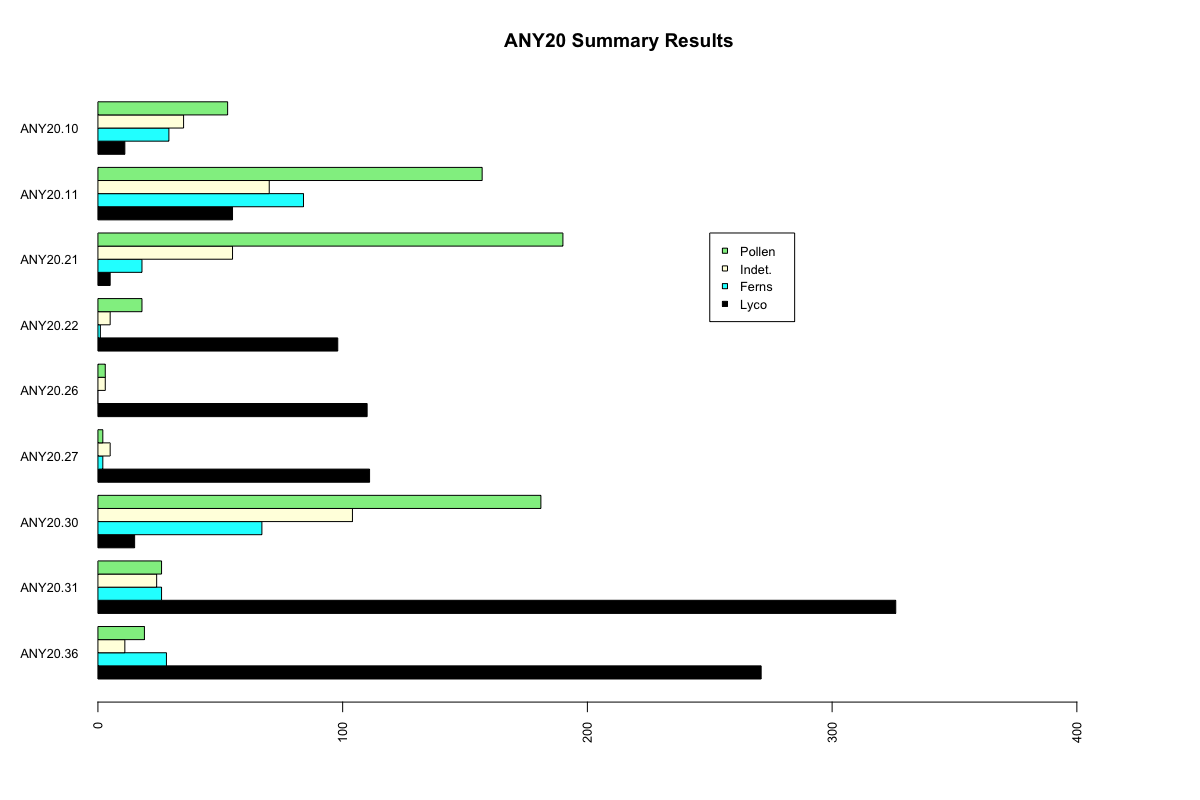


**Figure S20:** Barplots showing concentrations per gram of pollen, indeterminable palynomorphs, and ferns in Bété I samples. Left plot shows total frequencies and right plot is on logarithmic scale.
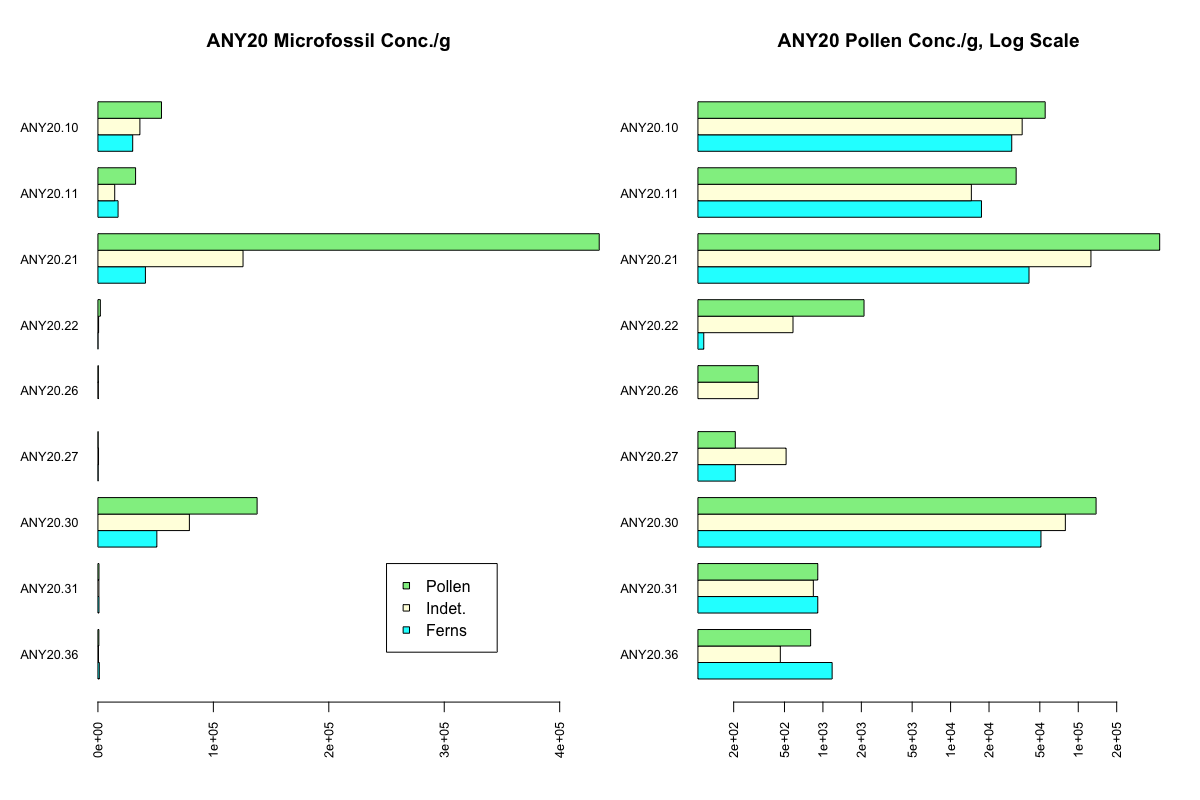


Pollen concentrations in the Bété I samples vary by orders of magnitude. The higher concentration samples also tend to have more pollen that are identifiable rather than indeterminable, which is not the case for the low concentration samples which have more indeterminable than identifiable pollen. Higher concentrations of ferns in these samples is consistent with strong taphonomic degradation, as fern spores have thick walls and higher local production. Because there are likely comparatively more ferns and they tend to preserve in greater abundances than many pollen types, we should expect higher frequencies of ferns and indeterminables in highly degraded samples. However, some Bété I samples show a lesser degree of taphonomic impact and in these samples identifiable pollen reaches 100,000/g and 400,000/g in samples 21 and 30 (**Figure S21**). These concentrations are low compared to terrestrial lake and swamp deposits, but they are comparable to terrestrial soils^134^.

Fossil pollen recovered from Bété I originates from wetland and terrestrial herbs as well as tree palms and woody plants. To better evaluate the potential impacts of taphonomic processes, indeterminable pollen were assigned to either generally indeterminate pollen or indeterminates with morphologies consistent with woody plants (*e.g.* trizonocolporate), but which cannot be assigned to an accepted morphotaxon.

**Figure S21:** Barplot of completely indeterminate and indeterminate eudicotyledons as concentration per gram.


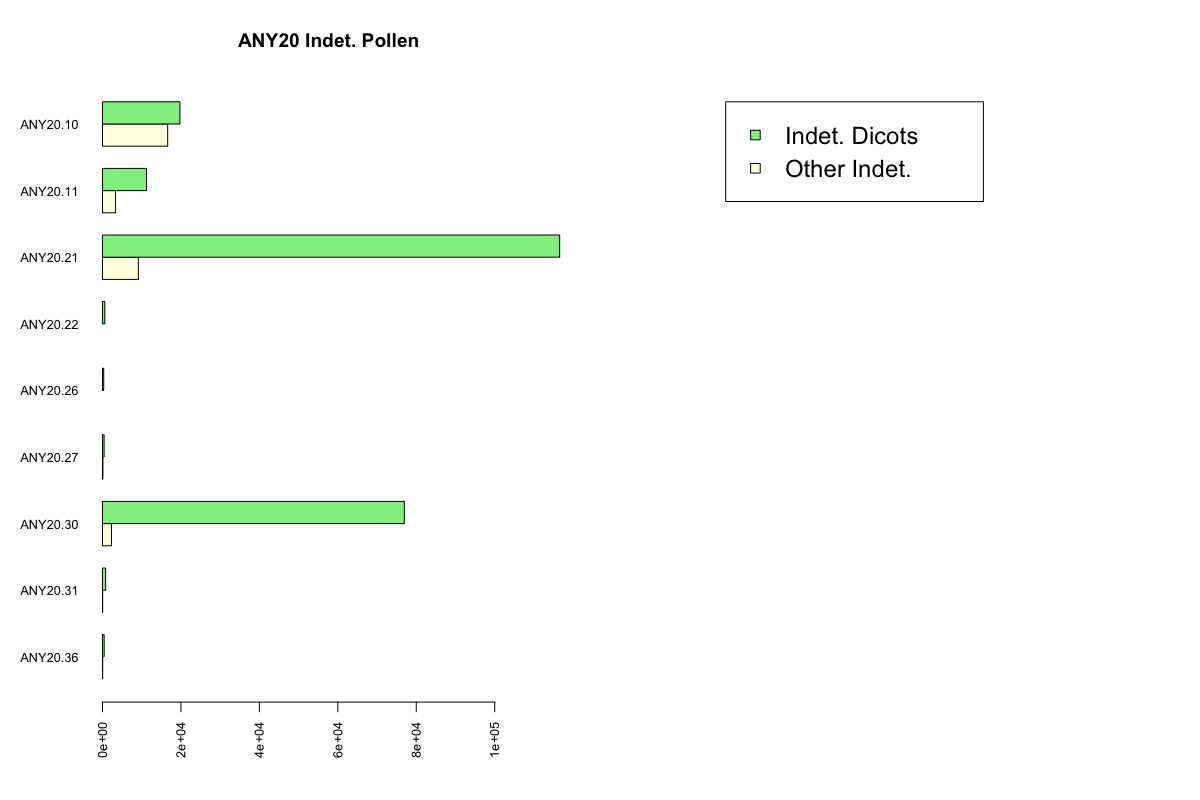


All pollen types are not uniformly produced across all plants and the size, thickness, and exine patterns of pollen contributes both to its likelihood of being preserved as well as the likelihood that it is identified. Generally, this effect tends to produce overestimates of grasses and heliophiles with thick and strongly patterned exines (*e.g.* Asteraceae or Poaceae; also see^135^). In both profiles, indeterminables follow the overall pattern of pollen concentration across the profile. In samples with modest concentrations and poor preservation, the ratio of all indeterminables to woody indeterminables is more even, reflecting the complete degradation of the pollen and loss of identifiable features. In samples with better preservation and higher pollen concentrations, there is a much better representation of woody indeterminables. This suggests that taphonomy is not the only driver of the representation of grasses and woody vegetation in these better-preserved samples, as there are similar abundances of woody plant pollen in both the damaged/indeterminable classes as well as the identifiable fraction of the pollen sample.

Identifiable pollen are grouped by a combination of their functional type and growth form into eight classes, listed below with examples of pollen morphotaxa belonging to them:

• 01_WMON : Wetland monocotyledons : Cyperaceae (sedges), *Typha* (cattail)

• 02_TMON : Terrestrial monocotyledons : Poaceae (grasses)

• 03_PALM : Palms : *Elaeis guineensis* (oil palm)

• 04_HRFB : Heliophilic shrubs, forbs, herbs : Asteraceae (sunflower), Amaranthaceae

• 05_PTSH : Pioneer trees/shrubs: *Alchornea*, *Macaranga*

• 06_TRSH : Trees/shrubs : Ebenaceae, *Hunteria*

• 07_CLPR : Climbers and/or parasites : *Corchorus*

• 08_VAR : Various form and function : Combretaceae-Melastomataceae

All eight of the growth form/functional type classes are represented in the Bété I samples, especially those with higher pollen concentrations (**Figure S22**). The distribution of these types is relatively even in the uppermost samples at sample 10 and sample 11, which also have generally lower concentrations. In sample 21, which has the highest concentrations, there is an elevated representation of trees/shrubs and pollen from various growth forms. The adjacent sample 22 has a much lower concentration of fossil pollen that is proportionally made up of more heliophilic herbs/forbs/shrubs. Low concentration and poor preservation persist through samples 26 and 27, with the former being nearly sterile. Sample 30 shows a rebound in pollen concentrations as well as the proportion of all trees/shrubs and pollen from morphotaxa with various growth forms. Samples 31 and 36 have low pollen concentrations and high proportions of indeterminate pollen, but the pollen recovered from these samples is primarily from trees/shrubs as well as heliophilic shrubs/herbs/forbs.

**Figure S22:** Plots showing pollen counts (A) and concentrations per gram (B) grouped by growth form/functional type.


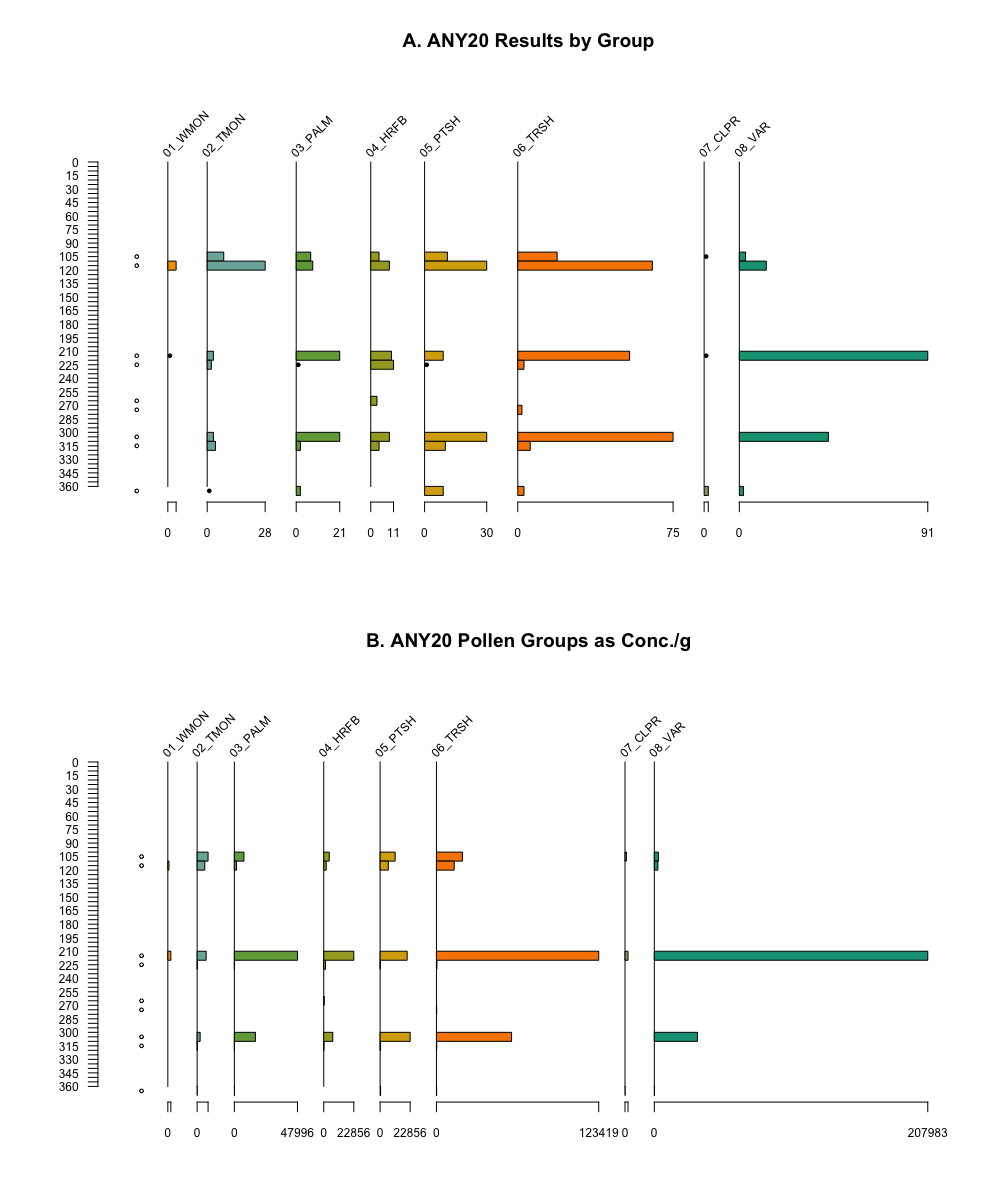


The Bété I samples yielded more than 40 different identifiable pollen morphotaxa. These are listed along with their functional types (**Table S17**).

**Table S17:** Pollen morphotaxa, family, and code for functional-type/growth form assignments.

| Pollen Taxon | Plant Family | PFT/Growth Form |
| --- | --- | --- |
| Monolete Pteridophyta | Pteridophyta | 00_FERN |
| Trilete undiff. | Pteridophyta | 00_FERN |
| Cyperaceae undiff. | Cyperaceae | 01_WMON |
| Lasimorpha senegalensis | Araceae | 01_WMON |
| Poaceae undiff. | Poaceae | 02_TMON |
| Ancistrophyllum | Arecaceae | 03_PALM |
| Elaeis guineensis | Arecaceae | 03_PALM |
| E. guineensis anther | Arecaceae | 03_PALM |
| Amaranthaceae | Amaranthaceae | 04_HRFB |
| Asteraceae anther | Asteraceae | 04_HRFB |
| Asteraceae undiff. | Asteraceae | 04_HRFB |
| Mitracarpus-type | Rubiaceae | 04_HRFB |
| Alchornea | Euphorbiaceae | 05_PTSH |
| Macaranga | Euphorbiaceae | 05_PTSH |
| Tetrorchidium didymostemon | Euphorbiaceae | 05_PTSH |
| Scottellia | Achariaceae | 06_TRSH |
| Lannea/Sclerocarya | Anacardiaceae | 06_TRSH |
| Xylopia-type | Annonaceae | 06_TRSH |
| Alstonia | Apocynaceae | 06_TRSH |
| Hunteria anthers | Apocynaceae | 06_TRSH |
| Hunteria-type umbellata | Apocynaceae | 06_TRSH |
| Landolphia | Apocynaceae | 06_TRSH |
| Rauvolfia vomitoria-type | Apocynaceae | 06_TRSH |
| Canarium-type | Burseraceae | 06_TRSH |
| Celtis | Cannabaceae | 06_TRSH |
| Parinari-type | Chrysobalanaceae | 06_TRSH |
| Pentadesma | Clusiaceae | 06_TRSH |
| Ebenaceae undiff. | Ebenaceae | 06_TRSH |
| Isoberlinia-type | Fabaceae - Caesalpinoideae | 06_TRSH |
| Dichrostachys | Fabaceae - Mimosoideae | 06_TRSH |
| Anthocleista | Gentianaceae | 06_TRSH |
| Meliaceae | Meliaceae | 06_TRSH |
| Coelocaryon preusseii | Myrsticaceae | 06_TRSH |
| Eugenia/Syzygium | Myrtaceae | 06_TRSH |
| Lophira alata | Ochnaceae | 06_TRSH |
| Uapaca | Phyllanthaceae | 06_TRSH |
| Nauclea-type | Rubiaceae | 06_TRSH |
| Sterculiaceae | Sterculiaceae | 06_TRSH |
| Musanga/Myrianthus | Urticaceae | 06_TRSH |
| Loranthaceae undiff. | Loranthaceae | 07_CLPR |
| Corchorus/Triumfetta | Malvaceae | 07_CLPR |
| Comb/Mel | Combretaceae-Melastomataceae | 08_VAR |
| Comb/Mel anther | Combretaceae-Melastomataceae | 08_VAR |
| Convolulaceae undiff. | Convolvulaceae | 08_VAR |
| Lamiaceae | Lamiaceae | 08_VAR |
| Others (< 2 IDs) | Various | 08_VAR |
| Unknowns | Various | 08_VAR |

### 4.3.4 Discussion

While the overall influx of pollen across the Bété I profile is strongly controlled by sedimentary process and overall pollen preservation, inferences about paleovegetation at Bété I may be derived from the presence of indicator taxa across the profile as well as careful interpretation of their frequencies in the two better-preserved samples.

Some pollen taxa are present across the entire profile (**Figure S23**). Grasses, *Elaeis guineensis*, Asteraceae, *Alchornea*, and *Macaranga* are present in every sample or nearly so. All of these taxa favor forest gaps and *E. guineensis*, *Alchornea*, and *Macaranga* are characteristic of early phases of swamp forest succession. In the uppermost samples 10 and 11, the presence of Cyperaceae and *Uapaca* together points to these deposits being associated with or influenced by riparian conditions.

Sample 21 is uniquely diverse and has a higher pollen concentration than any of the samples. The pollen signal shows continuity with the upper samples and includes the forest pioneers identified above with the addition of significant contributions of *Hunteria*-type pollen and a minor influx of *Musanga/Myrianthus*, *Coelocaryon preussei*, and Loranthaceae. These taxa are affiliated with tropical forests, even if *Musanga/Myrianthus* is a usually a gap colonizer in these conditions. The Loranthaceae are generally parasites and the two most widely distributed genera are *Globimetula* and *Tapinanthus*, the former is common in forest opening especially in riparian settings and the latter tends to occur in wooded savanna. *Hunteria umbellata* is a likely candidate for the source of the *Hunteria*-type pollen and this tree is characteristic of closed forests in close proximity to riparian environments^136,137^. The most abundant pollen type in the sample 21 sample is only identifiable to two related families, Combretaceae and Melastomataceae and is thus listed as Combretaceae-Melastomataceae. There are many species that can be attributed to this pollen type, but *Terminalia superba* Engl. & Diels is often associated with *H. umbellata* and many Melastomataceae are associated with channels in flooded forest settings.

**Figure S23:** Plot showing abundance of different pollen morphotaxa as concentrations per gram.


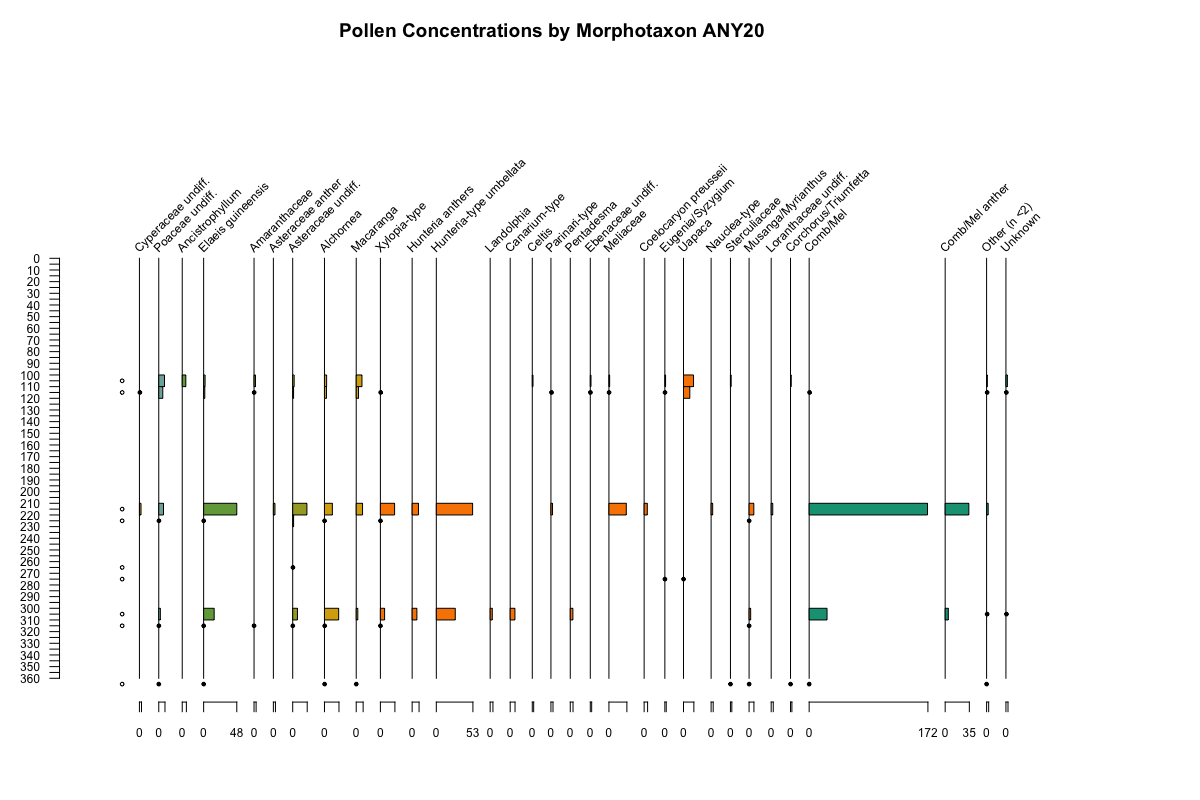


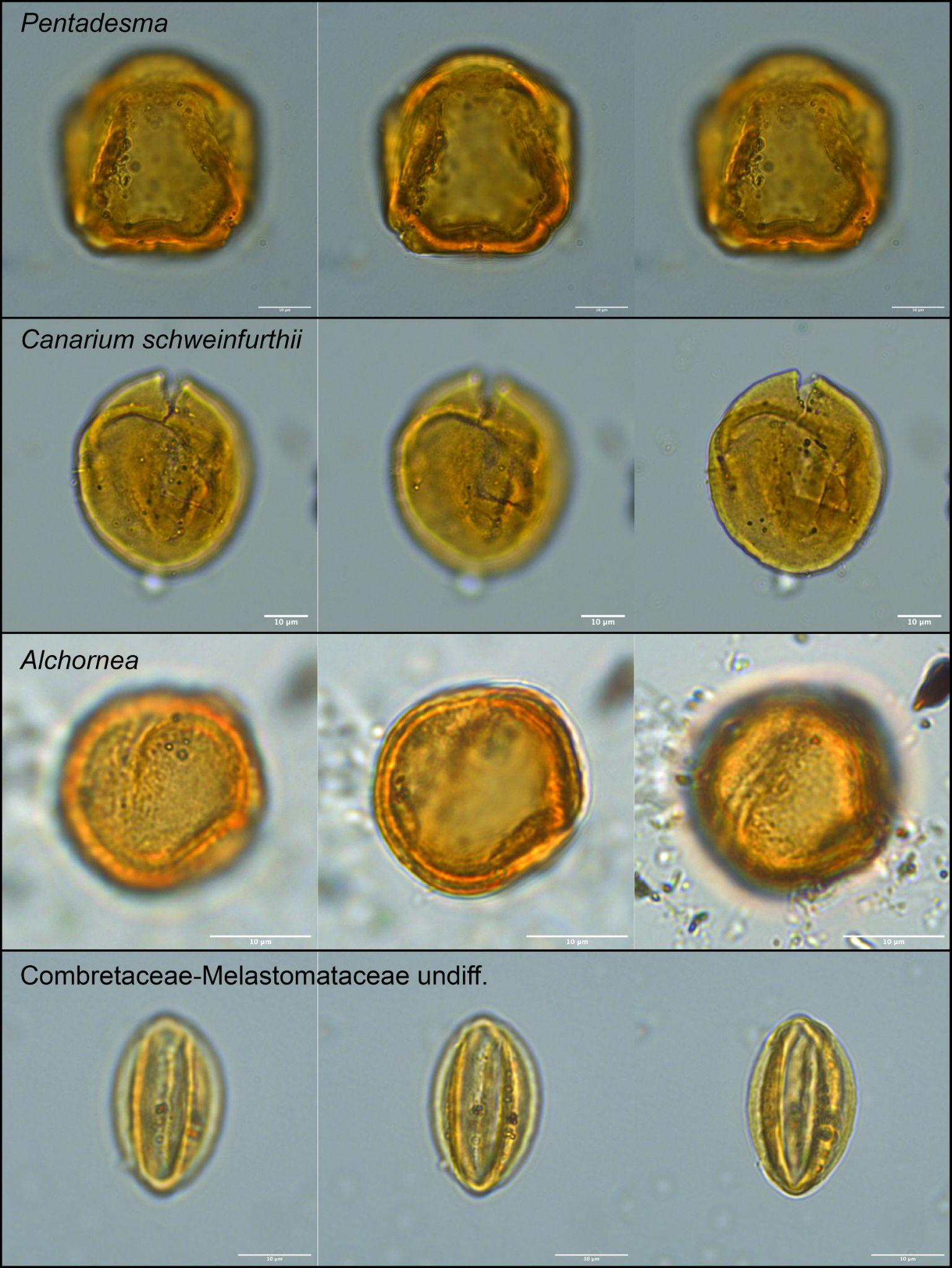
**Figure S24:** Images of important pollen types at 100x magnification.


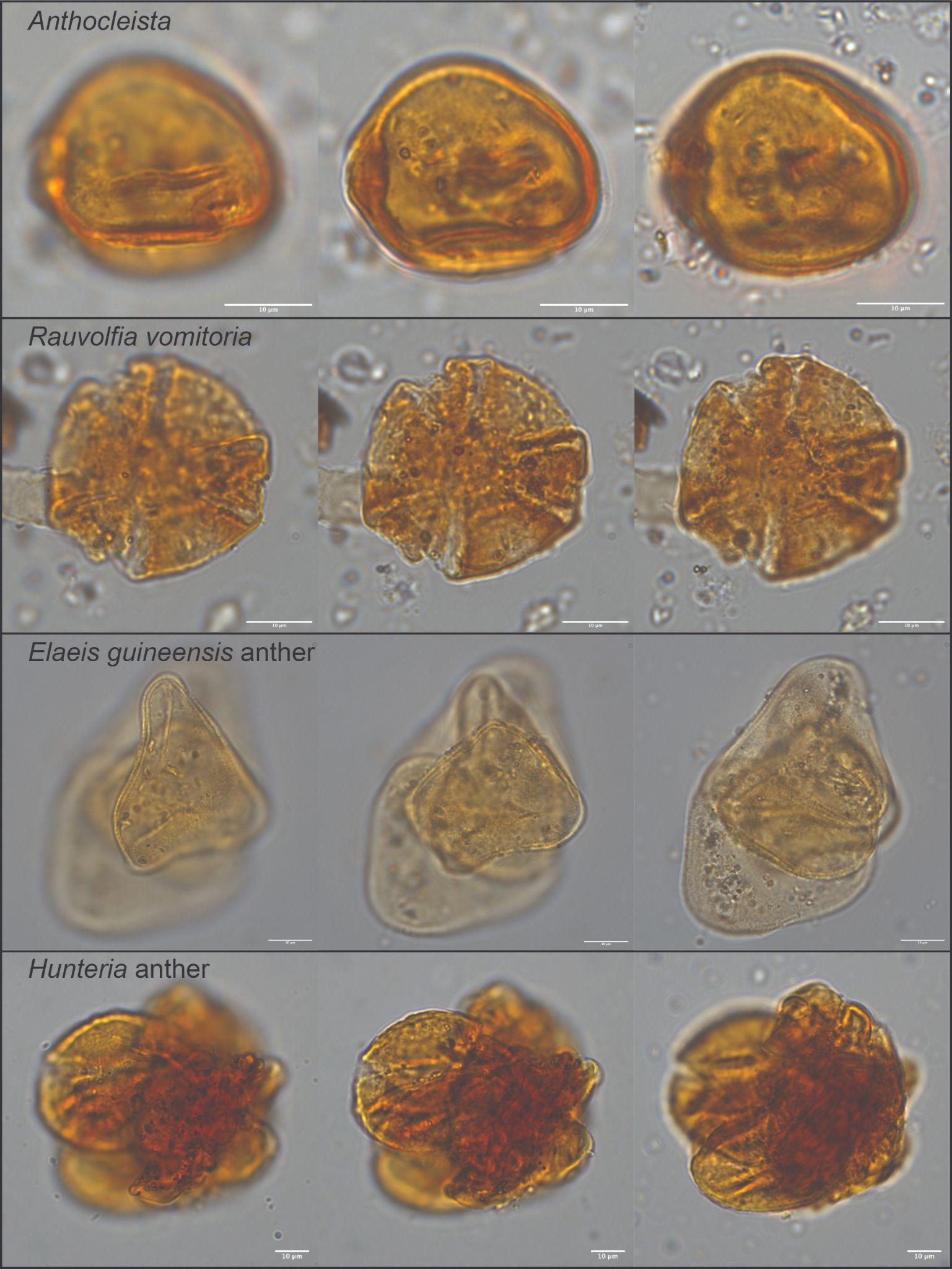
**Figure S25:** Images of important pollen types at 100x magnification, including anther fragments.

This pattern is repeated in the sample 31, although at smaller pollen concentrations and with some minor contributions from *Canarium schweinfurthii* and *Pentadesma*. The latter is likely *P. butryacea* Sabine., which is an evergreen tree with affiliations with swamp and riparian forest environments. *C. schweinfurthii* is broadly distributed across closed and open forest, but is rare outside of riparian and lacustrine settings. *C. schweinfurthii* is also part of the forest communities where *H. umbellata* and *T. superba* co-occur[^93^](https://www.zotero.org/google-docs/?KH06Ph). Grasses as well as heliophilic herbs and shrubs (*e.g.* Asteraceae undiff.) are present in this sample at low frequencies.

A unique feature of the samples 21 and 30 is a high number of anther fragments (**Figures S24 and S25**), which appear as aggregations of pollen from the same morphotaxon. These samples contain many anther fragments from Combretaceae-Melastomataceae undiff., *E. guineensis*, *H. umbellata*, and Asteraceae. These pollen types all include insect-pollinated plants and the sediment samples may be inadvertently sampling ancient insect nests. These fragments may also reflect local deposition of entire flowers and/or anthers, which are present in higher quantities because they preserve better than isolated pollen grains. The preponderance of the sedimentary, geochemical, and sedimentological evidence supports the latter conclusion, but even incidental sampling of insect nests shows that these animals were foraging in local wet forest conditions.

### 4.3.5 Conclusions

Analysis of the pollen recovered from the Bété I samples shows that these samples are strongly modified by taphonomic processes, causing the loss of a substantial portion of the pollen signal. However, these samples still yielded identifiable pollen that is useful for assessing past vegetation cover. Furthermore, taphonomic processes tend to bias pollen signals towards pollen types that are wind-pollinated with thick exines and readily identifiable features such as plants in the Poaceae, Asteraceae, and Amaranthaceae. Finally, we must also recognize that it is sufficient in this case to reject a “savanna”, “wooded savanna”, or “forest savanna mosaic” as the primary environmental context of the Bété I deposits and the pollen results are suitable for this task.

Samples with low concentrations at Bété I tend to have greater representation of monocots and heliophilic herbs/shrubs. They also have higher rates of generally indeterminable pollen and tend to have a greater representation of Poaceae and Asteraceae pollen and a lower proportion of non-monocot indeterminables. A small number of samples yielded more and better preserved pollen including both thick-walled types that are more likely to preserve (*Pentadesma*) and small thin-walled types that are less likely to preserve (*Musanga-Myrianthus*-type). What we see in both the general representation of preserved pollen as well as its proportion (**Figure S25**) is a continuous, local input of pollen from tree palms (*E. guineensis*) and other swamp forest pioneers (*Alchornea* sp., *Macaranga* sp.). In cases where pollen concentrations and counts are higher, the pollen types represented are dominated by forest types both in their frequency as well as the range of pollen morphotaxa represented in these samples. In general, there is a particularly strong representation of pollen from plants which tolerate inundation and are commonly found in forests fringing rivers, lakes, and wetlands. The strong representation of anther fragments in these samples suggests a local origin for the pollen signal. These pollen results provide strong evidence that the cultural deposits at Anyama Bété originate from a tropical rain forest environment and argue that we reject an origin from a savanna or wooded savanna environment.

**Figure S26:** Barplot showing proportional representation of key groups of pollen (left) and log values of pollen concentrations (right). Note that sample 26 and 27 do not have sufficient pollen concentrations to produce meaningful proportions.


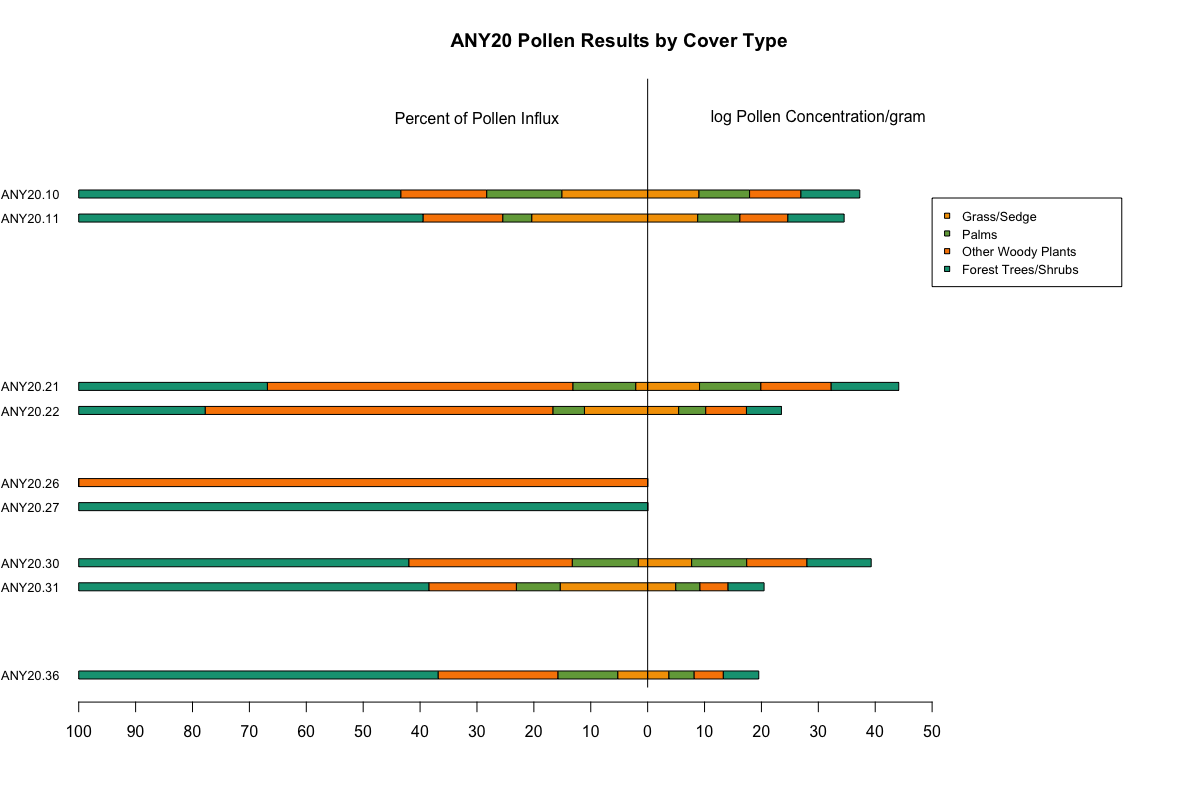


# SI 5 – Chronological comparison with others African sites

**Table S18:** Comparison chronology with other sites MSA African sites. The dates presented are clearly associated with archaeological levels/assemblages.

| Region | Country | Sub-region | Site name | Stratigraphy | Dates (ka) and dating methods direclty associated with the archaeological occurrence | | | | | | | MIS | References |
| --- | --- | --- | --- | --- | --- | --- | --- | --- | --- | --- | --- | --- | --- |
|  |  | | |  | OSL- FMM | OSL - CAM | TL | IRSL | ESR | Combined US-ESR | U-series |  |  |
| Northern Africa | Morocco | Atlantic coast | Bizmoune | 4c |  |  |  |  |  |  | 142±29 | 6 | ^138^ |
|  | Morocco | Eastern Rif | Ifri n'Ammar | Lower OS |  |  | 145±9 |  |  |  |  | 6 | ^139^ |
|  | Tunisia | Kebili | Wadi Lazalim (site 16/29) | Layer 3 |  |  |  | 149±15 |  |  |  | 6 | ^140^ |
|  |  |  |  | Layer 5 |  |  |  | 131±13 |  |  |  | 6 |  |
|  |  |  |  | Layer 6 |  |  |  | 160±14 |  |  |  | 6 |  |
|  | Egypt |  | Taramsa 1 | Unit RS |  | 165.5±17.8 |  |  |  |  |  | 6 | ^141^ |
| Eastern Africa | Soudan |  | Sai Island 8-B-11 | Unit ES and Unit S |  | Between 223±19 and 152 ± 10 |  |  |  |  |  | 6 | ^41^ |
|  | Soudan |  | Bîr Tirfawi | Grey 1 Phase |  | 135.1±21.6 |  |  |  |  |  | 6 | ^142^ |
|  | Soudan |  | Edar 135 | Unit II A – Unit II B |  | Between 194.8 ± 19.4 and 144.7 ± 19.4 |  |  |  |  |  | 6/7 | ^143^ |
|  | Soudan |  | Abdur | Coral formation |  |  |  |  |  |  | 125 ± 7 | 5/6 | ^144^ |
| Southern Africa | South Africa |  | Amanzi Springs | GBS (Area 1) | 189.7±2.8 |  |  |  |  |  |  | 6 | ^145^ |
|  | South Africa |  | Florisbad | Olive Green Sand | 157±21 |  |  |  |  |  |  | 6 | ^146^ |
|  | South Africa |  | Pinnacle Point 13B | LGB Sand 2/3 | ~174-153 |  |  |  |  |  |  | 6 | ^147^ |
|  | South Africa |  | Wonderwerk |  | ~187-132 |  |  |  |  |  |  | 6 | ^148^ |
|  | South Africa |  | Border Cave | Member 5 White Ash | 183 ± 20 |  |  |  |  |  |  | 6 | ^149^ |
|  | South Africa |  | Bundu Farm | Upper Group 4 trench 7 |  |  |  |  |  |  |  | 6 | ^150^ |
| Western Africa | Côte d'Ivoire |  | Bété I | Unit C |  | 12±1 |  |  |  |  |  | 2 | This study |
|  |  |  |  | Unit C |  | 20±1 |  |  | 51 ± 17 |  |  | 3 |  |
|  |  |  |  | Unit C/D |  | 35±3 |  |  | 44 ± 19 |  |  | 3 |  |
|  |  |  |  | Unit D1 |  | 55±3 |  |  | 77± 18 |  |  | 4 |  |
|  |  |  |  | Unit D2 |  | 76±4 |  |  |  |  |  | 5 |  |
|  |  |  |  | Unit D2 |  | 106±6 |  |  |  |  |  | 5 |  |
|  |  |  |  | Unit D3 |  | 146±9 |  |  | 207 ±75 |  |  | 6 |  |
|  | Senegal | Falémé Valley | Ravin Blanc I | SU 1 |  | 128 ± 12 |  |  |  |  |  | 5 | ^151^ |
|  |  |  |  | SU 2 |  | 124 ± 12 |  |  |  |  |  |  |  |
|  | Mali | Ounjougou | Kokolo 2 | U2 |  | 60 ± 5;  59 ± 6 |  |  |  |  |  | 4 | ^152,153^ |
|  |  |  | Oumounaama Atelier | U2 | 56 ± 6 | 64 ± 7 |  |  |  |  |  | 4 | ^152,153^ |
|  |  |  | Vipère 1, Lower and Upper | U3 | 57 ± 5 | 55 ± 5 |  |  |  |  |  | 4 | ^152,153^ |
|  |  |  | Kondo | U3 | 45 ± 5 | 59 ± 6 |  |  |  |  |  | 4 | ^153,154^ |
|  | Mali | Yawa | Songona 1 | F3A |  | 44 ± 4 |  |  |  |  |  | 3 | ^154,155^ |
|  | Mali | Dogon | Draperies | U5 | 43 ± 5 | 43 ± 5 |  |  |  |  |  | 3 | ^152,153^ |
|  |  |  | Oumounaama butte | U6 | 41 ± 6 | 43 ± 6 |  |  |  |  |  | 3 | ^153,154^ |
|  |  |  | Oumounaama Px | U5 | 40 ± 3; 33 ± 3 | 40 ± 3;39 ± 3 |  |  |  |  |  | 3 | ^153,154^ |
|  |  |  | Orosobo | U5 | 44 ± 4; 45 ± 4 | 39 ± 3;27 ± 3 |  |  |  |  |  | 3 | ^152,153^ |
|  |  |  | Oumounaama Coupe Frank | U3 | 41 ± 4 | 18 ± 2; 19 ± 3 |  |  |  |  |  | 3 | ^152,153^ |
|  |  |  | Oumounaama Coupe Frank, lower | U4 | 43 ± 4; 37 ± 5 | 47 ± 4;48 ± 6 |  |  |  |  |  |  |  |
|  |  |  | Oumounaama Coupe Frank, upper |  | 37 ± 3 | 23 ± 3 |  |  |  |  |  |  |  |
|  |  |  | Kokolo 3 c.3 | U5 | U5: 31 ± 3 | U5: 33 ± 3 |  |  |  |  |  | 3 | ^152,153^ |
|  |  |  |  |  | U5: 33 ± 4 | U5: 26 ± 3 |  |  |  |  |  |  |  |
|  |  |  | Dandoli 1 |  | U5: 30 ± 4 | U5: 34 ± 4 |  |  |  |  |  | 3 | ^152,153^ |
|  |  |  |  |  | U5: 36 ± 4 | U5: 35 ± 4 |  |  |  |  |  |  |  |
|  |  |  | Dandoli 2 | U5 | 30 ± 3;32 ± 3 | 30 ± 3 |  |  |  |  |  | 3 | ^152,153^ |
|  |  |  | Sinkarma 1 | U5 | 34 ± 4 | 23 ± 3 |  |  |  |  |  | 3 | ^152,153^ |
|  | Senegal | Western coast | Tiémassas |  | S7: 61.9 ± 2.6 |  |  |  |  |  |  | 4 | ^156,157^ |
|  |  |  |  |  | S6: 47.3 ± 2.9 |  |  |  |  |  |  | 3 |  |
|  |  |  |  |  | S6: 43.8 ± 2.81 |  |  |  |  |  |  | 3 |  |
|  |  |  |  |  | S5: 25.9 ± 1.3 |  |  |  |  |  |  | 3 |  |
|  |  |  | Bargny 1 | US 5 |  | 144±6 |  |  | 138±14 |  |  | 6 | ^158,159^ |
|  |  |  | Bargny 3 | US 5 |  | 126±7 128±8 |  |  | 115±13134±15 |  |  | 6-5 | ^159^ |
|  | Senegal | Falémé Valley | Toumboura III | Uj | 35 ± 3 |  |  |  |  |  |  | 3 | ^160,161^ |
|  |  |  |  |  | 33 ± 3 |  |  |  |  |  |  |  |  |
|  |  |  | Missira III | Uj | 37 ± 4 |  |  |  |  |  |  | 3 | ^160^ |
|  | Ghana | Northern Region | Birimi, Upper | Layer 3 | 40.8 ± 11.4 |  |  |  |  |  |  | 2 | ^162–164^ |
|  |  |  |  |  | 105 cm: 23.6 ± 2.9 |  |  |  |  |  |  |  |  |
|  | Senegal | Gambia river | Laminia | 1B | 22 ± 0.85 |  |  |  |  |  |  | 2 | ^165^ |
|  |  |  |  |  | 20.8 ± 0.83 |  |  |  |  |  |  |  |  |

# References

1. Hublin, J.-J. *et al.* New fossils from Jebel Irhoud, Morocco and the pan-African origin of Homo sapiens. *Nature* **546**, 289–292 (2017).

2. Richter, D. *et al.* The age of the hominin fossils from Jebel Irhoud, Morocco, and the origins of the Middle Stone Age. *Nature* **546**, 293–296 (2017).

3. Scerri, E. M. L. *et al.* Did our species evolve in subdivided populations across Africa, and why does it matter? *Trends Ecol. Evol.* **33**, 582–594 (2018).

4. Scerri, E. M. L., Chikhi, L. & Thomas, M. G. Beyond multiregional and simple out-of-Africa models of human evolution. *Nature Ecology and Evolution* vol. 3 1370–1372 at https://doi.org/10.1038/s41559-019-0992-1 (2019).

5. Ragsdale, A. P. *et al.* A weakly structured stem for human origins in Africa. *Nat. 2023 6177962* **617**, 755–763 (2023).

6. Bergström, A., Stringer, C., Hajdinjak, M., Scerri, E. M. L. & Skoglund, P. Origins of modern human ancestry. *Nature* vol. 590 229–237 at https://doi.org/10.1038/s41586-021-03244-5 (2021).

7. Deino, A. L. *et al.* Chronology of the Acheulean to Middle Stone Age transition in eastern Africa. *Science (80-. ).* **360**, 95–98 (2018).

8. Scerri, E. M. L. & Will, M. The revolution that still isn’t: The origins of behavioral complexity in Homo sapiens. *J. Hum. Evol.* **179**, (2023).

9. Hladik, A. & Dounias, E. WILD YAMS OF THE AFRICAN FOREST AS POTENTIAL FOOD RESOURCES. **13**, 163–177 (1993).

10. Roberts, P. & Petraglia, M. Pleistocene rainforests: barriers or attractive environments for early human foragers? *World Archaeol.* **47**, 718–739 (2015).

11. Mercader, J. Forest people: The role of African rainforests in human evolution and dispersal. *Evol. Anthropol. Issues, News, Rev.* **11**, 117–124 (2002).

12. Yasuoka, H. Concentrated distribution of wild yam patches: Historical ecology and the subsistence of African rainforest hunter-gatherers. *Hum. Ecol.* **37**, 577–587 (2009).

13. Roberts, P. *Tropical forests in prehistory, history, and modernity*. (2019).

14. Roberts, P. *Jungle: how tropical forests shaped the world - and us*. (2021).

15. Scerri, E. M. L., Roberts, P., Maezumi, S. Y. & Malhi, Y. Tropical forests in the deep human past. *Philos. Trans. R. Soc. B Biol. Sci.* **377**, (2022).

16. Sato, H. The potential of edible wild yams and yam-like plants as a staple food resource in the African tropical rain forest. *Afr. Study Monogr.* **Suppl.26**, 123–134 (2001).

17. Wedage, O. *et al.* Specialized rainforest hunting by Homo sapiens ~45,000 years ago. *Nat. Commun.* **10**, 1–8 (2019).

18. Roberts, P. *et al.* Fruits of the forest: Human stable isotope ecology and rainforest adaptations in Late Pleistocene and Holocene (36 to 3 ka) Sri Lanka. *J. Hum. Evol.* **106**, 102–118 (2017).

19. Westaway, K. E. *et al.* An early modern human presence in Sumatra 73,000-63,000 years ago. *Nature* **548**, 322–325 (2017).

20. Barker, G. *et al.* The ‘human revolution’ in lowland tropical Southeast Asia: the antiquity and behavior of anatomically modern humans at Niah Cave (Sarawak, Borneo). *J. Hum. Evol.* **52**, 243–261 (2007).

21. Summerhayes, G. R. *et al.* Human adaptation and plant use in Highland New Guinea 49,000 to 44,000 years ago. *Science (80-. ).* **330**, 78–81 (2010).

22. Roosevelt, A. C. The Rise and Fall of the Amazon Rubber Industry. *L’Homme* 255–283 (1993) doi:10.4324/9781315179971.

23. Roberts, P. & Stewart, B. A. Defining the ‘generalist specialist’ niche for Pleistocene Homo sapiens. *Nat. Hum. Behav. 2018 28* **2**, 542–550 (2018).

24. Cerasoni, J. N. *et al.* Late Pleistocene to Holocene palaeoenvironmental reconstruction and human behaviour at Iho Eleru rock shelter, Nigeria. in *11th Annual meeting of the European Society for the Study of the Human Evolution* (2021).

25. Harvati, K. *et al.* The Later Stone Age Calvaria from Iwo Eleru, Nigeria: Morphology and Chronology. *PLoS One* **6**, e24024 (2011).

26. Cornelissen, E. The later Pleistocene in the Northeastern Central African rainforest. *Vertebr. Paleobiol. Paleoanthropology* 301–319 (2016) doi:10.1007/978-94-017-7520-5_16/COVER.

27. Shipton, C. *et al.* 78,000-year-old record of Middle and Later stone age innovation in an East African tropical forest. *Nat. Commun.* **9**, 1832 (2018).

28. Roberts, P. *et al.* Mapping our reliance on the tropics can reveal the roots of the Anthropocene. *Nat. Ecol. Evol. 2023 75* **7**, 632–636 (2023).

29. Blome, M. W., Cohen, A. S., Tryon, C. A., Brooks, A. S. & Russell, J. The environmental context for the origins of modern human diversity: A synthesis of regional variability in African climate 150,000-30,000 years ago. *J. Hum. Evol.* **62**, 563–592 (2012).

30. Taylor, N. Across Rainforests and Woodlands: a systematic re-appraisal of the Lupemban Middle Stone Age in central Africa. in *Africa from MIS 6-2: Population Dynamics and Paleoenvironments* (eds. Jones, S. C. & Stewart, B. A.) 273–299 (2016). doi:10.1007/978-94-017-7520-5.

31. Kaboth-Bahr, S. *et al.* Paleo-ENSO influence on African environments and early modern humans. *Proc. Natl. Acad. Sci. U. S. A.* **118**, (2021).

32. Clark, J. D. *The prehistory of southern Africa*. (1959).

33. McBrearty, S. The Sangoan‐Lupemban and middle stone age sequence at the Muguruk site, western Kenya. *World Archaeol.* **19**, 388–420 (1988).

34. Wayland, E. J. & Smith, R. Some primitive stone tools from Uganda. *Geol. Surv. Uganda Occas. Pap.* **1**, (1923).

35. Davies, O. West Africa before the europeans: Archaeology & prehistory. *West Africa before Eur. Archaeol. Prehistory* **19**, 1–367 (1967).

36. Taylor, N. Riddles wrapped inside an enigma. Lupemban MSA technology as a rainforest adaptation: Revisiting the lanceolate point. *Philos. Trans. R. Soc. B Biol. Sci.* **377**, (2022).

37. Padilla-Iglesias, C., Grove, M. & Blinkhorn, J. Ecological drivers of hunter-gatherer lithic technology from the Middle and Later Stone Age in Central Africa. *Quat. Sci. Rev.* **322**, 108390 (2023).

38. McBrearty, S. & Brooks, A. S. The revolution that wasn’t: a new interpretation of the origin of modern human behavior. *J. Hum. Evol.* **39**, 453–563 (2000).

39. Lioubin, V. P. & Guédé, F. Y. *Paleolit Respubliki Kot d’Ivvuar (Zapadnaya Afrika) [The Palaeolithic of the Republic of Cote d’Ivoire (West Africa)]*. *Russian Academy of Sciences, Institute of the History of Material Culture Proceedings, vol. 3* (2000).

40. Van Peer, P. *et al.* The Early to Middle Stone Age Transition and the emergence of modern human behaviour at site 8-B-11 Sai Island, Sudan. *J. Hum. Evol.* **45**, 187–193 (2003).

41. Van Peer, P. Sai Island (Site 8-B-11), Sudan. *Handb. Pleistocene Archaeol. Africa* 1013–1025 (2023) doi:10.1007/978-3-031-20290-2_66.

42. Fisher, E. C. *et al.* Archaeological Reconnaissance for Middle Stone Age sites along the Pondoland coast, South Africa. *PaleoAnthropology* 104–137 (2013) doi:10.4207/PA.2013.ART82.

43. Kuman, K., Le Baron, J. C. & Gibbon, R. J. Earlier stone age archaeology of the Vhembe-Dongola National Park (South Africa) and vicinity. *Quat. Int.* **129**, 23–32 (2005).

44. Wurz, S. The Early Middle Stone Age in South Africa. *Oxford Res. Encycl. Anthropol.* (2020) doi:10.1093/ACREFORE/9780190854584.013.118.

45. Sheppard, P. J. & Kleindienst, M. R. Technological change in the earlier and middle stone Age of Kalambo Falls (Zambia). *African Archaeol. Rev.* **13**, 171–196 (1996).

46. Clark, J. D. *Kalambo Falls - Prehistoric Site - Volume 3: The Earlier Cultures: Middle and Earlier Stone Age*. (University of California, 2001).

47. Barham, L., Tooth, S., Duller, G. A. T., Plater, A. J. & Turner, S. Excavations at Site C North, Kalambo Falls, Zambia: New insights into the mode 2/3 transition in South-Central Africa. *J. African Archaeol.* **13**, 187–214 (2015).

48. Duller, G. A. T., Tooth, S., Barham, L. & Tsukamoto, S. New investigations at Kalambo Falls, Zambia: Luminescence chronology, site formation, and archaeological significance. *J. Hum. Evol.* **85**, 111–125 (2015).

49. Mcbrearty, S. Sangoan technology and Habitat at Simbi. *Nyame akuma* **39**, 38–40 (1993).

50. Kuman, K. The earlier Stone Age in South Africa: site context and the influence of 496 cave studies. in *Breathing Life Into 497 Fossils: Taphonomic Studies in Honor of C. K. (Bob) Brain* (eds. Pickering, T. R., Schick, K. & Toth, N.) 181–198 (Gosport, 2007).

51. Barham, L. *The Middle Stone Age of Zambia, South-Central Africa*. (2000).

52. Herries, A. I. R. A Chronological Perspective on the Acheulian and Its Transition to the Middle Stone Age in Southern Africa: The Question of the Fauresmith. *Int. J. Evol. Biol.* **2011**, 961401 (2011).

53. Shea, J. J. Sink the Mousterian? Named stone tool industries (NASTIES) as obstacles to investigating hominin evolutionary relationships in the Later Middle Paleolithic Levant. *Quat. Int.* **350**, 169–179 (2014).

54. Wilkins, J. Is it Time to Retire NASTIES in Southern Africa? Moving Beyond the Culture-Historical Framework for Middle Stone Age Lithic Assemblage Variability. *Lithic Technol.* **45**, 295–307 (2020).

55. Andah, B. W. The Early Palaeolithic in West Africa: the case of Asokrochonacoastal region of Accra, Ghana in perspectives on West Africa’s past. *West Afr. J.Archaeol* **9**, 47–85 (1979).

56. Nygaard, S. E. & Talbot, M. R. Stone age archaeology and environment on the southern accra plains, ghana. *Nor. Archaeol. Rev.* **17**, 19–38 (1984).

57. Soper, R. C. The Stone Age in Northern Nigeria. *J. Hist. Soc. Niger* **3**, 175–194 (1965).

58. Clark, J. D. The Sangoan culture of Equatoria: the implications of its stone equipment. in *Miscellanea en Homenaje al Abate Henri Breuil.Barcelona: Instituto de Prehistoria y Arcqueologia Monografias, 9: 30* (1964).

59. Clark, J. D. The later Pleistocene cultures of Africa. *Science (80-. ).* **160**, 833–847 (1965).

60. Clark, J. D. *The Prehistory of Africa*. (1970).

61. Shea, J. J. Prehistoric Stone Tools of Eastern Africa: A Guide. *Prehist. Stone Tools East. Africa* (2020) doi:10.1017/9781108334969.

62. Terrazas-Mata, A. *et al.* Middle Stone Age at Equatorial Guinea: Technical and use-wear analysis of lithic bifacial points. *Anthropol.* **127**, (2023).

63. Rots, V. & Van Peer, P. Early evidence of complexity in lithic economy: core-axe production, hafting and use at Late Middle Pleistocene site 8-B-11, Sai Island (Sudan). *J. Archaeol. Sci.* **33**, 360–371 (2006).

64. Allsworth-Jones, P. *The Middle Stone Age of Nigeria in its West African Context*. (2019).

65. Chenorkian, R. & Paradis, G. Une industrie paleolithique decouverte dans la ‘terre dc barre’ d’une terrasse proche d’Anyama (region d’Abidjan). in *3rd Congress, West African Archaeological Association, Dakar.* (1981).

66. Chenorkian, R. & Paradis, G. Une industrie paléolithique découverte dans la « Terre de Barre » d’une terrasse proche d’Anyama (région d’Abidjan). *Nyame Akuma* **21**, 18–27 (1982).

67. Fick, S. E. & Hijmans, R. J. WorldClim 2: new 1-km spatial resolution climate surfaces for global land areas. *Int. J. Climatol.* **37**, 4302–4315 (2017).

68. Fick, S. E. & Hijmans, R. J. WorldClim 2: new 1‐km spatial resolution climate surfaces for global land areas. *Int. J. Climatol.* **37**, 4302–4315 (2017).

69. N’Zi, D. C. & Guédé, F. Y. Recherches prehistoriques en Cote d’Ivoire : non-developpements recents sur le site d’Anyama (district d’Abidjan). *Anthropologie.* **127**, (2023).

70. Blott, S. J. & Pye, K. Gradistat: A grain size distribution and statistics package for the analysis of unconsolidated sediments. *Earth Surf. Process. Landforms* **26**, 1237–1248 (2001).

71. Buylaert, J. P., Murray, A. S., Thomsen, K. J. & Jain, M. Testing the potential of an elevated temperature IRSL signal from K-feldspar. *Radiat. Meas.* **44**, 560–565 (2009).

72. BATEMAN, M. D. & CATT, J. A. An absolute chronology for the raised beach and associated deposits at Sewerby, East Yorkshire, England. *J. Quat. Sci.* **11**, 389–395 (1996).

73. Bateman, M. D., Frederick, C. D., Jaiswal, M. K. & Singhvi, A. K. Investigations into the potential effects of pedoturbation on luminescence dating. *Quat. Sci. Rev.* **22**, 1169–1176 (2003).

74. Duller, G. A. T. Single-grain optical dating of Quaternary sediments: why aliquot size matters in luminescence dating ? *Boreas* **37**, 589–612 (2008).

75. Murray, A. S. & Wintle, A. G. The single aliquot regenerative dose protocol: Potential for improvements in reliability. *Radiat. Meas.* **37**, 377–381 (2003).

76. Murray, A. S. & Wintle, A. G. Luminescence dating of quartz using an improved single-aliquot regenerative-dose protocol. *Radiat. Meas.* **32**, 57–73 (2000).

77. Murray, A., Buylaert, J. P., Henriksen, M., Svendsen, J. I. & Mangerud, J. Testing the reliability of quartz OSL ages beyond the Eemian. *Radiat. Meas.* **43**, 776–780 (2008).

78. Duller, G. A. T. Distinguishing quartz and feldspar in single grain luminescence measurements. *Radiat. Meas.* **37**, 161–165 (2003).

79. Duller, G. The Analyst software package for luminescence data: overview and recent improvements. *Anc. TL* **33**, 35–42 (2015).

80. Duller, G. A. T. Analyst User Manual. (2017).

81. Burow, C. *et al.* RLumShiny - A graphical user interface for the R Package ‘Luminescence’. *Anc. TL* **34**, 22–32 (2016).

82. Kreutzer, S. *et al.* Comprehensive Luminescence Dating Data Analysis [R package Luminescence version 0.9.26]. *CRAN Contrib. Packag.* (2024) doi:10.32614/CRAN.PACKAGE.LUMINESCENCE.

83. Duller, G. A. T., Bøtter-Jensen, L., Murray, A. S. & Truscott, A. J. Single grain laser luminescence (SGLL) measurements using a novel automated reader. *Nucl. Instruments Methods Phys. Res. Sect. B Beam Interact. with Mater. Atoms* **155**, 506–514 (1999).

84. Toyoda, S., Voinchet, P., Falgueres, C., Dolo, J.-M. & Laurent, M. Bleaching of ESR signals by the sunlight : a laboratory experiment for establishing the ESR dating of sediments. *Appl. Radiat. Isot.* **52**, 1357–1362 (2000).

85. Duval, M. Dose response curve of the ESR signal of Aluminum center in quartz grains extracted from sediment Dose response curve of the ESR signal of the Aluminum center in quartz grains extracted from sediment. *Anc. TL* **2**, 1–10 (2012).

86. Guilarte, V. & Duval, M. ESR dating of optically bleached quartz grains: Assessing the impact of different experimental setups on dose evaluations. *Geochronometria* **1695**, 0–6 (2020).

87. Toyoda, S. & Falguères, C. The method to represent the ESR signal intensity of the aluminium hole center in quartz for the purpose of dating. *Adv. ESR Appl.* **20**, 7–10 (2003).

88. Ben Arous, E., Duval, M. & Bateman, M. D. ESR dating of optically bleached quartz grains from Plio-Pleistocene to Holocene coastal dune deposits (Wilderness-Knysna area, South Africa): a comparison with luminescence. *Quat. Geochronol.* 101293 (2022) doi:10.1016/j.quageo.2022.101293.

89. Duval, M. Evaluating the accuracy of ESR dose determination of pseudo-Early Pleistocene fossil tooth enamel samples using dose recovery tests. *Radiat. Meas.* **79**, 24–32 (2015).

90. Duval, M. & Guilarte Moreno, V. Assessing the influence of the cavity temperature on the ESR signal of the Aluminum center in quartz grains extracted from sediment. *Anc. TL* **30**, 11–16 (2012).

91. Woda, C. & Wagner, G. A. Non-monotonic dose dependence of the Ge- and Ti-centres in quartz. *Radiat. Meas.* **42**, 1441–1452 (2007).

92. Yokoyama, Y., Falguères, C. & Quaegebeur, J. P. ESR dating of quartz from Quaternary sediments : first attempts. *Nucl. Tracks* **10**, 921–928 (1985).

93. Guérin, G., Mercier, N. & Adamiec, G. Dose-rate conversion factor: update. *Anc. TL* **29**, 5–8 (2011).

94. Duval, M. *et al.* Quantifying hydrofluoric acid etching of quartz and feldspar coarse grains based on weight loss estimates: implication for ESR and luminescence dating studies. *Anc. TL* **36**, (2018).

95. Brennan, B. J. Beta doses to spherical grains. *Radiat. Meas.* **37**, 299–303 (2003).

96. Brennan, B. J., Lyons, R. G. & Phillips, S. W. Attenuation of alpha particle track dose for spherical grains. *Int. J. Radiat. Appl. Instrumentation. Part D. Nucl. Tracks Radiat. Meas.* **18**, 249–253 (1991).

97. Duval, M. *et al.* Re-examining the earliest evidence of human presence in western Europe: New dating results from Pirro Nord (Italy). *Quat. Geochronol.* **82**, (2024).

98. Bartz, M. *et al.* First experimental evaluation of the alpha efficiency in coarse-grained quartz for ESR dating purposes: implications for dose rate evaluation. *Sci. Rep.* **9**, (2019).

99. Durcan, J. A., King, G. E. & Duller, G. A. T. DRAC: Dose Rate and Age Calculator for trapped charge dating. *Quat. Geochronol.* **28**, 54–61 (2015).

100. Prescott, J. R. & Hutton, J. T. Cosmic ray and gamma ray dosimetry for TL and ESR. *Int. J. Radiat. Appl. Instrumentation. Part D. Nucl. Tracks Radiat. Meas.* **14**, 223–227 (1988).

101. Prescott, J. R. & Hutton, J. T. Cosmic ray contributions to dose rates for luminescence and ESR dating: Large depths and long-term time variations. *Radiat. Meas.* **23**, 497–500 (1994).

102. Duval, M. *et al.* ESR dating of optically-bleached quartz grains: Evaluating measurement repeatability and reproducibility. *Radiat. Phys. Chem.* **215**, (2024).

103. Duval, M. & Guilarte, V. ESR dosimetry of optically bleached quartz grains extracted from Plio-Quaternary sediment: Evaluating some key aspects of the ESR signals associated to the Ti-centers. *Radiat. Meas.* **78**, 28–41 (2015).

104. Duval, M., Arnold, L. J. & Rixhon, G. Electron spin resonance (ESR) dating in Quaternary studies: evolution, recent advances and applications. *Quaternary International* vol. 556 1–10 at https://doi.org/10.1016/j.quaint.2020.07.044 (2020).

105. Bartz, M. *et al.* Successful combination of electron spin resonance, luminescence and palaeomagnetic dating methods allows reconstruction of the Pleistocene evolution of the lower Moulouya river (NE Morocco). *Quat. Sci. Rev.* **185**, 153–171 (2018).

106. Méndez-Quintas, E. *et al.* First evidence of an extensive Acheulean large cutting tool accumulation in Europe from Porto Maior (Galicia, Spain). *Sci. Rep.* **8**, (2018).

107. Ben Arous, E., Bateman, M. D. & Duval, M. Extending the ESR and OSL dating comparison on coastal dune deposits from the Wilderness-Knysna area (South Africa). *Quat. Geochronol.* 101580 (2024) doi:10.1016/J.QUAGEO.2024.101580.

108. Duval, M. *et al.* Electron spin resonance dating of optically bleached quartz grains from the Middle Palaeolithic site of Cuesta de la Bajada (Spain) using the multiple centres approach. *Quat. Geochronol.* **37**, 82–96 (2017).

109. Bartz, M. *et al.* Testing the potential of K-feldspar pIR-IRSL and quartz ESR for dating coastal alluvial fan complexes in arid environments. *Quat. Int.* **556**, 124–143 (2020).

110. Hoaglin, D. C., Mosteller, F. & Tukey, J. W. *Understanding robust and exploratory data analysis*. (Wiley, 1983).

111. Boulter, C., Bateman, M. D. & Frederick, C. D. Understanding geomorphic responses to environmental change: a 19 000-year case study from semi-arid central Texas, USA. *J. Quat. Sci.* **25**, 889–902 (2010).

112. Galbraith, R. F., Roberts, R. G., Laslett, G. M., Yoshida, H. & Olley, J. M. Optical dating of single and multiple grains of quartz from Jinmuim rockshelter, northern Australia : Part I, experimental design and statistical models. *Archaeometry* **41**, 339–364 (1999).

113. Roberts, R. G., Galbraith, R. F., Yoshida, H., Laslett, G. M. & Olley, J. M. Distinguishing dose populations in sediment mixtures: A test of single-grain optical dating procedures using mixtures of laboratory-dosed quartz. *Radiat. Meas.* **32**, 459–465 (2000).

114. Boulter, C. H. Reconstructing the palaeoenvironmental dynamics of East Central Texas since the Last Glacial Maximum. Unpublished PhD Thesis. (University of Sheffield, 2007).

115. Arnold, L. J. *et al.* OSL dating of individual quartz ‘supergrains’ from the Ancient Middle Palaeolithic site of Cuesta de la Bajada, Spain. *Quat. Geochronol.* **36**, 78–101 (2016).

116. Galbraith, R. F. & Roberts, R. G. Statistical aspects of equivalent dose and error calculation and display in OSL dating: An overview and some recommendations. *Quat. Geochronol.* **11**, 1–27 (2012).

117. Rommerskirchen, F., Plader, A., Eglinton, G., Chikaraishi, Y. & Rullkötter, J. Chemotaxonomic significance of distribution and stable carbon isotopic composition of long-chain alkanes and alkan-1-ols in C4 grass waxes. *Org. Geochem.* **37**, 1303–1332 (2006).

118. Dodd, R. S. & Poveda, M. M. Environmental gradients and population divergence contribute to variation in cuticular wax composition in Juniperus communis. *Biochem. Syst. Ecol.* **11**, 1257–1270 (2003).

119. Bush, R. T. & McInerney, F. A. Influence of temperature and C4 abundance on n-alkane chain length distributions across the central USA. *Org. Geochem.* **79**, 65–73 (2015).

120. Tipple, B. J. & Pagani, M. Environmental control on eastern broadleaf forest species’ leaf wax distributions and d/h ratios. *Geochim. Cosmochim. Acta* **111**, 64–77 (2013).

121. Bush, R. T. & McInerney, F. A. Leaf wax n-alkane distributions in and across modern plants: Implications for paleoecology and chemotaxonomy. *Geochim. Cosmochim. Acta* **117**, 161–179 (2013).

122. Diefendorf, A. F., Freeman, K. H., Wing, S. L. & Graham, H. V. Production of n-alkyl lipids in living plants and implications for the geologic past. *Geochim. Cosmochim. Acta* **75**, 7472–7485 (2011).

123. Jaeschke, A. *et al.* Influence of land use on distribution of soil n-alkane δD and brGDGTs along an altitudinal transect in Ethiopia: Implications for (paleo)environmental studies. *Org. Geochem.* **124**, 77–87 (2018).

124. Castañeda, I. S. & Schouten, S. A review of molecular organic proxies for examining modern and ancient lacustrine environments. *QSRv* **30**, 2851–2891 (2011).

125. Bray, E. E. & Evans, E. D. Distribution of n -paraffins as a clue to recognition of source beds. *GeCoA* **22**, 2–15 (1961).

126. Duan, Y. & He, J. Distribution and isotopic composition of n-alkanes from grass, reed and tree leaves along a latitudinal gradient in China. *Geochem. J.* **45**, 199–207 (2011).

127. Ficken, K. J., Li, B., Swain, D. L. & Eglinton, G. An n-alkane proxy for the sedimentary input of submerged/floating freshwater aquatic macrophytes. *Org. Geochem.* **31**, 745–749 (2000).

128. Liu, H. & Liu, W. Concentration and distributions of fatty acids in algae, submerged plants and terrestrial plants from the northeastern Tibetan Plateau. *Org. Geochem.* **113**, 17–26 (2017).

129. Patalano, R., Roberts, P., Boivin, N., Petraglia, M. D. & Mercader, J. Plant wax biomarkers in human evolutionary studies. *Evol. Anthropol. Issues, News, Rev.* **30**, 385–398 (2021).

130. Caratini, C. & Guinet, P. Pollen et Spores d ’ Afrique tropicale. *Assoc. des Palynol. Lang. Fr.* (1974).

131. Ybert, J. . *Atlas de pollens de cote d’Ivoire*. (1979).

132. Gosling, W. D., Miller, C. S. & Livingstone, D. A. Atlas of the tropical West African pollen flora. *Rev. Palaeobot. Palynol.* **199**, 1–135 (2013).

133. Ram, K. & Wickham, H. A Wes Anderson Palette Generator [R package wesanderson version 0.3.7]. *CRAN Contrib. Packag.* (2023) doi:10.32614/CRAN.PACKAGE.WESANDERSON.

134. DIMBLEBY, G. W. Pollen Analysis of Terrestrial Soils. *New Phytol.* **56**, 12–28 (1957).

135. Bush, M. B. On the interpretation of fossil Poaceae pollen in the lowland humid neotropics. *Palaeogeogr. Palaeoclimatol. Palaeoecol.* **177**, 5–17 (2002).

136. Onyekwelu, J. C., Mosandl, R. & Stimm, B. No Title. *J. Trop. For. Sci.* **20**, 193–204 (2008).

137. Owusu, G., Anning, A. K., Belford, E. J. D. & Acquah, E. Plant species diversity, abundance and conservation status of the Ankasa Resource Reserve, Ghana. *Trees, For. People* **8**, 100264 (2022).

138. Sehasseh, E. M. *et al.* Early middle stone age personal ornaments from Bizmoune Cave, Essaouira, Morocco. *Sci. Adv.* **7**, 8620–8642 (2021).

139. Richter, D., Moser, J., Nami, M., Eiwanger, J. & Mikdad, A. New chronometric data from Ifri n’Ammar (Morocco) and the chronostratigraphy of the Middle Palaeolithic in the Western Maghreb. *J. Hum. Evol.* **59**, 672–679 (2010).

140. Cancellieri, E. *et al.* A late Middle Pleistocene Middle Stone Age sequence identified at Wadi Lazalim in southern Tunisia. *Sci. Rep.* **12**, 3996 (2022).

141. Van Peer, P., Vermeersch, P. M. & Paulissen, E. Chert Quarrying, Lithic Technology and a Modern Human Burial at the Palaeolithic Site of Taramsa 1, Upper Egypt . Philip Van Peer , Pierre M. Vermeersch , Etienne Paulissen. *Leuven Univ. Press* **67**, 288–289 (2010).

142. Nicoll, K. A revised chronology for Pleistocene paleolakes and Middle Stone Age – Middle Paleolithic cultural activity at Bîr Tirfawi – Bîr Sahara in the Egyptian Sahara. *Quat. Int.* **463**, 18–28 (2018).

143. Michalec, G. *et al.* A Window into the Early–Middle Stone Age Transition in Northeastern Africa—A Marine Isotope Stage 7a/6 Late Acheulean Horizon from the EDAR 135 Site, Eastern Sahara (Sudan). *https://doi.org/10.1080/00934690.2021.1993618* **46**, 513–533 (2021).

144. Walter, R. C. *et al.* Early human occupation of the Red Sea coast of Eritrea during the last interglacial. *Nature* **405**, 65–69 (2000).

145. Herries, A. I. R. *et al.* *A marine isotope stage 11 coastal Acheulian workshop with associated wood at Amanzi Springs Area 1, South Africa*. *PLoS ONE* vol. 17 (2022).

146. Grun, R. Direct dating of Florisbad hominid. *Science* vol. 382 500–5001 at (1996).

147. Marean, C. W. Pinnacle Point Cave 13B (Western Cape Province, South Africa) in context: The Cape Floral kingdom, shellfish, and modern human origins. *J. Hum. Evol.* **59**, 425–443 (2010).

148. Beaumont, P. B. & Vogel, J. C. On a timescale for the past million years of human history in central South Africa. *S. Afr. J. Sci.* **102**, 217–228 (2006).

149. Wadley, L. *et al.* Fire and grass-bedding construction 200 thousand years ago at Border Cave, South Africa. *Science (80-. ).* **369**, 863–866 (2020).

150. Kiberd, P. Bundu Farm: A Report on Archaeological and Palaeoenvironmental Assemblages from a Pan Site in Bushmanland, Northern Cape, South Africa. *South African Archaeol. Bull.* **61**, 189–201 (2006).

151. Douze, K. *et al.* A West African Middle Stone Age site dated to the beginning of MIS 5: Archaeology, chronology, and paleoenvironment of the Ravin Blanc I (eastern Senegal). *J. Hum. Evol.* **154**, 102952 (2021).

152. Soriano, S., Rasse, M., Tribolo, C. & Huysecom, E. Ounjougou: A long Middle Stone Age sequence in the Dogon country (Mali). in *West African Archaeology. New Developments, New Perspectives* (ed. Allsworth-Jones, P.) 1–14 (Archaeopress, 2010).

153. Tribolo, C., Rasse, M., Soriano, S. & Huysecom, E. Defining a chronological framework for the Middle Stone Age in West Africa: Comparison of methods and models for OSL ages at Ounjougou (Mali). *Quat. Geochronol.* **29**, 80–96 (2015).

154. Chevrier, B. *et al.* Between continuity and discontinuity: An overview of the West African Paleolithic over the last 200,000 years. *Quat. Int.* **466**, 3–22 (2018).

155. Rasse, M., Tribolo, C., Soriano, S. & Huysecom, E. Premières données chronostratigraphiques sur les formations du Pléistocène supérieur de la « falaise » de Bandiagara (Mali, Afrique de l’Ouest). *http://journals.openedition.org/quaternaire* **23**, 5–23 (2012).

156. Niang, K., Blinkhorn, J. & Ndiaye, M. The oldest Stone Age occupation of coastal West Africa and its implications for modern human dispersals: New insight from Tiémassas. *Quat. Sci. Rev.* (2018) doi:10.1016/j.quascirev.2018.03.022.

157. Niang, K. *et al.* The Middle Stone Age occupations of Tiémassas, coastal West Africa, between 62 and 25 thousand years ago. *J. Archaeol. Sci. Reports* **34**, 102658 (2020).

158. Niang, K., Blinkhorn, J., Bateman, M. D. & Kiahtipes, C. A. Longstanding behavioural stability in West Africa extends to the Middle Pleistocene at Bargny, coastal Senegal. *Nat. Ecol. Evol. 2023* 1–11 (2023) doi:10.1038/s41559-023-02046-4.

159. Ben Arous, E. *et al.* Constraining the age of the Middle Stone Age locality of Bargny sites (Senegal) through a combined OSL-ESR dating approach. *Quat. Environ. Humans* (2024) doi:doi.org/10.1016/j.qeh.2024.100044.

160. Lebrun, B. *et al.* Establishing a West African chrono-cultural framework: First luminescence dating of sedimentary formations from the Falémé Valley, Eastern Senegal. *J. Archaeol. Sci. Reports* **7**, 379–388 (2016).

161. Chevrier, B. *et al.* West African Palaeolithic history: New archaeological and chronostratigraphic data from the Falémé valley, eastern Senegal. *Quat. Int.* **408**, 33–52 (2016).

162. Hawkins, A., Casey, J. L., Godfrey-smith, D. & D’Andrea, A. C. A Middle Stone Age Component at the Birimi Site, Northern Region, Ghana. *Nyame akuma* **46**, 1–3 (1996).

163. Casey, J. *et al.* Report of investigations at the Birimi Site in Northern Ghana. *Nyame akuma* 32–38 (1997).

164. Quickert, N. A., Godfrey-Smith, D. I. & Casey, J. L. Optical and thermoluminescence dating of Middle Stone Age and Kintampo bearing sediments at Birimi, a multi-component archaeological site in Ghana. in *Quaternary Science Reviews* vol. 22 1291–1297 (Elsevier Ltd, 2003).

165. Scerri, E. M. L. *et al.* Continuity of the Middle Stone Age into the Holocene. *Sci. Rep.* **11**, 70 (2021).
