# Supplementary material for: Humans in Africa’s wet tropical forests 150 thousand years ago
Source: Nature. 2025 Feb 26;640(8058):402–7. doi: 10.1038/s41586-025-08613-y (PMC11981921; doi:10.1038/s41586-025-08613-y)
Supplement: Supplementary file 2 — Reporting Summary [file 41586_2025_8613_MOESM2_ESM.pdf]

nature portfolio

Corresponding author(s): Eslem BEN AROUS, James. A. BLINKHORN, E. SCERRI

Last updated by author(s): Dec 13, 2024

Reporting Summary

Nature Portfolio wishes to improve the reproducibility of the work that we publish. This form provides structure for consistency and transparency in reporting. For further information on Nature Portfolio policies, see our Editorial Policies and the Editorial Policy Checklist.

Statistics

For all statistical analyses, confirm that the following items are present in the figure legend, table legend, main text, or Methods section.

n/a	Confirmed	
☐	☒	The exact sample size (*n*) for each experimental group/condition, given as a discrete number and unit of measurement
☒	☐	A statement on whether measurements were taken from distinct samples or whether the same sample was measured repeatedly
☒	☐	The statistical test(s) used AND whether they are one- or two-sided<br>*Only common tests should be described solely by name; describe more complex techniques in the Methods section.*
☒	☐	A description of all covariates tested
☒	☐	A description of any assumptions or corrections, such as tests of normality and adjustment for multiple comparisons
☐	☒	A full description of the statistical parameters including central tendency (e.g. means) or other basic estimates (e.g. regression coefficient) AND variation (e.g. standard deviation) or associated estimates of uncertainty (e.g. confidence intervals)
☐	☒	For null hypothesis testing, the test statistic (e.g. *F*, *t*, *r*) with confidence intervals, effect sizes, degrees of freedom and *P* value noted<br>*Give P values as exact values whenever suitable.*
☒	☐	For Bayesian analysis, information on the choice of priors and Markov chain Monte Carlo settings
☒	☐	For hierarchical and complex designs, identification of the appropriate level for tests and full reporting of outcomes
☒	☐	Estimates of effect sizes (e.g. Cohen's *d*, Pearson's *r*), indicating how they were calculated

Our web collection on statistics for biologists contains articles on many of the points above.

Software and code

Policy information about availability of computer code

Data collection	ESR data : Bruker WINEPR system v2.22; Microcal OriginPro v2022b; OSL data : Analyst v.4.57; R Statistical Software (v4.2.2) with RLumShiny (v1.8.1.1) and Luminescence v 0.9.26 R code packages; Pollen data : R studio v4.2.2 and 'wesanderson' package v0.3.6; Pytholiths data : C2 v1.8.0; Sedimentology data : Gradistat v8; Biomarker data: PAST 4.03 statistical software; MassHunter Qualitative Analysis v.8.0<br>Map : ArcMap v10.5; BartSoft v4.2;
Data analysis	The data analysis (dating, sedimentology, istopics and biomarkers) are precisely described and provided in the Supplementary Information, Methods online and Extended Data Figures.

For manuscripts utilizing custom algorithms or software that are central to the research but not yet described in published literature, software must be made available to editors and reviewers. We strongly encourage code deposition in a community repository (e.g. GitHub). See the Nature Portfolio guidelines for submitting code & software for further information.

Data

Policy information about availability of data

All manuscripts must include a data availability statement. This statement should provide the following information, where applicable:

- Accession codes, unique identifiers, or web links for publicly available datasets
- A description of any restrictions on data availability
- For clinical datasets or third party data, please ensure that the statement adheres to our policy

Provide your data availability statement here.

Research involving human participants, their data, or biological material

Policy information about studies with human participants or human data. See also policy information about sex, gender (identity/presentation), and sexual orientation and race, ethnicity and racism.

Reporting on sex and gender	NA
Reporting on race, ethnicity, or other socially relevant groupings	NA
Population characteristics	NA
Recruitment	NA
Ethics oversight	NA

Note that full information on the approval of the study protocol must also be provided in the manuscript.

Field-specific reporting

Please select the one below that is the best fit for your research. If you are not sure, read the appropriate sections before making your selection.

☐ Life sciences ☐ Behavioural & social sciences ☒ Ecological, evolutionary & environmental sciences

For a reference copy of the document with all sections, see nature.com/documents/nr-reporting-summary-flat.pdf

Ecological, evolutionary & environmental sciences study design

All studies must disclose on these points even when the disclosure is negative.

Study description	We report a clear association between late Middle Pleistocene material culture and a wet tropical forest in southern Côte d'Ivoire. Twinned Optically Stimulated Luminescence (OSL) and Electron Spin Resonance (ESR) dating methods constrain the onset of human occupations at Bété I to ~150 ka. Plant wax biomarker, stable isotope, phytolith and pollen analyses of associated sediments all point to a wet forest environment.
Research sample	Sediment samples from Bété I site used to generate chronometric dates (SA and SG ages : 8; Ti-H ESR ages : 5), 37 sediments characterized, 37 bulk isotopes analysis, 31 sediments for plant wax analyses, 9 sediments for phytoliths and pollen
Sampling strategy	Sediment sampled to cover the first ca. 6m of the sedimentary sequence of Bété I.
Data collection	Fieldtrip in March 2020.
Timing and spatial scale	Not relevant.
Data exclusions	*If no data were excluded from the analyses, state so OR if data were excluded, describe the exclusions and the rationale behind them, indicating whether exclusion criteria were pre-established.*
Reproducibility	Not relevant.
Randomization	Not relevant.
Blinding	Not applicable to palaeontology/archaeology.

Did the study involve field work? ☒ Yes ☐ No

Field work, collection and transport

Field conditions	Bété I is located in the present-day tropical forest and excavations in March 2020 (hot and humid).
Location	Anyama city, ca. 20 km North of Abidjan.
Access & import/export	*Describe the efforts you have made to access habitats and to collect and import/export your samples in a responsible manner and in compliance with local, national and international laws, noting any permits that were obtained (give the name of the issuing authority, the date of issue, and any identifying information).*
Disturbance	*Describe any disturbance caused by the study and how it was minimized.*

Reporting for specific materials, systems and methods

We require information from authors about some types of materials, experimental systems and methods used in many studies. Here, indicate whether each material, system or method listed is relevant to your study. If you are not sure if a list item applies to your research, read the appropriate section before selecting a response.

Materials & experimental systems

n/a	Involved in the study	
☒	☐	Antibodies
☒	☐	Eukaryotic cell lines
☐	☒	Palaeontology and archaeology
☒	☐	Animals and other organisms
☒	☐	Clinical data
☒	☐	Dual use research of concern
☒	☐	Plants

Methods

n/a	Involved in the study	
☒	☐	ChIP-seq
☒	☐	Flow cytometry
☒	☐	MRI-based neuroimaging

Palaeontology and Archaeology

Specimen provenance	NA
Specimen deposition	NA
Dating methods	Optically Stimulated Method (OSL) and Electron Spin Resonance (ESR) on quartz

☐ Tick this box to confirm that the raw and calibrated dates are available in the paper or in Supplementary Information.

Ethics oversight	No ethical approval was required

Note that full information on the approval of the study protocol must also be provided in the manuscript.

Plants

Seed stocks	NA
Novel plant genotypes	NA
Authentication	NA
